# Supplementary material for: Diverse behaviors in non-uniform chiral and non-chiral swarmalators
Source: Nat Commun. 2023 Feb 20;14:940. doi: 10.1038/s41467-023-36563-4 (PMC9941214; doi:10.1038/s41467-023-36563-4)
Supplement: Supplementary file 1 — Supplementary Materials File [file 41467_2023_36563_MOESM1_ESM.pdf]

Supplementary Information for

# Diverse Behaviors in Non-Uniform Chiral and Non-Chiral Swarmalators

## The PDF file includes:


Supplementary Table 1. Summary of emergent states in this work and other works.

Supplementary Note 1. System oscillation characterization.
Supplementary Note 2. Natural frequency and revolution radius distributions.
Supplementary Note 3. Non-chiral swarmalators with a frequency spread.
Supplementary Note 4. Overview and characterization of non-chiral swarmalators.
Supplementary Note 5. Chiral swarmalators with discrete sets of natural frequencies.
Supplementary Note 6. Overview and characterization of chiral swarmalators.
Supplementary Note 7. Order Parameter.
Supplementary Note 8. Analysis of sync state.
Supplementary Note 9. Analysis of Phase waves / vortices.
Supplementary Note 10. Analysis of Revolving Swarmalators.

Supplementary Fig. 1. Summary of emergent states.
Supplementary Fig. 2. Phase-interaction parameter space.
Supplementary Fig. 3. Natural frequency distributions.
Supplementary Fig. 4. Non-chiral swarmalators with a natural frequency spread.
Supplementary Fig. 5. Collective behaviors of non-chiral swarmalators with no frequency coupling.
Supplementary Fig. 6. Collective behavior characterization of non-chiral swarmalators with discrete natural frequencies and no frequency coupling.
Supplementary Fig. 7. Collective behavior characterization of non-chiral swarmalators with no frequency coupling and a natural frequency spread.
Supplementary Fig. 8. Splintered phase waves
Supplementary Fig. 9. Frequency group separation.
Supplementary Fig. 10. Concentric phase self-organization.
Supplementary Fig. 11. Collective expansion.
Supplementary Fig. 12. Collective behaviors of non-chiral swarmalators with frequency coupling.
Supplementary Fig. 13. Collective behaviors of chiral swarmalators with no frequency coupling.
Supplementary Fig. 14. Chiral swarmalators with no natural frequency spread.
Supplementary Fig. 15. Characterization of collective behaviors in chiral swarmalators with discrete natural frequencies and no frequency coupling.
Supplementary Fig. 16. Characterization of collective behaviors in chiral swarmalators with a natural frequency spread and no frequency coupling.
Supplementary Fig. 17. Collective behaviors of chiral swarmalators with frequency coupling.
Supplementary Fig. 18. Characterization of collective behaviors in chiral swarmalators with frequency coupling.
Supplementary Fig. 19. Order parameters.
Supplementary Fig. 20. Collective behaviors of locally coupled non-chiral swarmalators with one natural frequency and no frequency coupling.
Supplementary Fig. 21. Collective behaviors of locally coupled non-chiral swarmalators with two natural frequencies and no frequency coupling.
Supplementary Fig. 22. Collective behaviors of locally coupled non-chiral swarmalators with a natural frequency spread and no frequency coupling.

Supplementary Fig. 23. Collective behaviors of locally coupled chiral swarmalators with one natural frequency and no frequency coupling.
Supplementary Fig. 24. Collective behaviors of locally coupled chiral swarmalators with two natural frequencies and no frequency coupling.
Supplementary Fig. 25. Collective behaviors of locally coupled chiral swarmalators with a natural frequency spread and no frequency coupling.
Supplementary Fig. 26. Collective behaviors of locally coupled chiral swarmalators with no natural frequency spread and frequency coupled.
Supplementary Fig. 27. Collective behaviors of locally coupled chiral swarmalators with a natural frequency spread and frequency-coupled.
Supplementary Fig. 28. 3D Maps for locally coupled non-chiral swarmalators with no frequency spread and no frequency coupling.
Supplementary Fig. 29. 3D Maps for locally coupled non-chiral swarmalators with a frequency spread and no frequency coupling.
Supplementary Fig. 30. 3D Maps for locally coupled chiral swarmalators with no frequency spread and no frequency coupling.
Supplementary Fig. 31. 3D Maps for locally coupled chiral swarmalators with a frequency spread and no frequency coupling.
Supplementary Fig. 32. 3D Maps for locally coupled non-chiral swarmalators with no frequency spread and frequency-coupled.
Supplementary Fig. 33. 3D Maps for locally coupled non-chiral swarmalators with a frequency spread and frequency-coupled.
Supplementary Fig. 34. 3D Maps for locally coupled chiral swarmalators with no frequency spread and frequency-coupled.
Supplementary Fig. 35. 3D Maps for locally coupled chiral swarmalators with a frequency spread and frequency-coupled.
Supplementary Fig. 36. Cluster count and motion characterization of locally coupled chiral swarmalators with no frequency spread and no frequency coupling.
Supplementary Fig. 37. Cluster count and motion characterization of locally coupled chiral swarmalators with a frequency spread and no frequency coupling.
Supplementary Fig. 38. Cluster count and motion characterization of locally coupled chiral swarmalators with no frequency spread and frequency-coupled.
Supplementary Fig. 39. Cluster count and motion characterization of locally coupled chiral swarmalators with no frequency spread and frequency-coupled.
Supplementary Fig. 40. Sync state for increasing $d$.
Supplementary Fig. 41. Phase coherence for increasing $d$ in collectives with global coupling, identical, and non-chiral swarmalators.
Supplementary Fig. 42. Phase-phase correlation function.
Supplementary Fig. 43. Velocity auto-correlation function.
Supplementary Fig. 44. Sync state for increasing Ω.
Supplementary Fig. 45. Phase coherence for increasing $d$ in collectives with global coupling, non-identical, and non-chiral swarmalators.
Supplementary Fig. 46. Non-monotonic behavior of phase coherence for collectives with global coupling, non-identical, and non-chiral swarmalators.
Supplementary Fig. 47. Phase-phase correlation function for collectives with global coupling, non-chiral, and non-identical swarmalators.
Supplementary Fig. 48. Non-monotonic behavior of phase coherence for collectives with local coupling and non-chiral swarmalators.
Supplementary Fig. 49. Sync state for different $\sigma$ when there is local coupling.
Supplementary Fig. 50. $g(r)$ for different coupling ranges $\sigma$.

Supplementary Fig. 51. $g(r)$ for different coupling ranges $\sigma$ when noise increases.
Supplementary Fig. 52. Phase-phase correlation of collectives with local coupling ($\sigma = 10, 3, 1$), non-identical, non-chiral, and with noise.
Supplementary Fig. 53. Phase-phase correlation of collectives with local coupling ($\sigma = 10, 3, 1$), non-identical ($\Omega = 0.9$), non-chiral, and with noise.
Supplementary Fig. 54. Vortices with disorder.
Supplementary Fig. 55. Order parameter s for different $d$ and Ω.
Supplementary Fig. 56. Phase-phase correlation and $g(r)$ for vortices.
Supplementary Fig. 57. Phase-phi space for phase wave state.
Supplementary Fig. 58. Order parameters for active and quenched disorder.
Supplementary Fig. 59. Phase-phase correlation and $g(r)$ for revolving collectives in the vortex state.
Supplementary Fig. 60. Comparison to embryonic genetic oscillators.
Supplementary Fig. 61. Local clusters of embryonic genetic oscillators.
Supplementary Fig. 62. Synchronization of various clusters.
Supplementary Fig. 63. Comparison to experiments with micro-disks.
Supplementary Fig. 64. Phase difference vs. spatial orientation difference.

**Other Supplementary Material for this manuscript includes the following:**

Supplementary Movie 1. Non-Chiral Swarmalators with No Frequency Coupling.
Supplementary Movie 2. Splintered Phase Waves.
Supplementary Movie 3. Natural Frequency Group Separation.
Supplementary Movie 4. Concentric Phase Self-Organization.
Supplementary Movie 5. Non-Chiral Swarmalators with Frequency Coupling.
Supplementary Movie 6. Revolving swarmalators with No Frequency Coupling.
Supplementary Movie 7. Revolving Swarmaltors with Frequency Coupling.
Supplementary Movie 8. Global coupling, non-chiral, and noise.
Supplementary Movie 9. Global coupling, non-identical, and non-chiral.
Supplementary Movie 10. Local coupling, identical, and non-chiral.
Supplementary Movie 11. Global coupling, non-identical, and non-chiral vortices with noise.
Supplementary Movie 12. Global coupling, identical, and non-chiral vortices with noise.
Supplementary Movie 13. Local coupling, non-chiral, and no frequency coupling.
Supplementary Movie 14. Local coupling, chiral, and no frequency coupling.
Supplementary Movie 15. Local coupling, chiral, and frequency coupling.
Supplementary Movie 16. Slime Mold.
Supplementary Movie 17. Embryonic Genetic Oscillators.

**Supplementary Table 1. Summary of emergent states in this work and other works.**
*A refers to the work in this paper

| No. | States | Real-world systems that exhibit similar states | Referenced studies that demonstrate similar states | | | | | | | |
|---|---|---|---|---|---|---|---|---|---|---|
| | | | A | 20 | 24 | 25 | 26 | 27 | 36 | 45 |
| 1 | Static Sync | Spermatozoa[10], bristlebots[56], Janus particles[57], chemical micromotors[58, 59], magnetic domain walls[60] | ■ | | ■ | | | ■ | | ■ |
| 2 | Static Async | Quincke rollers[31], bristlebots[56] | ■ | | ■ | | | ■ | | |
| 3 | Phase Wave | Ferromagnetic colloids[61], vinegar eels[11], Japanese tree frogs[5] | ■ | | ■ | | | ■ | | |
| 4 | Splintered Phase Wave | ~ | ■ | | | | | ■ | | |
| 5 | Active Phase Wave | Mosh pit[62], Colloidal rollers[31], Spermatozoa[10], Bristlebots[56] | ■ | | | | | ■ | | |
| 6 | Linear Phase Wave | Slime mold[47] | ■ | | ■ | | | | | |
| 7 | Multiple Bouncing Clusters | ~ | ■ | | ■ | | | | ■ | |
| 8 | Concentric Phase Self-Organization | Embryonic genetic oscillators[40] | ■ | | | | | | | ■ |
| 9 | Radial Oscillation | Phototactic micromotors[12] | ■ | | | | | | | |
| 10 | Synchronized Expansion | Ferromagnetic microrobots[53] | ■ | | | | | | | |
| 11 | Anti-Phase State | ~ | ■ | | | | | | | |
| 12 | Vortex | Mosh pit[62], ferromagnetic microrobots[49, 50, 53, 54] | ■ | | | | | | | |
| 13 | Gas-like | Ferromagnetic microrobots[53], Quincke rollers[31], slime mold[47] | ■ | ■ | | ■ | ■ | | | |
| 14 | Dense Revolving Cluster | Bristlebots[56] | ■ | | | | ■ | | | |
| 15 | Multiple Revolving Cluster | Bristlebots[56], Quincke rollers[31] | ■ | ■ | | ■ | ■ | | | |
| 16 | Sparse Revolving Cluster | Quincke rollers[31] | ■ | ■ | | | ■ | | | |
| 17 | Multiple Vortices | Spermatozoa[10] | ■ | | | | | | | |
| 18 | Elongated Vortices | ~ | ■ | | | | | | | |

## Supplementary Note 1. System oscillation characterization

This discussion reviews the oscillatory swarmalator behavior in attraction and repulsion across various phase interaction parameter spaces. The plots in Supplementary Fig. 2 show why oscillation in attraction and repulsion occurs as the agents' phases vary. Fig. S1a shows a heat map of the spatial phase interaction term in Equation (1) from the main text: when the two agents' phases are used as coordinates to plot a point in $\theta_i - \theta_j$ space, we can determine whether that point lies along attraction or repulsion regions. If the point lies close to the line $\theta_j = \theta_i$, then it lies within the yellow region which indicates a positive value for the spatial phase interaction, which enables attraction. When the point lies close to either of the lines $\theta_j = \theta_i - \pi$ or $\theta_j = \theta_i - \pi$, then the spatial phase interaction is negative, which enables repulsion. If agents vary their phases at the same rate, they will maintain the same phase difference over time and according to the phase distribution they will maintain a constant amount of attraction or repulsion. However, with natural frequency distributions the phase difference changes over time and the ($\theta_i$,$\theta_j$) point moves through attraction and repulsion regions.

Supplementary Fig. 2b shows the effect of a phase offset on the spatial phase interaction term. Here, the space is defined by the phase difference changing between $-2\pi$ and $2\pi$ and a fixed phase offset ($Q_{\dot{x}}$) also changing between 0 and $2\pi$; the trend shown continues over larger ranges. In Supplementary Fig. 2a there were two bands of repulsion and one band of attraction; however, the phase offset enables two additional bands, three bands of repulsion and two bands of attraction. The more complex space means that agents are more prone to switch between attraction and repulsion when their phase difference changes over time. Supplementary Fig. 2c shows the spatial phase interaction value when $Q_{\dot{x}}$ is defined by Equation (4) from the main text. Here, the three possible values for $Q_{\dot{x}}$ are $-\pi$, 0, and $\pi$.

The frequency dependence is relevant when modeling physical systems, where agents with the same direction of motion will tend to align and repel agents that are revolving in the opposing direction. The three lines show the phase shifts along positive and negative phase differences that occur with frequency coupling. Future swarmalator studies with different natural frequency distributions may find the particular spatial phase interaction space that leads to especially useful static or dynamic formations. Further research along these lines may find that macro-scale robot collectives can be made to move in specific ways and self-organize into any formation by simply communicating some phase value to surrounding agents and reacting to the neighbors' phase through a programmable spatial phase interaction.

**Supplementary Note 2. Natural frequency distributions.**

This discussion reviews the methods used for generating the four natural frequency distributions used in this study and how they relate to the inherent orbiting radius when $c_i$ is equal to $1$ or $-1$.

- Frequency distribution $F1$: The collective shares the same natural frequency ($\omega = 1$), $c_i = 1$ and $R_i = 1$.
- Frequency distribution $F2$: The collective is split into two natural frequency groups; half of the collective's natural frequency is $\omega = 1$ and the second half has $\omega = -1$. Here, the first half has $c_i = 1, R_i = 1$ and the other half with $c_i = -1, R_i = 1$.
- Frequency distribution $F3$: There is a spread of natural frequencies across only positive values so that the whole collective has $\omega \sim U(1,3)$, $c_i = 1$, and $P(R) \sim R\text{^}(-2)$.
- Frequency distribution $F4$: There is a spread of natural frequencies across only positive values so that one half of the swarmalators have $\omega \sim U(1,3)$ and the second half have $\omega \sim U(-3,-1)$. If the swarmalators have inherent circular orbits, then first half of the collective has $c_i = 1$ and the second half has $c_i = -1$, and both halves have $P(R) \sim R\text{^}(-2)$.

For chiral swarmalators and frequency-coupled chiral swarmalators (FCCS) this ensures that agents have a negative or positive sign to their natural frequency and an inherent angular velocity in their circular orbit which determines their direction of motion. For revolving swarmalators $R_i$ is a physically relevant term since it defines an agent's revolution radius.

The four natural frequency distributions ($F1$, $F2$, $F3$, and $F4$) are depicted in Supplementary Fig. 3.

## Supplementary Note 3. Non-chiral swarmalators with a natural frequency spread

This discussion presents some of the most interesting emergent collective behaviors when non-chiral swarmalators have a natural frequency spread. Supplementary Figs. 4a-b show that disorder lies throughout much of the $K - J$ parameter space and an example formation is shown in Supplementary Fig. 4c. At higher values of $K$ and $J$, however, clusters of agents with lower $|\omega|$ begin to synchronize while agents with higher $|\omega_i|$ remain asynchronous and at the outer edges of the collective. Supplementary Fig. 4d shows the first instance of partial synchronized collectives with a positive natural frequency spread; at $K = 1$, the collective begins to couple and initially a small, synchronized cluster forms in the center of a ring of asynchronous agents, much like the one in Supplementary Fig. 4e. Over time, agents from the asynchronous group begin to couple more closely with the cluster and therefore move towards the center. The agents joining the center cluster have a higher natural frequency; therefore, the group does not synchronize and an elongated phase wave forms along an axis. Agents from the outer boundary coalesce at both ends of the phase wave, slowly stepping into phase with the surrounding agents.

Supplementary Fig. 4e shows synchronization; Similar behaviors occur when the natural frequency spread is across positive and negative values, except two sync clusters form since agents with the same sign of natural frequency can synchronize more easily. As shown in the sequential images in Supplementary Fig. 4f, two phase waves periodically bounce from each other's boundary because of the oscillatory attraction and repulsion and are surrounded by a cloud of agents with higher natural frequencies. The mechanisms of phase interaction here are like those underlying the behavior in main text Fig. 2h, but the spatial oscillation amplitude remains much lower because of the many agents along the border that are unable to join one of the synchronized clusters. Another partial synchronization state emerges at high $K$ when $J = 0$ for both natural frequency distributions; agents coalesce into a circular formation and many of them synchronize (Supplementary Figs. 4g-h).

Finally, a state emerges at high $K$ and low $J$; agents self-organize concentrically by phase and an asynchronous cluster forms at the center. When $J < 0$, agents with a high $|\omega_i|$ spatially attract since their phase difference is large much of the time, thus a cluster forms. Conversely, agents with a lower $|\omega_i|$ synchronize more easily and repel each other because of the low phase difference; this drives them to form a sparse cloud around the center cluster (Supplementary Figs. 4i-j). A greater fraction of the collective forms the sparse cloud in Supplementary Fig. 4i than in Supplementary Fig. 4j because all agents have natural frequencies distributed across positive values, which enables a greater fraction to have low $|\omega_i|$ and synchronize more easily. In Supplementary Movie 1 there is slight radial oscillation; however, we classify these as separate states because (1) the radial oscillation is almost insignificant, (2) much of the collective remains clustered and is not expanding, and (3) the perimeter cloud of agents has a noticeable concentric phase wave traveling from the center out that is not as obvious in the radially oscillating collective from main text Fig. 2j.

## Supplementary Note 4. Overview and characterization of non-chiral swarmalators

This discussion reviews the collective behaviors exhibited by non-chiral swarmalators with no frequency coupling. The behaviors achieved by swarmalators when $c, Q_{\dot{x}}, Q_{\dot{\theta}} = 0$ for integer values of $K$ and $J$ are shown for the four natural frequency distributions in Supplementary Fig. 5. The formations shown here have only scratched the surface of what emerges when the coefficients within $\dot{\boldsymbol{x}}_i$ and $\dot{\theta}_i$ are varied. The formations shown in Supplementary Fig. 6 correspond to swarmalators with discrete natural frequencies; Supplementary Figs. 6a-g help the reader visualize each of the corresponding behavioral characterizations that are relevant in Supplementary Figs. 6h-n.

The first formation shown and characterized in Supplementary Figs. 6a and 6h is the interacting phase waves that results from half of the collective having $\omega_i = -1$ and the other half $\omega_i = 1$. The mean speed and $S$ order are plotted for the range of $K$ when $J = 1$. Throughout most of the cases in which $K > 0$, the collective holds a stable mean speed between 0.1 and 0.15; however, around the point where two interacting phase waves emerge ($K \approx 0$), the collective increases its mean speed because of the increased oscillation that occurs when the agents spread out along rings and are each closer to agents from the opposite natural frequency group. When $K, J > 0$, two clusters form that oscillate between attraction and repulsion; this enables the entire collective to be in constant motion, and after each natural frequency group synchronizes within itself, the speed remains constant ($K \approx 0.3$). At these higher values of $K$, the cluster formation ensures that each agent's motion is affected mainly by two values at any point in time: the average phase within its own natural frequency group and the average phase of the opposite group. When the ring formations emerge, the global spatial-phase order decreases because of the presence of two natural frequency groups, but the concurrent interaction between so many different phases enables each agent to oscillate in place more quickly.

In Supplementary Fig. 6i the local coherence measures the average phase coherence of each agent with neighbors that are within a distance of $0.15$. The static anti-phase locked state occurs when $J = 0$; when $K < 0$, the local coherence remains at a very small value and then spikes up as soon as it is positive. Another increase in phase coherence occurs when $K > 1.5$; here, the agents synchronize with most other members within the same natural frequency group.

Supplementary Fig. 6j shows the amplitude of the distance between the centroids of the two natural frequency groups throughout $K - J$ space. The amplitude is highest when two clusters form and "bounce" off each other's circumference ($K, J > 0$). At high values of $K$, ($K \approx 2$) the whole collective begins to synchronize which halts the bouncing cluster behavior and forms the sync state that has been observed in earlier works[27]. We have run longer simulations with lower values of $K$ for this natural frequency distribution and have found that the bouncing clusters continue oscillating about their common centroid with the same amplitude. We demonstrate that clustering also occurs when there are more than two natural frequency groups. A collective of $500$ agents with random phases are evenly divided among the following five natural frequencies: $-1$, $-0.5$, $0$, $0.5$, and $1$. When $K, J = 1$, five independently synchronous clusters form that oscillate between attraction and repulsion with the surrounding clusters. This behavior is shown in Supplementary Movie 2 and the five separate clusters in Supplementary Fig. 9a. Supplementary Figs. 9b-f show the oscillation between each natural frequency group's centroid as it oscillates between attraction and repulsion with each of the others. Since there are more than two groups, the oscillation is noisy as the frequency groups in the center are squeezed out of the visible range of the axis along which the groups align. We also show images of collectives with three, four, five, and nine natural frequency groups in Supplementary Fig. 9g-j and display each of their bouncing behaviors in Supplementary Movie 2. We believe this emergent behavior could be especially relevant to robot collectives at the macro scale that can separate and cluster into distinct groups simply by changing their natural frequencies.

The expanding and contracting collectives are analyzed in Supplementary Fig. 6k where the collective's radii is recorded for the peak minimum and maximum as it oscillates. When $K < 0, J = -1$, agents cannot synchronize and as a result there is minimal repulsion across the collective since few agents share the same phase. As soon as $K > 0$, the collective increases its degree of synchrony within each natural frequency group, and agents repel each other when they share the same phase. Agents with $\omega_i = -1$ are essentially traveling about the phase unit circle in the clockwise direction while the agents with $\omega_i = 1$ are traveling in the counterclockwise direction. Since both groups are traveling at the same rate about the phase unit circle in opposite directions, they exhibit the same phase two times for each phase oscillation cycle. The repulsion between the two groups is highest when the whole collective shares the same phase, which results in the maximum point for the radial oscillation cycle. The greatest attraction occurs when the phases of the two natural frequency groups are at opposite ends of the phase unit circle; this results in the minimum collective radius for the radial oscillation cycle.

Supplementary Fig. 6e shows the phase wave behavior that results when the natural frequency distribution is $F1$ and $K = 0, J = 1$. This is very similar to what was shown in 2017 by O'Keeffe et al[27]. with the difference that although each agent remains essentially unmoving once the phase wave formation is reached, the whole collective oscillates in place so that a uniform phase wave oscillates circumferentially about the annulus formation. In the original swarmalator study, the collective was shown either in a static phase wave state where the agents' motion and phase remained static, or in an active phase wave state where their phase and motion were dynamic. The phase wave state achieved in our study results from the fact that the natural frequency for all agents is much higher than what is needed for phase-coupling-induced motion given by the spatial phase coupling term in $\dot{\boldsymbol{x}}_i$.A lower natural frequency would similarly produce the active phase wave state. Supplementary Fig. 6l shows the spatial-phase order throughout the collective for different values of $K$ and $J$; these results allow us to get a closer look at the behavior of the order parameter $S$ throughout $K - J$ parameter space than what is shown in the main text.

The collective reaches another state where degree of synchrony increases with distance from the collective's centroid when $F1$ and $K = 0, J = -1$, as shown in Supplementary Figs. 6f and 5m. As opposed to exponential-like and step-like behavior in degree of synchrony as a function of increasing distance from the collective centroid seen for collectives with natural frequency distributions $F3$ and $F4$, the plot shown in Supplementary Fig. 6m shows a very linear behavior. The linear behavior is the same when $K = 0.5, 1, 1.5,$ and 2, and reaches a maximum degree of synchrony value of $\sim 0.2$. From Supplementary Fig. 6f we can distinctly see a ring of agents with similar phases lining the circular boundary of the collective.

Supplementary Fig. 6g shows an expanding collective when $F1$ and $K = 2, J = -1$; since $J$ is negative, agents with similar phases will repel and expand the radius of the collective. Supplementary Fig. 6n shows the rate of expansion and the final radius (after 500 time steps) for different values of $K$ when $J = -1$. After $K$ reaches a high enough value to synchronize agents' phases, radial repulsion begins, and the collective maintains the same rate of expansion and final radius after 500 time steps. Supplementary Fig. 9 explores the expansion behavior across 1000 time steps for different values of $J$; this plot demonstrates that when $0 > J > -1$, the collective expands and reaches a steady collective radius at which it remains. When $J = -1$ the collective continues expanding and does not seem to stop; this is reasonable since the phase coupling-induced motion in $\dot{\boldsymbol{x}}_i$ is defined by a unit vector and the coefficient for global attraction ($A$) has a value of 1. When $J = -1$ and all agents are synchronized, the unit vector term in $\dot{\boldsymbol{x}}_i$ is cancelled out and only the power law repulsion term remains active; thus, all agents repel each other slightly and the collective expands indefinitely.

When the natural frequency distribution is $F4$, the collective has a uniform distribution of natural frequencies across positive and negative values. This ensures that agents with the same natural frequency sign will not synchronize as easily as when the natural frequency distribution

is $F2$. The emergent behaviors for collectives with natural frequency distributions are shown in Supplementary Figs. 7a-d and characterized in Supplementary Figs. 7e-h. The first emergent collective behavior presented for this natural frequency distribution is shown in Supplementary Fig. 7a ($K = 2, J = -1$), where a cluster of phase-disordered agents forms at the center and is surrounded by rings of agents with greater phase synchrony. Supplementary Fig. 7e explores the phase synchrony behavior as a function of distance from the collective's centroid for different positive values of $K$. At $K = 0, 0.5,$ and 1, the degree of synchrony remains below $0.05$ across all distances from the centroid. Once $K \approx 1.5$, there is a jump in the degree of synchrony at about $0.7R_{max}$ and which increases with distance from the collective centroid ($R_{max}$ is the collective radius). These periodic rings of higher synchrony occurs because of the spread in natural frequency; agents with a higher $|\omega_i|$ will have a harder time synchronizing their phase with their neighbors. Since $J$ is negative in these cases, agents with greater phase differences will tend to attract to each other and cluster closer to the collective centroid. Agents with a lower $|\omega_i|$ will synchronize more easily with other agents that share the same sign for their natural frequency; this means that these agents will repel each other more and move towards the collective's boundary where they will have the greatest distance from similarly phased agents. Agents with positive and negative natural frequencies will have high phase differences which enables greater attraction. As a result, although the degree of synchrony increases the collective boundary, it does not surpass $0.1$ because agents with differing phases attract and sometimes occupy the same annulus regions used for the measurements in this plot.

Supplementary Fig. 7b ($K = 2, J = 1$) shows two clusters of agents form close to the collective centroid while a ring of phase-disordered agents encapsulate the clusters. Fig. 5f shows how the degree of synchrony for the whole collective and within each natural frequency group increases with $K$. This shows that higher values of $K$ are needed to fully synchronize each group and the whole collective. Similar bouncing cluster behavior is also observed for $K, J > 0$; however, the clusters do not repel each other at such great distances as was the case when $F2$ since there is greater phase disorder resulting from the spread of natural frequencies.

When $F3$, the collective has a uniform distribution between 0 and 1 for the agents' natural frequencies. Supplementary Fig. 7c shows the collective almost forming a sync state when $K = 2, J = 1$; however, a tight ring of asynchronous agents encapsulate the center cluster. The outer agents have higher natural frequency which makes it more difficult for them to synchronize with the center agents. Since $J$ is positive, agents with similar phases attract to each other; therefore, the outer agents tend to radially oscillate as their phase difference with the center cluster increases or decreases. In Supplementary Fig. 7g, we show that the collective synchrony increases with $K$, but only reaches about $0.6$ because of the spread in natural frequency.

When $K = 2, J = -1$ (Supplementary Fig. 7d), the collective exhibits a more organized version of what emerges at the same values of $K$ and $J$ when the natural frequency is spread across negative and positive values. A tight cluster of phase disordered agents forms at the center while rings of more coherent agents encapsulate it. The agents along the boundary have a lower natural frequency than those in the cluster and thus synchronize more easily. Supplementary Fig. 7h shows how the coherence within concentric rings increases with the distance from the collective's centroid. Starting at about $K = 1.5$ the degree of synchrony begins to rise at a distance of $\sim 0.4$ from the collective centroid. Since all agents share the same sign for their natural frequencies, there are lower phase differences between agents with a lower $|\omega|$ which enables greater spacing between the phase-ordered agents and thus a larger region of phase-ordered agents than when $F2$. Supplementary Fig. 10 and Supplementary Movie 4 shows a dynamic plot of the agents' distance from the collective centroid as a function of phase, and highlights the concentric phase self-organization behavior for different $J$.

## Supplementary Note 5. Chiral swarmalators with discrete sets of natural frequencies

This discussion reviews the chiral collective's behavior when all agents revolve in the same direction at the same rate and when half revolve clockwise (CW) and the other half counterclockwise (CCW) at the same rate. The emergent formations across $K$ and $J$ in the four natural frequency distributions is shown in Supplementary Fig. 13; however, only the upper left and lower right quadrants of the figure relate to this discussion. A summary of the most interesting behaviors and the $S$ order heat maps across $K - J$ parameter space is shown in Supplementary Fig. 14. Supplementary Figs. 14a-b shows that $S$ remains relatively high even when $K$ and $J$ are negative; a very disordered phase wave forms along this region (Supplementary Fig. 14c) with high spatial-phase order at the outer boundary and low order towards the center. The circular motion and negative $K$ drives agents to follow circular paths that will intersect where the phase difference is greatest. Agents on opposite sides of the collective, revolving in the same direction, minimize the distance between each other when they are offset by $\pi$ along their circular trajectories, and results in the emergent formation in Supplementary Fig. 14c.

Disordered phase waves also exist at low $J$ and high $K$; Supplementary Fig. 14d shows a phase wave along an axis that results from a rotating ellipse formation that steadily increases its major axis over time. In this case, similar phases spatially attract, but temporally repel; instead of moving towards synchrony, agents anti-couple and become asynchronous. This enables the formation to slowly stretch out over time. Similar elliptical formations are observed when the collective has two natural frequency groups; however, the two ellipses that emerge rotate about a common center in opposite directions and maintain relatively constant major axes over time. The equal and opposite natural frequencies inhibit perfect anti-coupling, so the collective maintains a steady rotating formation over time (Supplementary Movie 6).

Like the non-chiral swarmalators, distinct phase waves emerge when $K = 0$ (Supplementary Figs. 14e-g). With two natural frequency groups, $K = 0,\ J = 1$ enables agents to separate into concentric, counter-rotating groups that each oscillate between being on the inside and being on the outside (Supplementary Fig. 14f). This is driven by their spatial repulsion and their attempt to each settle at the same revolution radius. A phase wave also forms when there is no spatial or temporal coupling (Supplementary Fig. 14g), the collective forms an annulus because of the agents' inherent circular motion with equal radii, their spatial attraction through the unit vector model, and repulsion through a power law model. All agents maintain the same radius of revolution, but switch between the inner and outer boundaries of the annulus.

Sync states are also observed at positive $K$ and $J$ when the collective revolves in the same direction (Supplementary Fig. 14h); and a high $S$ is maintained because the collective maintains high circumferential phase order about its time-average centroid. Similar behavior is observed when there are two natural frequency groups; two clusters independently synchronize and revolve along the same circular trajectory in opposite directions (Supplementary Fig. 14i). Contrary to the bouncing clusters observed for the non-chiral swarmalators, these collectives maintain a steady trajectory and pass through each other two times per phase cycle because of their inherent circular motion. Their inherent motion practically inhibits synchronization since agents from opposite natural frequency groups have a harder time clustering. Finally, expanding synchronized collectives are also found for chiral swarmalators when the collective has uniform revolution behavior (Supplementary Fig. 14j) and sparse revolving clusters when there are two natural frequency groups (Supplementary Fig. 14k). The sparse revolving clusters are very similar to the dense revolving clusters, except that there is much higher neighbor spacing. Each cluster size also remains relatively constant, as opposed to constant expansion (Supplementary Fig. 14j) or radial oscillation (main text Fig. 2k), because of the inherent circular motion that inhibits much of the phase interaction effects between the two groups.

## Supplementary Note 6. Overview and characterization of chiral swarmalators

This discussion reviews the collective behaviors of regular and frequency-coupled chiral swarmalators. The behaviors of collectives composed of chiral swarmalators with no natural frequency coupling ($c = 1, Q_{\dot{x}}, Q_{\dot{\theta}} = 0$) when $K$ and $J$ hold integer values and different natural frequency distributions are shown in Supplementary Fig. 14. Supplementary Figs. 15a-e show many of the emergent behaviors when the collective has discrete natural frequencies and each behavior is characterized in Supplementary Figs. 15f-j.

Supplementary Fig. 15a highlights chiral swarmalators' emergent behaviors when there is no phase coupling and only global attraction enabled by a unit vector model and repulsion through a power law. The phase wave formation results from the fact that all agents have the same angular velocity and thus the same radius of revolution; half have an inherent clockwise motion and the other half a counterclockwise motion. Since $K, J = 0$, there is no phase coupling behavior and agents simply revolve about the same centroid. The plot of $S$ in Supplementary Fig. 15f shows a clearer view of the spatial-phase order when $J = 0$ than the heat map shown in the main text. As opposed to non-chiral swarmalators, which maintain a low spatial-phase order when the spatial phase coupling term is zero in $\dot{x}_i$, the chiral swarmalators naturally self-organize into a phase wave formation with an even distribution of phases across the collective. The high value for $S$ when $K > -0.9$ is a direct result of each agent's inherent circular motion and each collective starting with a uniform distribution of phases, meaning the agents' orientations are spread across $0$ and $2\pi$. Even when agents anti-couple ($K < 0$), the inherent circular motion ensures that a single spinning vortex will form. We also note that the trend in inner radius of the collective decreases with $K$, this results from the fact that as $K$ decreases, agents will tend to phase couple more easily with other agents with whom they have a phase difference closer to $\pi$; this will drive the collective to lower the inner radius since this is the point at which agents with a phase difference of $\pi$ will be closest to each other.

The two concentric ring formations shown in Supplementary Fig. 15b happen when $F2$ and $K = 0, J = 1$. Each ring is formed by a natural frequency group and the two are composed of agents revolving in opposite directions. As shown in Supplementary Fig. 15g, the average radius of each frequency group oscillates over time, and this behavior persists even when the collectives run for longer simulation times. The radial oscillation occurs because each natural frequency group has equal and opposite angular velocities that are paired with the phase behavior. As agents minimize their phase difference with agents from the other natural frequency group, which happens twice for each phase cycle, the attraction between these agents maximizes so that a single ring appears. Since the phase difference between agents is continuously increasing or decreasing, the two ring formations quickly separate back into concentric formations. Supplementary Fig. 15g shows that the two groups oscillate between being outside and inside. This most likely occurs because each group has a natural revolution radius of 1 and it is easier for each agent to continue traveling along the direction closest to their instantaneous direction of travel when the attraction between the two groups begins to decrease. This means that agents from the outer ring, which were moving towards the center as their attraction with agents from the other natural frequency group grew, will continue traveling towards the collective centroid once the attraction begins to decrease. It is interesting to note that while the frequency of radial oscillation remains almost constant throughout time, the amplitude changes a good amount; this could be due to slightly non-uniform distributions in the agents' $x$, $y$, and $\theta$ values at the beginning of the simulation. The slightly non-uniform distributions could drive each group to reach variable maximum and minimum radii; however, the average radius remains the same over time and equal to each group's inherent revolution radius.

The dense clusters observed for non-chiral swarmalators are also seen for positive values of $K$ and $J$ with the chiral swaramalators (Supplementary Fig. 15c). The general revolving cluster

behavior, however, persists throughout negative $J$ as well for the chiral swarmalators when $F2$ as shown by the heat map of maximum distance between the centroid of the two natural frequency groups in Supplementary Fig. 15h. The densest clusters are formed at high $K$ and $J$ since this enables agents from the same natural frequency group to synchronize and cluster with similarly phased agents. As $J$ decreases, each natural frequency group cluster becomes sparser and forms an elliptical-like formation that oscillates in attraction and repulsion to the other cluster. When $J < 0$, lower $J$ enables agents within the same natural frequency group to increase their neighbor spacing since they synchronize their phases and repel each other more strongly. The inherent circular motion of the agents, however, induces clustering since they share the same phase within each natural frequency group. Agents from opposite natural frequency groups still undergo oscillatory spatial attraction and repulsion two times per phase cycle (revolution cycle). This induces agents from opposite natural frequency groups to have high attraction and repulsion through each revolution; an elliptical-like formation enables agents from each group to balance the time-varying attraction / repulsion forces that drive each agent to (1) maintain its inherent circular motion, (2) increase its spacing from similarly phased agents, and (3) move towards or away from agents in the opposing natural frequency group (Supplementary Fig. 15d). Supplementary Fig. 15i shows that the major axis of each natural frequency group increases as $J$ becomes lower, which agrees with the statements above.

The image shown for Supplementary Fig. 15e shows the collective with $F1, K = -1, J = 1$ at the end of a simulation; during the simulation the collective starts out in a phase wave formation and over time begins to elongate into a rotating ellipse until the collective forms an ellipse with a very small minor axis (essentially a line) as shown in the image. The behavior repeats itself across different values of $K$ and the ellipse's major axis increases dramatically as $J$ approaches $-1$, as shown in Supplementary Fig. 15j.

When $F4$, half of the collective has an inherent circular motion in the clockwise direction, and the other half the counterclockwise direction; agents have an uniform distribution of revolution radii between 0 and 1. Supplementary Figs. 16a-e show some of the most interesting behaviors when there is a natural frequency spread and their corresponding characterization in Supplementary Figs. 16f-j. The global attraction between agents and their inherent circular motion enables the collective to form a single vortex when $K = 0, J = 1$ (Supplementary Fig. 16a); a small ring of phase-disordered agents on the boundary of the collective keeps the collective from having as high of a value for $S$ as when $F2$. The agents on the boundary each have a very small revolution radius and high natural frequency; this prohibits them from matching the phase of nearby agents and causes them to be repelled to the outer region of the collective. Supplementary Fig. 16f also shows that the degree of synchrony remains low even with positive $K$ because of the spread in natural frequencies across negative and positive values. Supplementary Fig. 16b shows two revolving clusters with a group of phase-disordered agents that remains around the centroid of the collective while the two clusters revolve about it at an average radius of 1. The plot in Supplementary Fig. 16g shows that the collective's and each natural frequency group's degree of synchrony significantly increases at $K \approx 1.5, J = 1$, but remains relatively low because of the natural frequency spread.

Supplementary Fig. 16c results from $F3, K = 0, J = 1$ and shows a very similar vortex to the one shown in Supplementary Fig. 16a. The trends for spatial-phase order and degree of synchrony shown in Supplementary Fig. 16h also closely resemble those in Supplementary Fig. 16f; this demonstrates that even though the whole collective has an inherent circular motion where agents travel in the same direction, the uniform distribution for the natural frequencies keeps the agents from reaching a very high degree of synchrony when $K < 1, J = 1$.

A revolving synchronized cluster with a ring of phase-disordered agents is shown in Supplementary Fig. 16d; the agents forming the outer ring of the collective each have a small revolution radius and a high natural frequency. As shown in Supplementary Fig. 16i, the spatial-

phase order remains above $\sim 0.5$ when $-1 < K < 1$ because the synchronized cluster is traveling in a circular trajectory around the collective's centroid. Even though there is no instantaneous distribution of phases about the centroid, as was the case when the collective formed a vortex, most agents are synchronized and have a position about the centroid that is phase-correlated. The spatial-phase order cannot be higher because of the phase-disordered agents that remain even when $K$ is positive.

When $J < 0$, agents with similar phases repel each other; as shown in Supplementary Fig. 16e, agents with a larger revolution radius and thus a lower natural frequency tend to synchronize more easily and form a large revolving orb. Agents with a low revolution radius and a high natural frequency are unable to synchronize and thus attract to each other to form a cluster of phase-disordered agents closer to the collective's centroid. Supplementary Fig. 16j shows that order remains relatively constant at a low value across negative and positive values of $K$ since the collective either has high phase disorder ($K < 0$) or has partial synchronization ($K > 0$) at which point agents with the same phase are evenly distributed circumferentially about the collective's centroid. In both cases there is low opportunity for the collective to achieve circumferential phase organization. The degree of synchrony, however, begins to increase to a higher value when $K \approx 0.7$; this enables the collective to partially synchronize and encapsulate a tight cluster of phase-disordered agents.

An overview of the chiral swarmalators' emergent behaviors with natural frequency coupling is shown in Supplementary Fig. 17 and the characterization of some of the emergent behaviors is shown in Supplementary Fig. 18. Supplementary Fig. 18a shows the emergent formation when $F2, K = 0, J = 1$; two non-concentric phase waves with counterrotating agents form side by side. The non-concentric formation occurs because of the phase shift in $\dot{\boldsymbol{x}}_i$ and $\dot{\theta}_i$ determined by the agents' natural frequency. The distance between the centroids of the two natural frequency groups is shown by the heat maps in Supplementary Fig. 18b, with a maximum occurring around $0.2 < K < 1$ and $J \approx 1$. The distance between the group centroids is non-zero for almost all positive values of $K$. The frequency coupling enables the current model to produce behaviors very similar to spermatozoa vortex arrays when there is local coupling.

When $F4$, the collective begins to mix more easily because the natural frequency distribution prevents each group from synchronizing with itself and producing the strong oscillatory attraction and repulsion with the opposing group, as was the case when $F2$. The double vortex formation is shown in Supplementary Fig. 18c, where two vortices form next to each other because of the phase shift in $\dot{\boldsymbol{x}}_i$ and $\dot{\theta}_i$. The heat map in Supplementary Fig. 18d shows that the greater distance between the centroids of the two natural frequency groups occurs when $J > 0$ and $K > 0$. This is reasonable since these are the regions of the $K - J$ parameter space in which the clusters are most likely to enable coupling between agents with a similar sign for their natural frequency and thus enable some oscillatory attraction and repulsion with agents from the other natural frequency group.

**Supplementary Note 7. Order parameter.**

This discussion defines the frequency group phase coherence order parameter which is used throughout some supplementary plots.

$$Z_{FG} = \frac{1}{2}\left(\frac{1}{n}\sum_{j}^{n} e^{i\theta_j} + \frac{1}{m}\sum_{k}^{m} e^{i\theta_k}\right) \tag{1}$$

Eq. 1 is used to calculate the average degree of synchrony within the collective when there are multiple natural frequency groups. In Equation (1) there are $n$ agents within a natural frequency group and $m$ agents in another group. The degree of synchrony for the collective is the average of the degree of synchrony within each group. In this study we look at the average degree of synchrony within groups when there are two natural frequency groups.

**Supplementary Note 8. Analysis of Sync State**

**Global Coupling Model with Noise**

The global coupling model can be rewritten with white noise terms $\boldsymbol{\xi}_i$ and $\eta_i$ to study its effect on the emergent collective behaviors.

$$\dot{\mathbf{x}}_i = \mathbf{v}_i + \frac{1}{N}\sum_{j\neq i}^{N}\left[\frac{\mathbf{x}_j - \mathbf{x}_i}{|\mathbf{x}_j - \mathbf{x}_i|}\left(A + J\cos(\theta_j - \theta_i - Q_{\dot{x}})\right) - B\frac{\mathbf{x}_j - \mathbf{x}_i}{|\mathbf{x}_j - \mathbf{x}_i|^2}\right] + \boldsymbol{\xi}_i(t) \tag{2}$$

$$\dot{\theta}_i = \omega_i + \frac{K}{N}\sum_{j\neq i}^{N}\frac{\sin(\theta_j - \theta_i - Q_{\dot{\theta}})}{|\mathbf{x}_j - \mathbf{x}_i|} + \eta_i(t) \tag{3}$$

**Global coupling, identical, non-chiral:** We freeze $(J, K, Q_{\dot{x}}, Q_{\dot{\theta}}, \sigma) = (1, 1, 0, 0, 10)$ and vary $d$ (noise). Supplementary Fig. 40 and Supplementary Movie 8 show how the sync state changes with increasing $d$. At $d = 0$, the swarmalators are frozen in a disk. For $d > 0$, they melt and begin to move around, blurring the disk edge (the radius increases) and degrading the overall phase coherence. We quantify this loss of synchrony by plotting the Kuramoto phase coherence order parameter (Z) versus $d$ (Supplementary Fig. 41, a global measure) and by plotting the two point orientation correlation function (Supplementary Fig. 42, a local measure) defined as

$$C_{n,n}(r) \coloneqq \langle\frac{1}{N(N-1)}\sum_{i,j}\mathbf{n}_i\cdot\mathbf{n}_j\delta(r_{ij} - r)\rangle \tag{4}$$

Where $\mathbf{n}_i = (\cos\left(\theta_i + \frac{\pi}{2}\right), \sin(\theta_i + \frac{\pi}{2}))$ is the 'orientation' of the $i$ -th paticle, $r_{ij}$ is the distance between $i, j$, and $\langle\cdot\rangle$ denotes the ensemble average. $R$ bifurcates from zero at $d \approx 1$. $C_{n,n}(r) >\approx 0.6$ for $d > 0.6$. For larger $d$, it begins to decay, before finally becoming zero as sync is lost. We also computed the (spatial) velocity auto-correlation function,

$$C_{v,v}(t;\tau) \coloneqq \langle\frac{1}{N}\sum_{i}\mathbf{v}_i(t)\,\mathbf{v}_i(t+\tau)\rangle \tag{5}$$

Where $\mathbf{v}_i$ is the unit vector of the spatial velocity. At $d = 0$, the swarmalators relax slowly into a fixed point and so $C_{v,v}(t)$ declines slowly from 1 (Supplementary Fig. 43). For any finite $d$, however, the brownian motion dominates and $C_{v,v}$ has small fluctuations about 0.

**Global coupling, non-identical, non-chiral:** Next we explore how distributed $\omega_i$ affect the above findings. We focus on the case where the natural frequencies are drawn uniformly at random:

$$U(-\Omega, \Omega) \tag{6}$$

$$\omega_i = (-\Omega, -1] \cup (1, \Omega) \text{ for } \Omega = 3 \tag{7}$$

Supplementary Fig. 44 and Supplementary Movie 9 shows how the sync state deform for increasing $\Omega$ and zero noise $d = 0$ ( we freeze $(J, K, \sigma) = (1,1,10)$ as before). For small $\Omega < \Omega_c$, the static sync state survives: swarmalators stay motionless with their phases locked. Note, however, that because they are non-identical, in the locked state the agents do not have identical phases, but rather exhibit a phase gradient. This phase gradient deforms the disk-like spatial density (Supplementary Fig. 44a and b). Beyond a critical $\Omega_c = 1$, however, the static sync bifurcates into an unsteady partial sync state. Here, the disk appears to 'boil': the fast swarmalators near the disk edge break free and vacillate noiselessly around the boundary. The slow swarmalators stay locked in the core, which executes small deformations itself (Supplementary Fig. 43c-f). For $\Omega \gg \Omega_c$, all phase coherence dies out and an incoherent gas like state appears. Supplementary Fig. 45 charts this trend: $Z(\Omega)$ decreases for increasing $\Omega$. Supplementary Fig. 46 shows a log-log plot the auto-correlation of the velocity $C_{v,v}(t)$. For $\omega < 1$, each $\Omega$ decreases to zero at a rate increasing with $\Omega$ (left panel). For $\omega > 1$, however, $C_{v,v}$ is wild and oscillatory, indicating the erratic motion of the 'boiling' in the partial sync state. Finally, Supplementary Fig. 47(a) shows the orientation correlation $C_{n,n}(r)$ (for $d = 0$; the right panel we discuss later). We see $C(r) \sim 1$ for small $\Omega$ consistent with Supplementary Fig. 44a. For larger $\Omega$, we see $C_{n,n}$ varies non-monotonically, picking up the full phase wave in Supplementary Fig. 44c-f. Turning on noise creates an interesting effect: non-monotonicity in $Z$ for $\Omega \sim 1$ (Supplementary Fig. 48a). The 'boiling' of the fast swarmalators in the partial sync state gets drowned out by the noise and the blurred sync state is born which has higher phase coherence. As $d$ is increased, this state blurs until becoming fully incoherent. Supplementary Fig. 48 shows this behavior by plotting $Z(d)$ for different $\Omega$ (the effect is clearer when plotted as $Z(d)$ for different $\Omega$; as opposed to $Z(\Omega)$ for different $d$). The $\Omega = 1$ curve, the regime in which the partial sync state exists, exhibits the non-monontonicty. The other $\Omega$ curves are shown for the sake of completion. The $C_{v,v}(t)$ for $d > 0$ are uninteresting; a rapid decline to browinian motion is observed for $d > 0.1$ (not plotted). Similarly the $C_{n,n}(r)$ becomes more blurry until the non-monotonicty disappears and the async state is born (Supplementary Fig. 47b).

**Local coupling, non-chiral:** Two things happen when coupling changes from global to local. For σ the single cluster (Supplementary Fig. 49a) splits into multiple clusters surrounded by a gas of spatially free, but synchronized swarmalators (Supplementary Fig. 49b-e). Then for $\sigma \lesseqgtr 1$, all clusters disappear leaving a mass of sync'd oscillators (Supplementary Fig. 49f). To explore the length scales of the clusters, as well as the inter-cluster separation, we calculate

$$g(r) \coloneqq \frac{1}{N(N-1)} \sum_{i,j} \delta(r - r_{ij}) \tag{8}$$

for different $\sigma$. Supplementary Fig. 52 shows the results for $N = 500$ swarmalators placed uniformly at random in a box of length $L = 4$. For large $\sigma$, there is one cluster and $g$ has only one peak. For smaller $\sigma$ multiple clusters form and $g$ exhibits multiple peaks. Notice, however, that the location of the first peak $r^*$ is the same for each $\sigma$; the radius of each cluster does not depend on $\sigma$. For small amounts of disorder, both with quenched $\Omega > 0$ and active $d > 0$, multiple clusters persist, but eventually disappear for large $d, \Omega$. This is evident by the disappearance of the multiple peaks in Supplementary Fig. 51.

Supplementary Fig. 52 demonstrates the inter-cluster phase correlation by plotting $C_{n,n}(r)$ (bottom row) for $(d, \Omega) = (0.1, 0.2)$. Scatter plots of the corresponding states are shown in the top row for convenience. The first column shows the simple single cluster behavior: $C_{n,n}$ decreases slightly over the cluster and then drops to zero. There is a small fluctuation for large $r$, corresponding to stray swarmalators which have not been absorbed. The second column shows

the multiple-cluster regime where $C_{n,n}$ varies non-monotonically. The first interval of decrease captures the intra-cluster correlation, the second the inter-cluster correlation, and the third the cluster-gas correlation. Finally the third column shows the gas regime where $C_{n,n}$ has small local correlations which disappear quickly as $r$ increases. Supplementary Fig. 53 shows the three $C$ for larger $\Omega$ in which the phase gradient within clusters is wider, which manifests as an oscillation in $C$ from positive to negative values.

**Supplementary Note 9. Analysis of phase waves / vortices**

**Global coupling:** Next we study the vortex like active phase wave. We set $(J, K) = (1, 0, 5)$ throughout this section. Supplementary Fig. 55 shows a scatter plot of the state for increasing amounts of disorder $d, \Omega > 0$ (see Supplementary Movie 12 for evolution of states). The state gradually blurs until becoming incoherent. Supplementary Fig. 55 quantifies this loss of coherence by plotting $S = \max(S_+, S_-)$, which is more than 0 in this state, against $d$ (left panel) and $\Omega$ (right panel).

**Local coupling:** When the coupling becomes local, we see a transition from a single vortex to multiple vortices to a incoherent gas (Supplementary Movie 12). Supplementary Fig. 56 plots the collective states (top row), the correlation functions (middle row), and the radial density (bottom row) for $\sigma = 10, 3, 1$ for small amounts of disorder $(d, \Omega) = (0.1, 0.2)$. For large coupling (left column): notice $C_{\theta,\theta}$ decreases from 1 to $\approx -0.6$ over the length scale of the single vortex, consistent with the phase gradient. The radial density g has a single peak, since there is only one cluster. For intermediary length scales $\sigma = 3$ (middle column), $C_{\theta,\theta}$ is non-zero over the length of a single vortex; there is no inter-vortex correlation. $g$ on the other hand has multiple peaks. Finally, for short range coupling $\sigma = 1$ phase-phase correlation is sparse and a single broad mode in $g$, consistent with the picture of an incoherent gas.

## Supplementary Note 10: Analysis of chirality

We analyze chirality and draw the swarmalators' natural frequencies from the $F2$ distribution. This splits the population into two (half rotating clockwise ($\omega_i < 0$), the other half revolving counterclockwise ($\omega_i > 0$). As defined in the main text, this distribution is to ensure that the radii of revolution are bounded; recall we set the speed to be magnitude unity $\mathbf{v}_i = c_i \mathbf{n}_i = 1\mathbf{n}_i = \mathbf{n}_i$. This means the radius of revolution (when uncoupled) of each swarmalators is $R_i = \frac{|\mathbf{v}_i|}{\omega_i} = \frac{1}{\omega_i}$, so if we allow small $\omega_i$ then we get large $R_i$. The main effect of this distribution is that the phase wave / vortex state splits into two, so that the winding number of the overall state is $k = 2$ (Supplementary Fig. 57). The sync state bifurcates into the bouncing cluster state (see main text; not plotted here). To capture the phase order, we use the order parameter $S_{+,2} = |\frac{1}{N}\sum_j e^{2i(x\pm\theta)}|$. Supplementary Fig. 58 plots $S_2 := \max(S_{+,2}, S_{-,2})$ along with $S$ for increasing $d$ and $\Omega$ (left panel) and for increasing $\Omega$ and fixed $d$ (right panel) which shows a steady decline to incoherence. The velocity-velocity correlation functions were uninformative (similar to Supplementary Fig. 43) so we do not display them here. Supplementary Fig. 59 shows local coupling produces no new new effects. The same the single vortex to multi-vortex to gas-like bifurcations are observed for increasing local coupling; i.e. Supplementary Fig. 59 for chiral swarmalators is qualitatively the same as Supplementary Fig. 56 for non-chiral swarmalators. The only difference is the phase-phase correlation function $C_{\theta,\theta}$ has two peaks, oscillating from positive to negative back to positive, reflecting the phase wave with winding number $k = 2$.

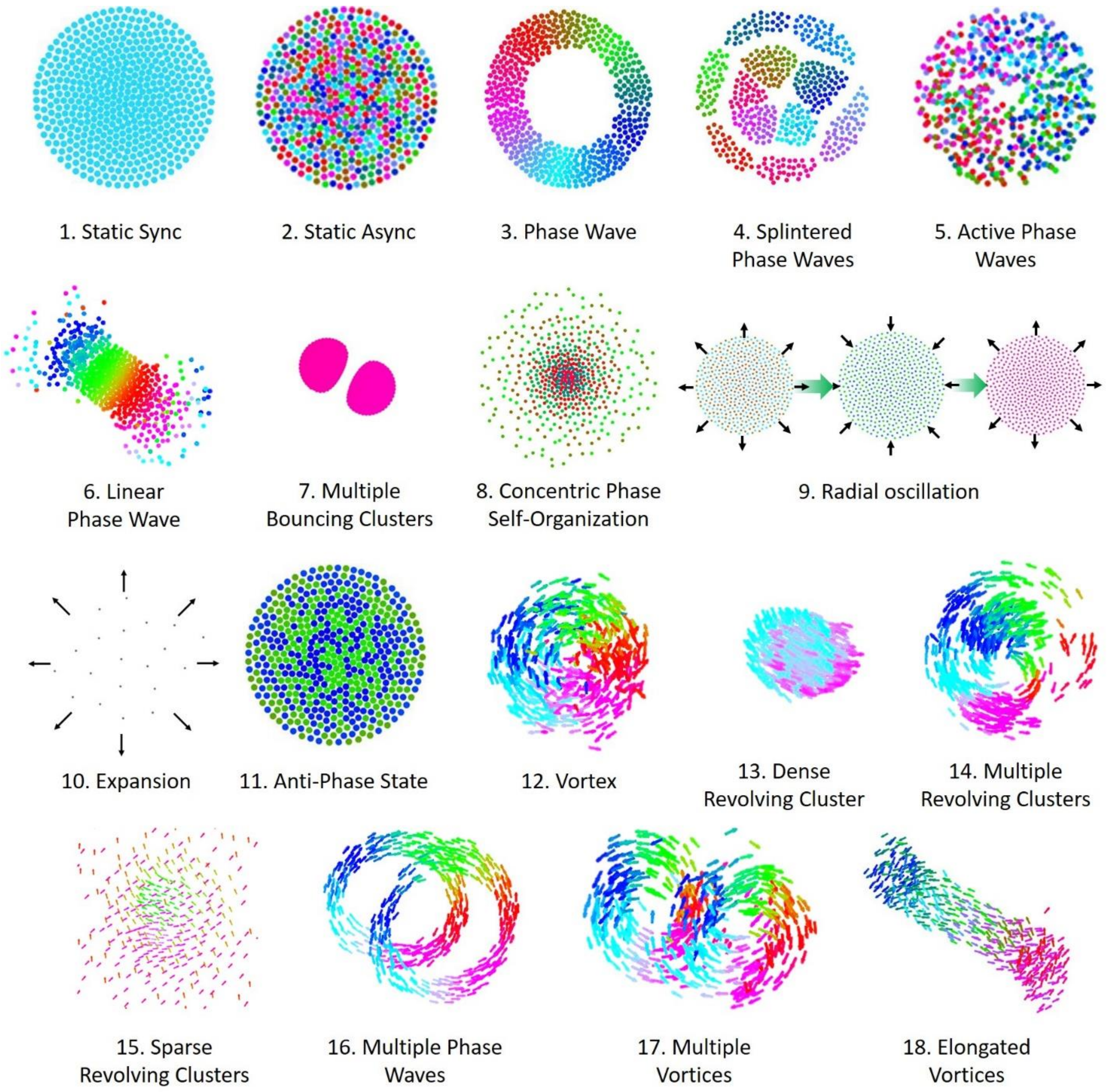


**Supplementary Fig. 1. Summary of emergent states.** The behaviors shown above summarize the major behaviors found through our model and their numbers correspond to their number in Table 1.

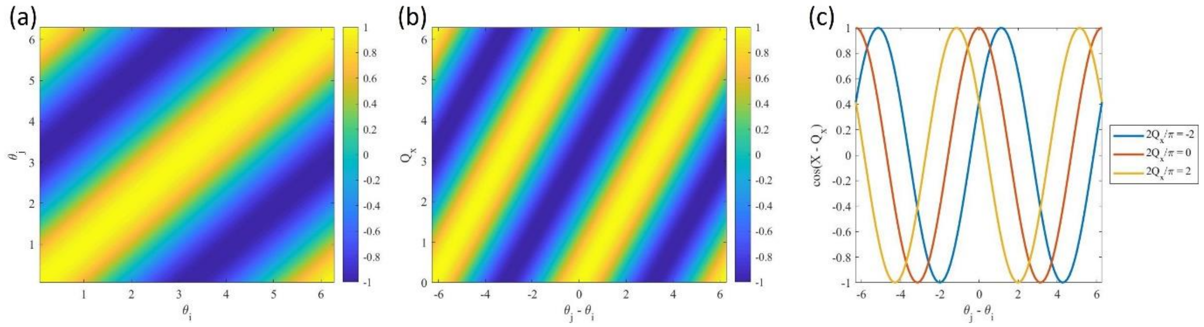


**Supplementary Fig. 2. Phase-interaction parameter space.** Value given by the spatial phase interaction term in Eq. 1: **(a)** Cases explored for $Q_{\dot{x}} = 0$ and different phase values. The color bar is defined by $\cos(\theta_j - \theta_i)$. **(b)** Cases explored for a constant phase shift value $Q_{\dot{x}}$ and different phase differences ($\theta_j - \theta_i$). The color bar is defined by $\cos(\theta_j - \theta_i - Q_{\dot{x}})$. **(c)** Oscillation behavior of the phase coupling-induced motion term in $\dot{\boldsymbol{x}}_i$.

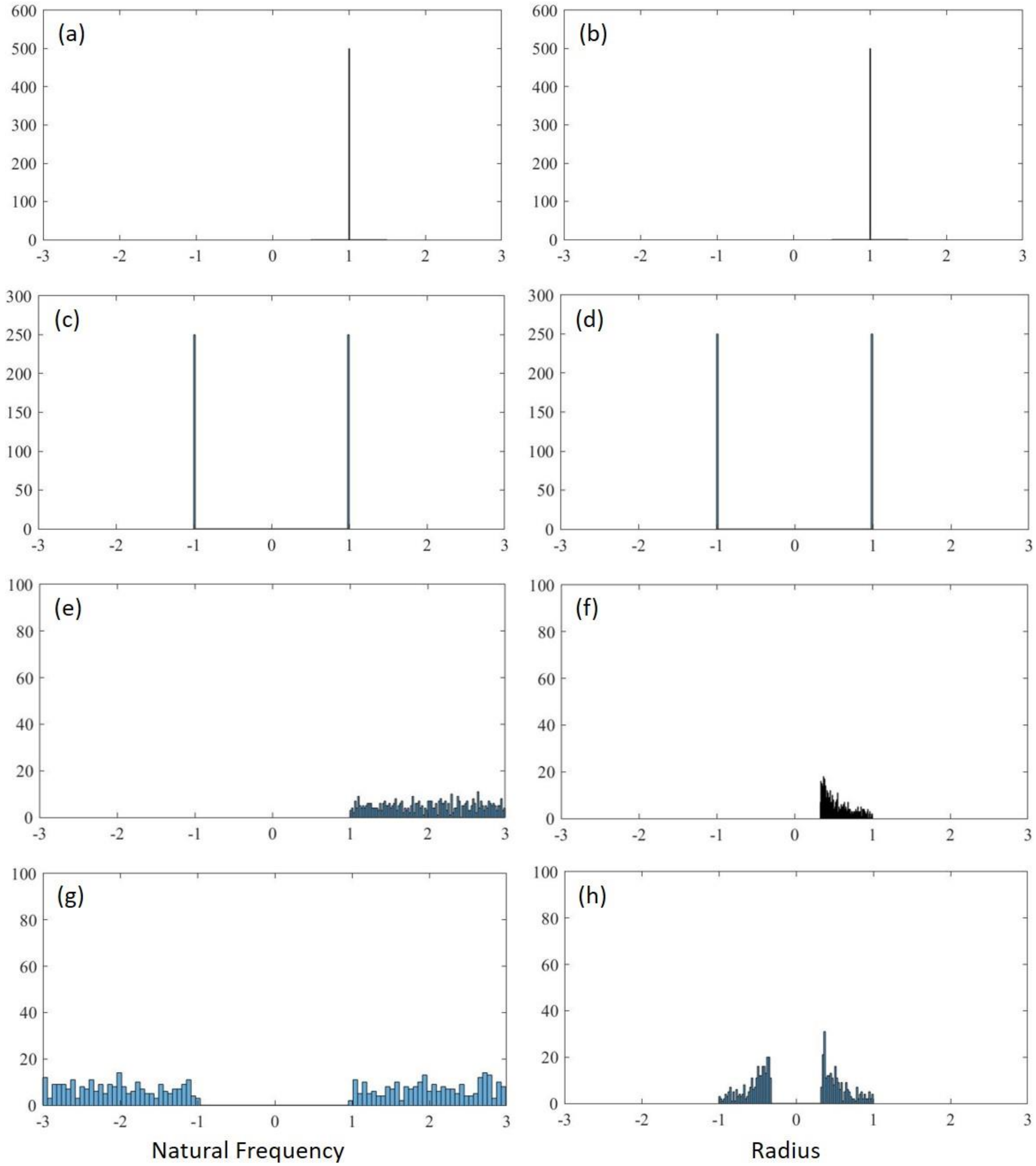


**Supplementary Fig. 3. Natural frequency and revolution radius distributions.** Histogram for each of the natural frequency distributions used throughout the numerical studies. **(a-b)** $F1$; **(c-d)** $F2$; **(e-f)** $F3$; **(g-h)** $F4$. The distributions shown in (a,c,e,g) correspond to the natural frequencies and the distributions shown in (b,d,f,h) correspond to revolution radius.

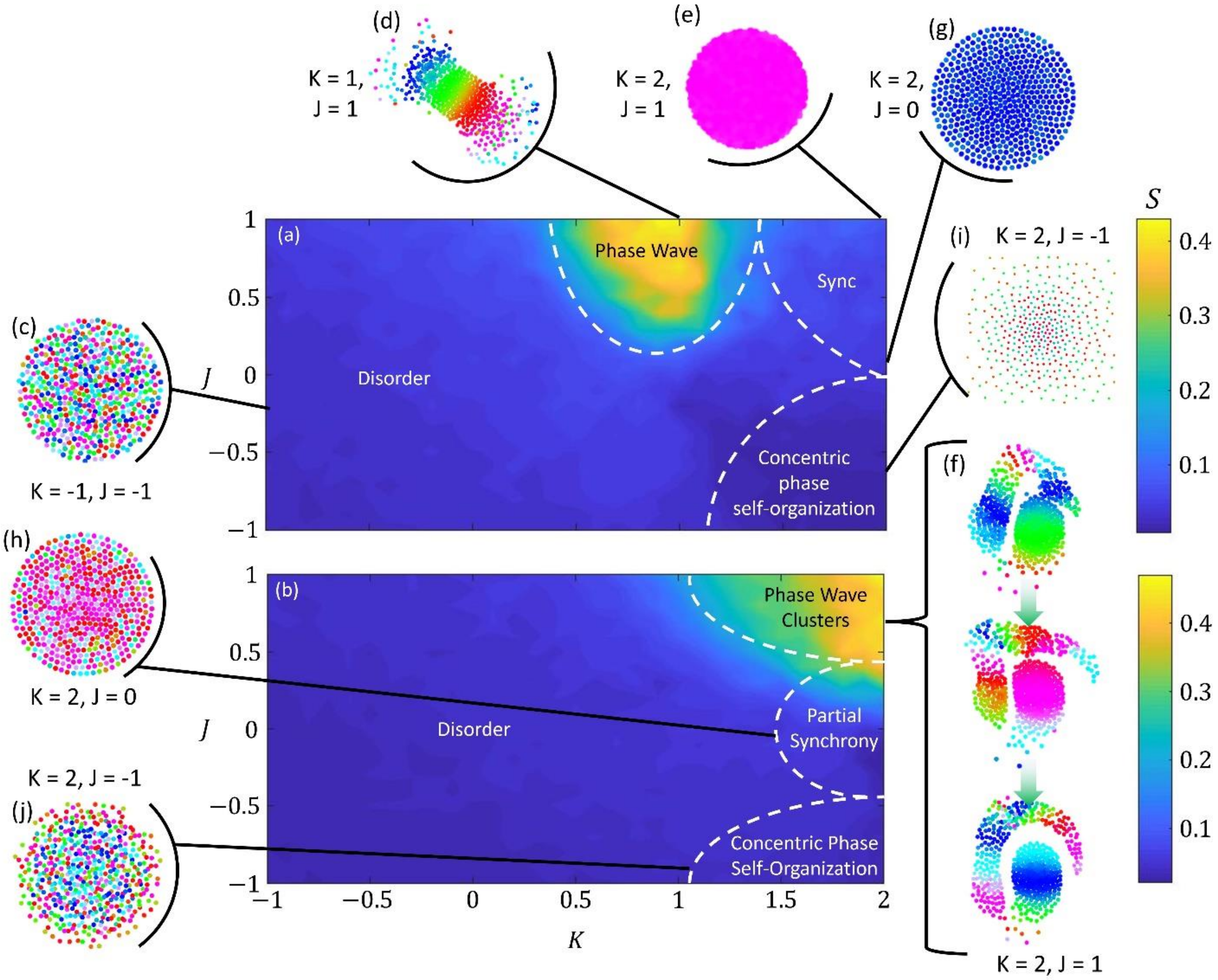


**Supplementary Fig. 4. Non-chiral swarmalators with a natural frequency spread.** Heat maps of $S$ across $K - J$ parameter space is shown for test cases with **(a)** $F3$ and **(b)** $F4$. **(c)** Static async. **(d)** Partial sync with phase wave along an axis. **(e)** Partial sync with synchronized cluster forms at the center. **(f)** Periodic bouncing of partial sync clusters. **(g-h)** Partial sync within a circular formation. **(i-j)** Concentric phase self-organization.

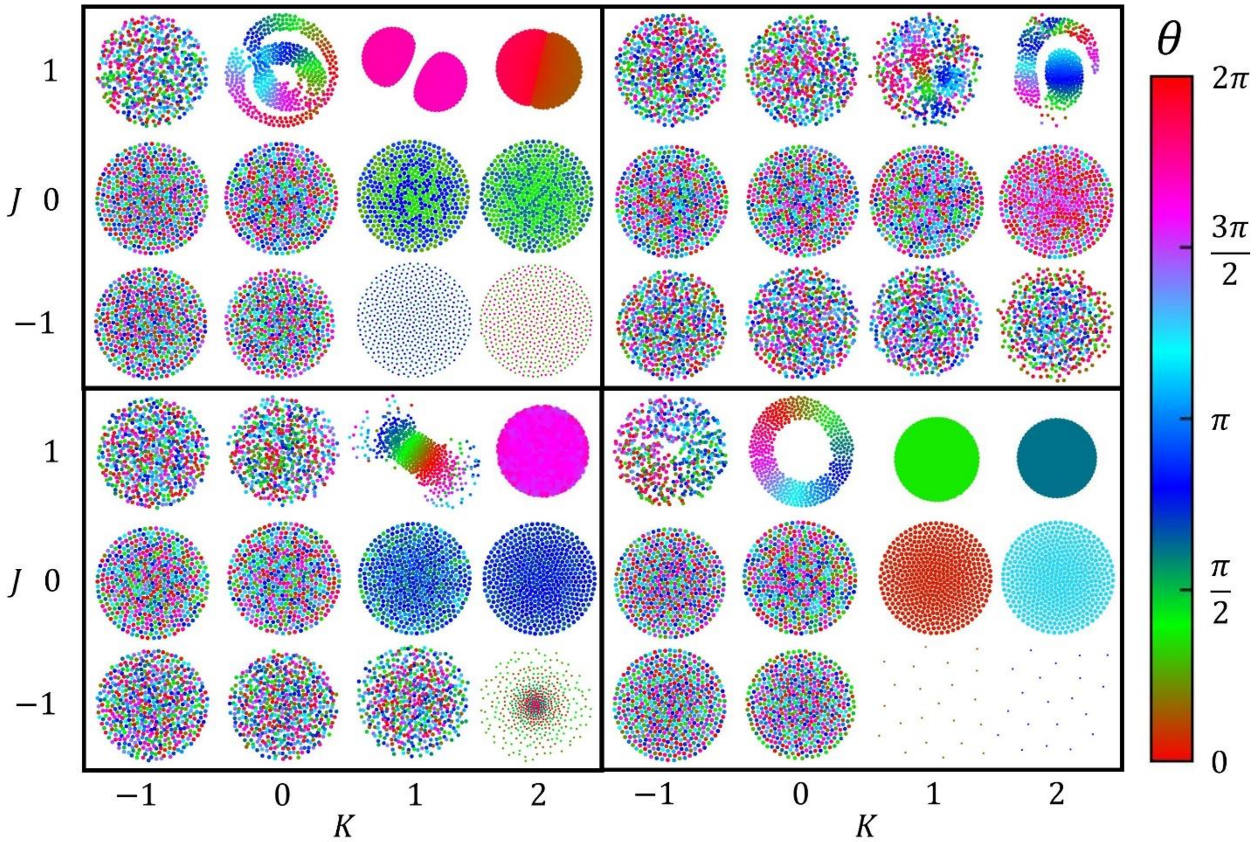


**Supplementary Fig. 5. Collective behaviors of non-chiral swarmalators with no frequency coupling.** $F2$ (Upper left). $F4$ (Upper right). $F3$ (Lower left). $F1$ (Lower right).

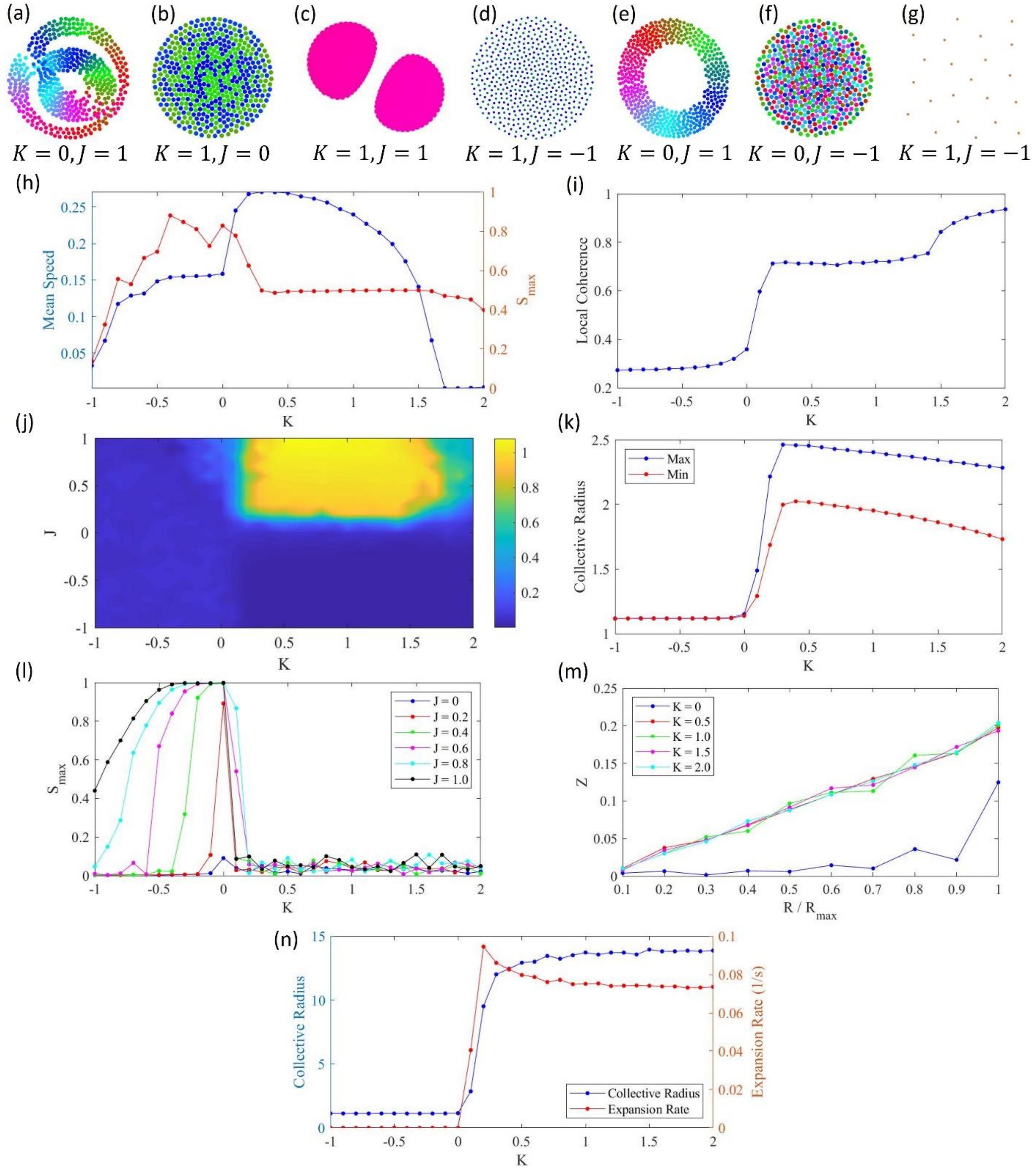


**Supplementary Fig. 6. Characterization of collective behaviors in non-chiral swarmalators with discrete natural frequencies and no frequency coupling. (a-g)** Various emergent configurations of non-chiral swarmalators. **(h-k)** Collective behavior characterizations for the same $\omega$ distributions listed in **(a-k)**, respectively. **(a)** $F2$; **(b)** $F2$; **(c)** $F2$; **(d)** $F2$; **(e)** $F1$; **(f)** $F1$; **(g)** $F1$. **(h)** Mean speed and $S$ order when $J = 1$; **(i)** Max distance between the two natural frequency group centroids; **(j)** Local coherence with neighboring agents when $J = 0$; **(k)** collective radius at peak expansion and contraction when $J = -1$; **(l)** $S$ order for various values of $J$ and $K$; **(m)** phase coherence as a function of distance from the collective's centroid when $J = -1$; **(n)** collective radius and expansion rate when $J = -1$.

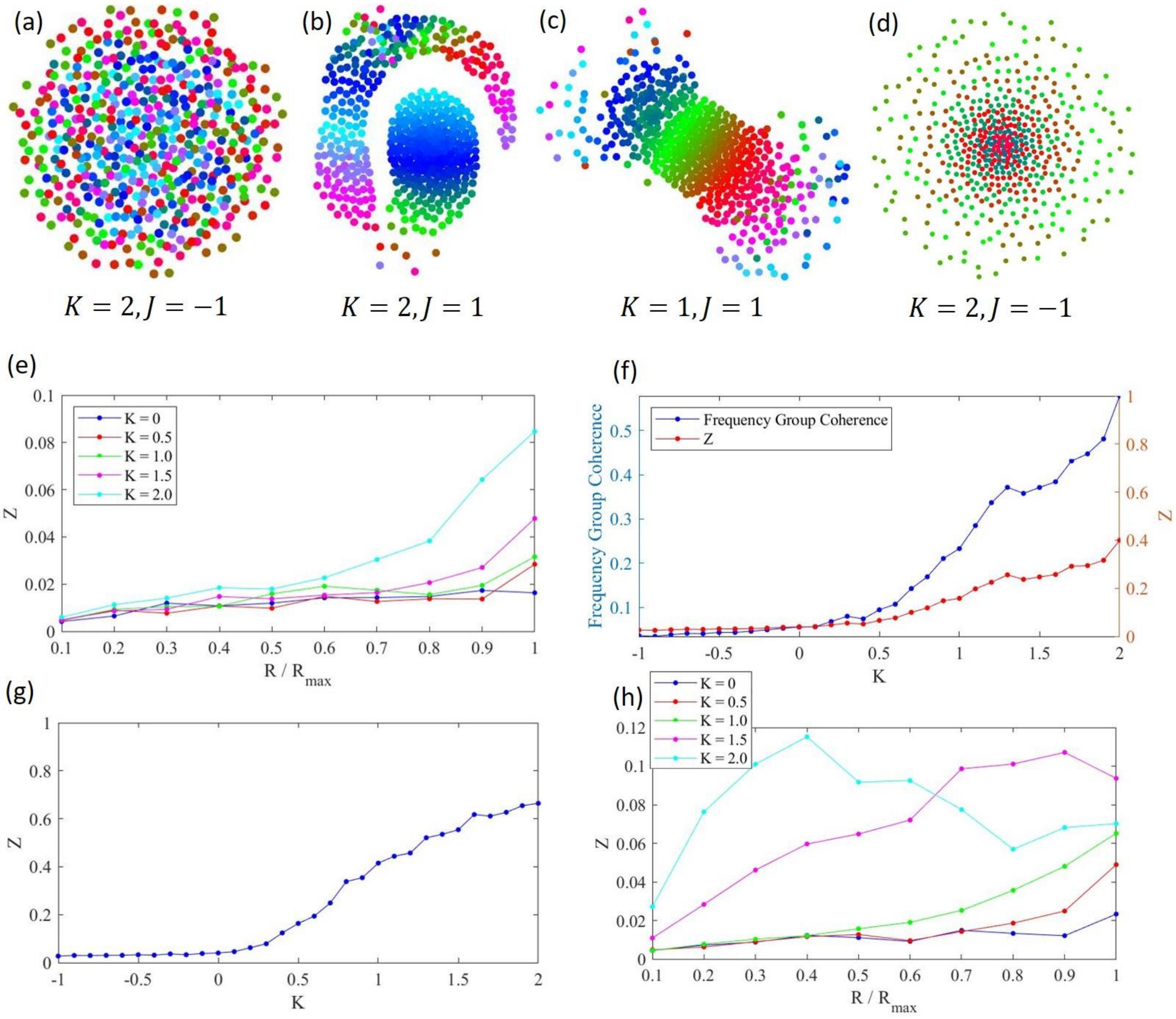


**Supplementary Fig. 7. Characterization of collective behaviors in non-chiral swarmalators with a natural frequency spread and no frequency coupling. (a-d)** Various emergent configurations of non-chiral swarmalators. **(e-h)** Collective behavior characterizations for the same natural frequency distributions listed in **(a-d)**, respectively. **(a)** $F4$; **(b)** $F4$; **(c)** $F3$; **(d)** $F3$. **(e)** phase coherence as function of distance from the collective centroid when $J = -1$; **(f)** Natural frequency group and global phase coherence when $J = 1$; **(g)** phase coherence when $J = 1$; **(h)** phase coherence when $J = -1$.

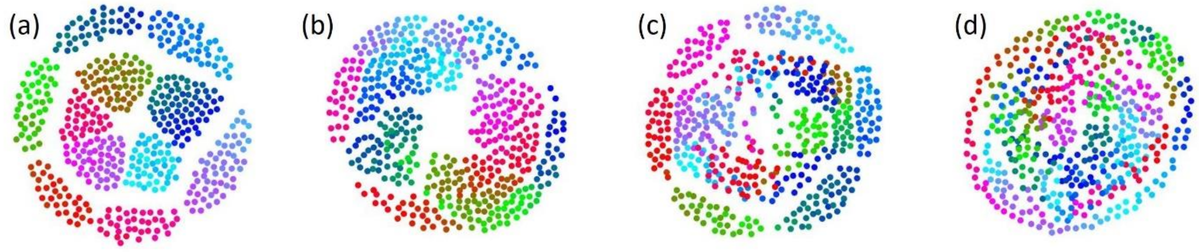


**Supplementary Fig. 8. Splintered phase waves.** Images of collectives with different numbers natural frequency groups when $c, Q_{\dot{x}}, Q_{\dot{\theta}} = 0, K = -0.1, J = 1$. **(a)** $\omega_i \in [-1], [1]$. **(b)** $\omega_i \in [-1], [0], [1]$. **(c)** $\omega_i \in [-1], [-0.5], [0.5], [1]$. **(d)** $\omega_i \in [-1], [-0.5], [0], [0.5], [1]$.

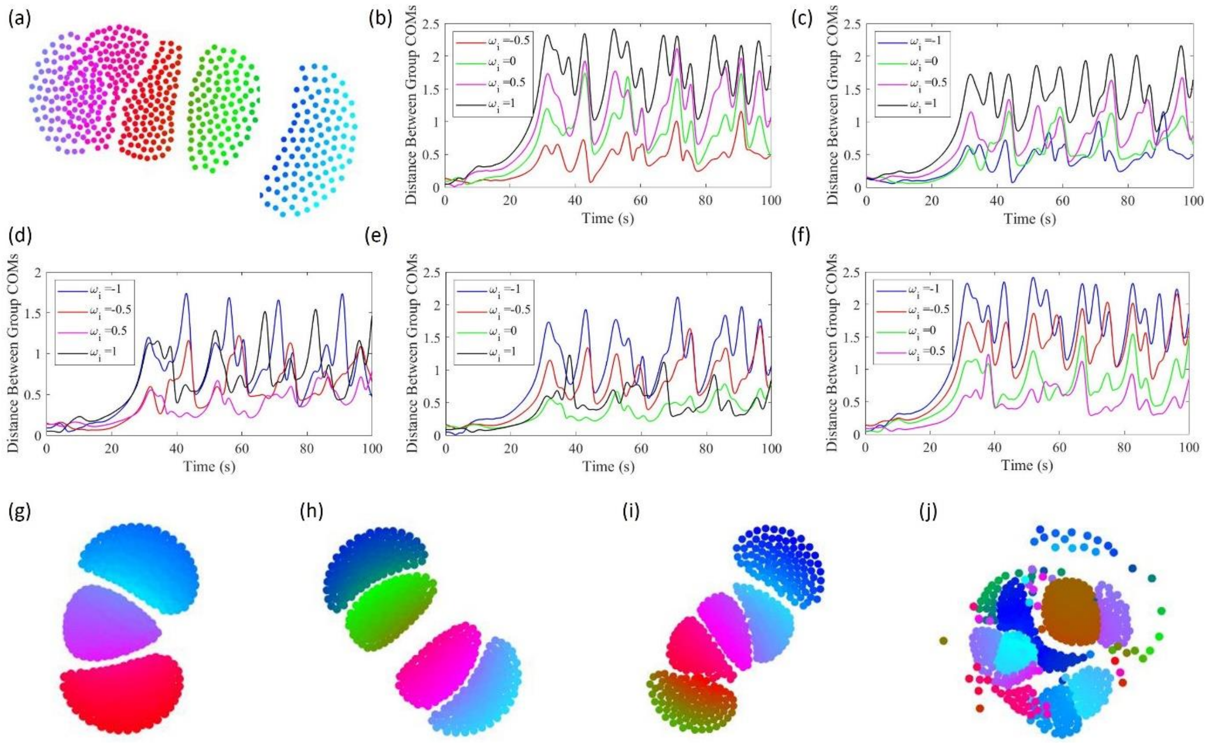


**Supplementary Fig. 9. Frequency group separation. (a)** A collective with five natural frequency groups ($\omega_i \in [-1], [-0.5], [0], [0.5], [1]$) when $c = 0$, and $K, J = 1$. **(b-f)** The distance between each natural frequency group's center of mass and all other frequency groups' center of masses over time. Distance from the center of mass of the group of agents with a natural frequency of **(b)** $\omega_i = -1$, **(c)** $\omega_i = -0.5$, **(d)** $\omega_i = 0$, **(e)** $\omega_i = 0.5$, **(f)** $\omega_i = 1$. **(g-j)** Collectives with differing numbers of natural frequency groups shown after 775 time steps. **(g)** $\omega_i \in [-1], [0], [1]$. **(h)** $\omega_i \in [-1], [-0.5], [0.5], [1]$. **(i)** $\omega_i \in [-1], [-0.5], [0], [0.5], [1]$. **(j)** $\omega_i \in [-4], [-3], [-2], [-1], [0], [1], [2], [3], [4]$.

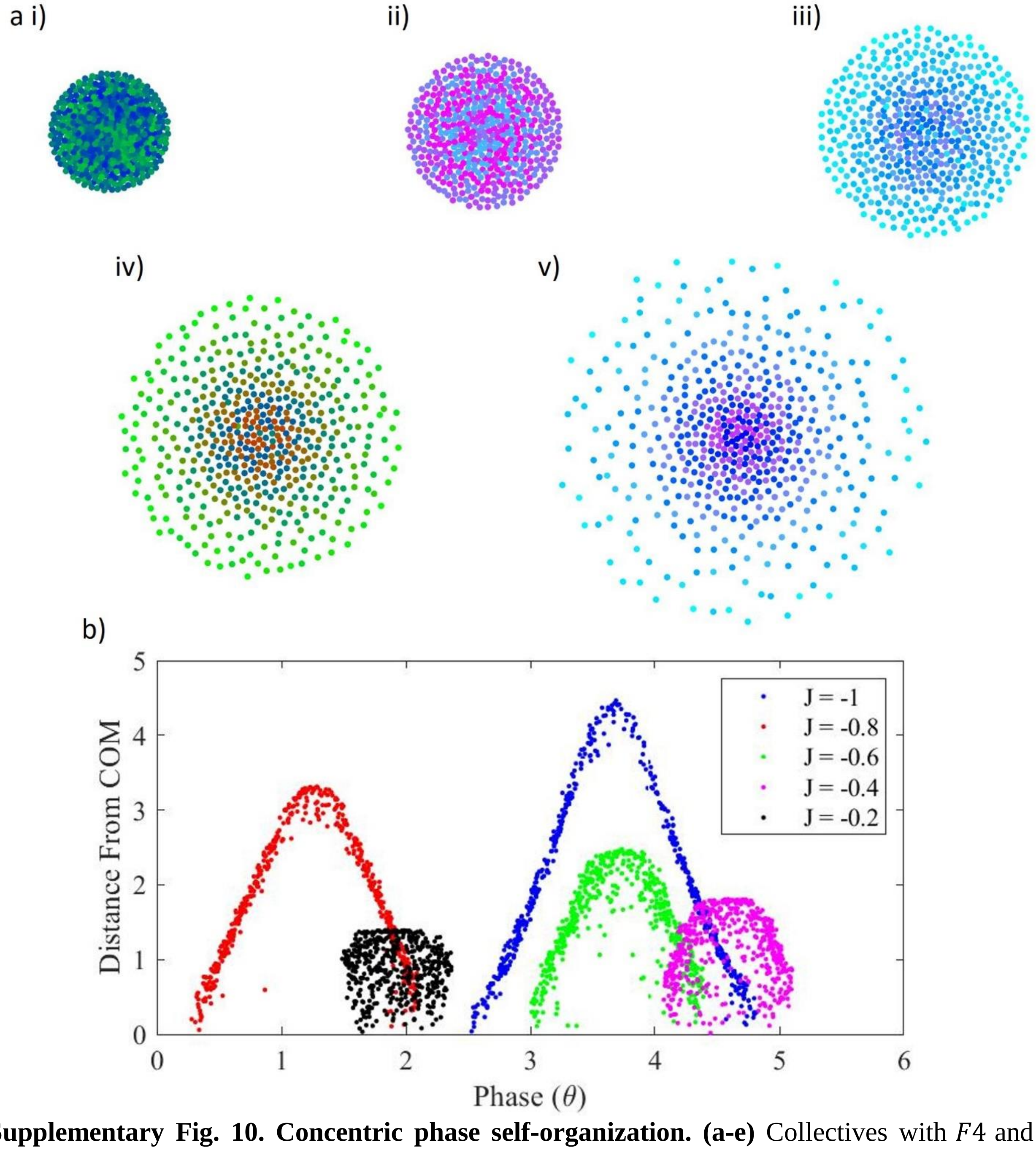


**Supplementary Fig. 10. Concentric phase self-organization. (a-e)** Collectives with $F4$ and different negative values of $J$ that self-organize concentric rings of agents with similar phases. **(a)** $J = -0.2$, **(b)** $J = -0.4$, **(c)** $J = -0.6$, **(d)** $J = -0.8$, **(e)** $J = -1.0$. **(f)** Plot of agents' distance from collective centroid as a function of phase demonstrates that after a certain distance there is distance-based phase self-organization.

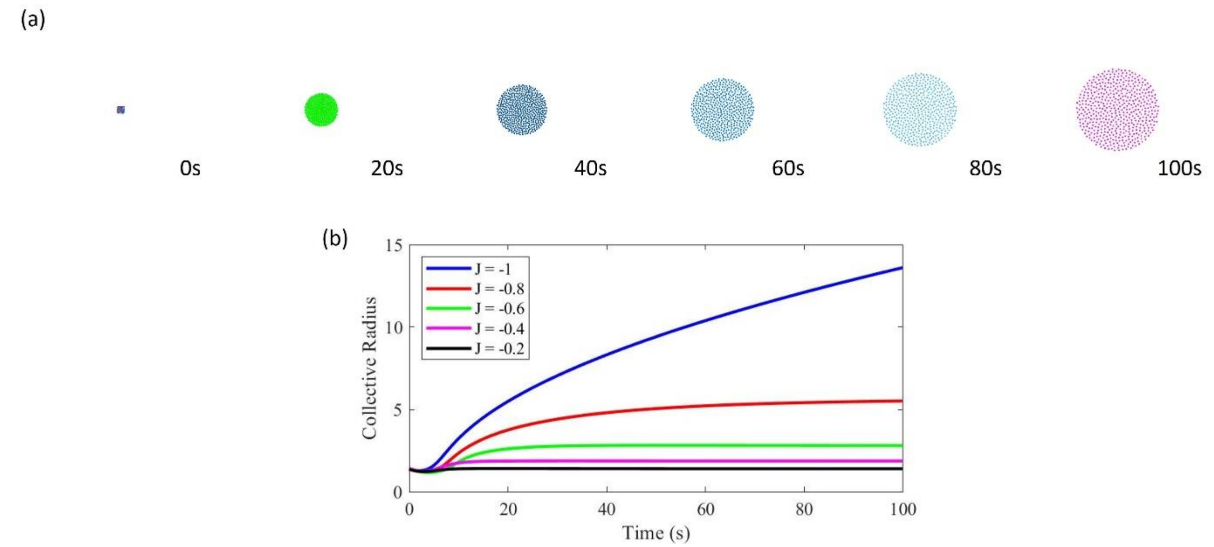


**Supplementary Fig. 11. Collective expansion. (a)** Collective expanding indefinitely when $c = 0, F1, K = 1$, and $J = -1$. **(b)** Collective radius plotted as a function of time for various values of $J$.

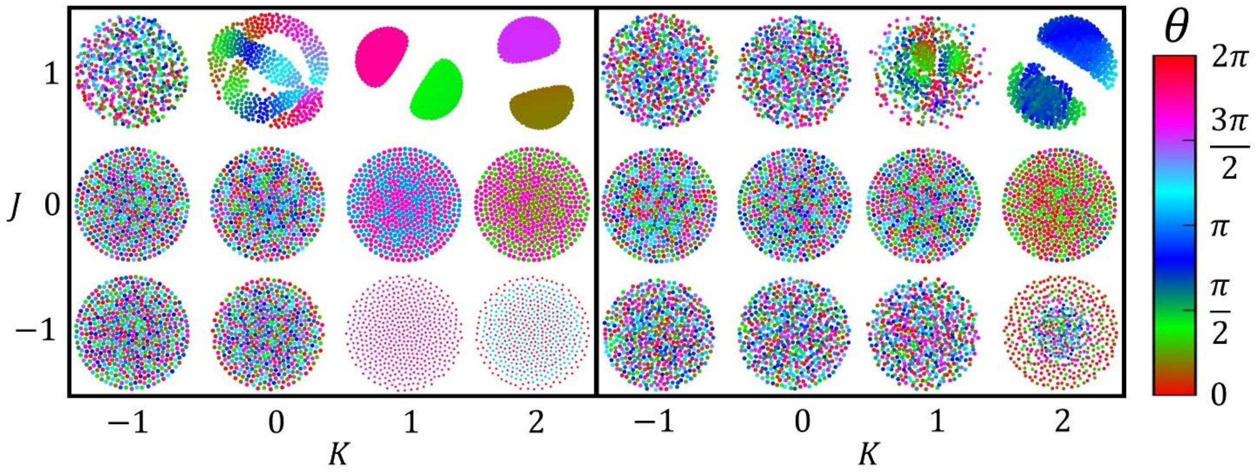


**Supplementary Fig. 12. Collective behaviors of non-chiral swarmalators with frequency coupling.** $Q_{\dot{x}} = \frac{\pi}{2}\left|\frac{\omega_j}{|\omega_j|} - \frac{\omega_i}{|\omega_i|}\right|, Q_{\dot{\theta}} = \frac{\pi}{4}\left|\frac{\omega_j}{|\omega_j|} - \frac{\omega_i}{|\omega_i|}\right|. F2$ (Left). $F4$ (Right).

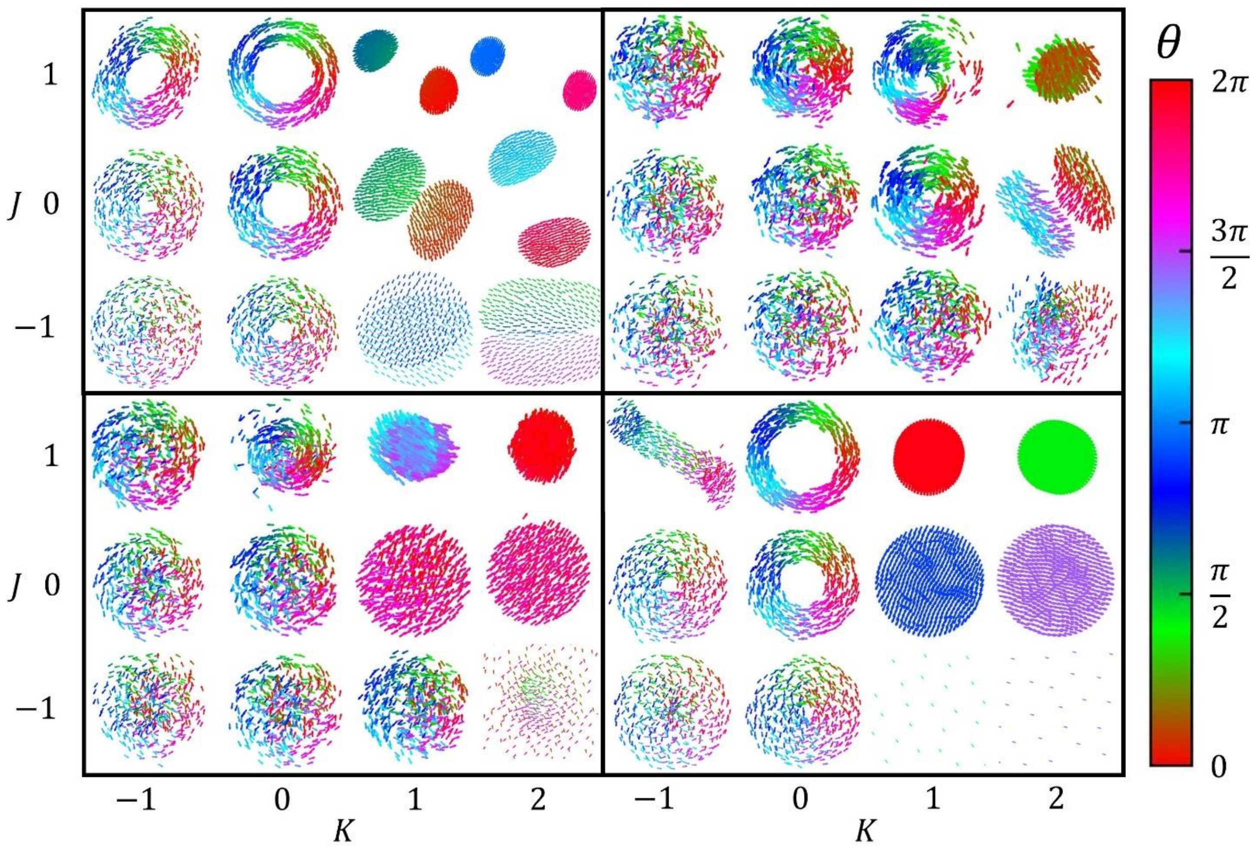


**Supplementary Fig. 13. Collective behaviors of chiral swarmalators with no frequency coupling.** $F2$ (Upper left). $F4$ (Upper right). $F3$ (Lower left). $F1$ (Lower right). Quiver plots are shown here instead of instantaneous positions because chiral swarmalators have an inherent motion driving them.

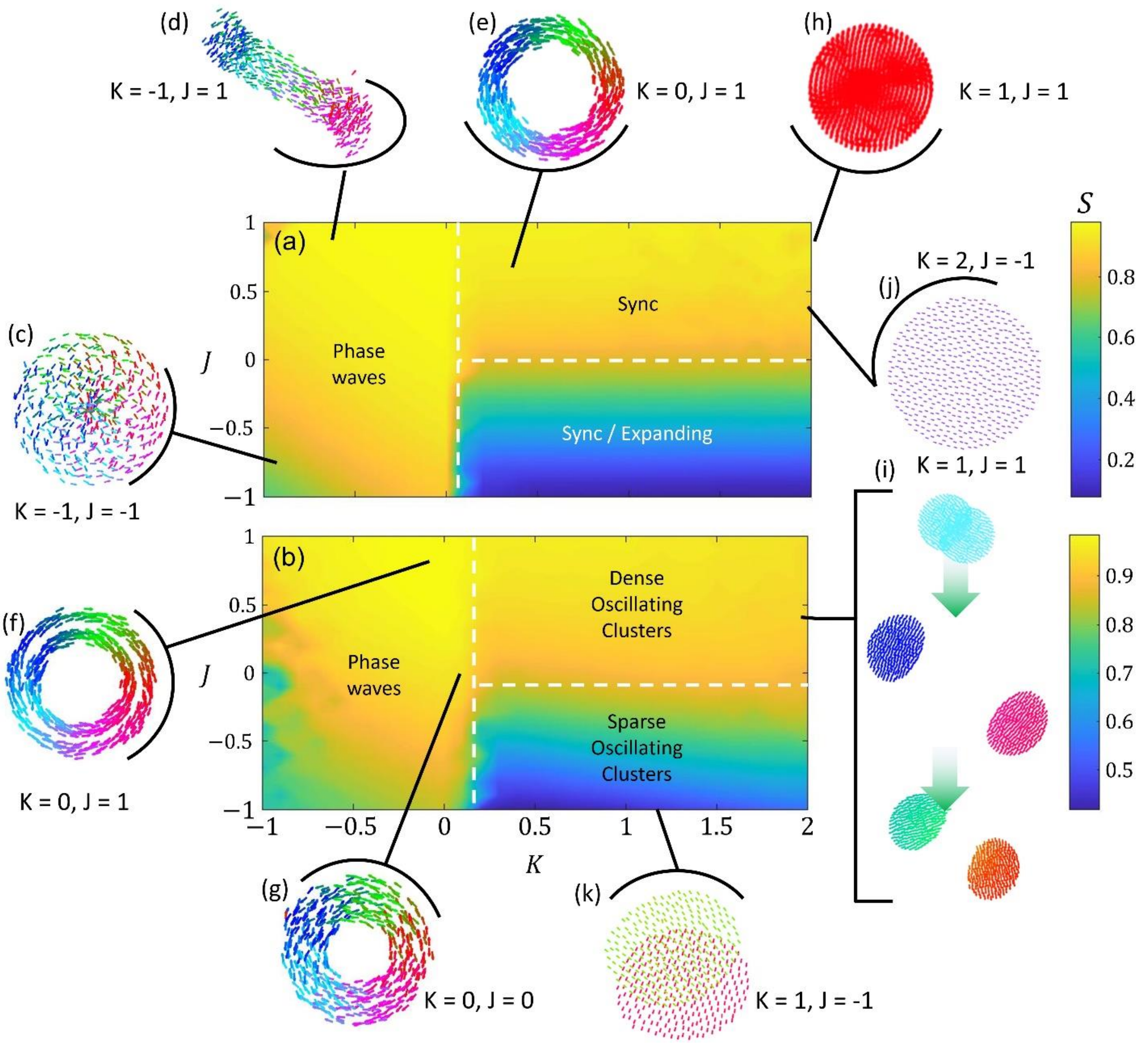


**Supplementary Fig. 14. Revolving swarmalators with no natural frequency spread.** Heat maps of $S$ across $K - J$ parameter space is shown for test cases with (**a**) $F1$ and **(b)** $F2$. **(c)** Phase wave. **(d)** Phase wave along a slim ellipse. **(e)** Phase wave. **(f)** Concentric double phase waves. **(g)** Phase wave. **(h)** Synchronized cluster. **(i)** Dense revolving clusters. **(j)** Expanding synchronized revolving cluster. **(k)** Sparse oscillating clusters.

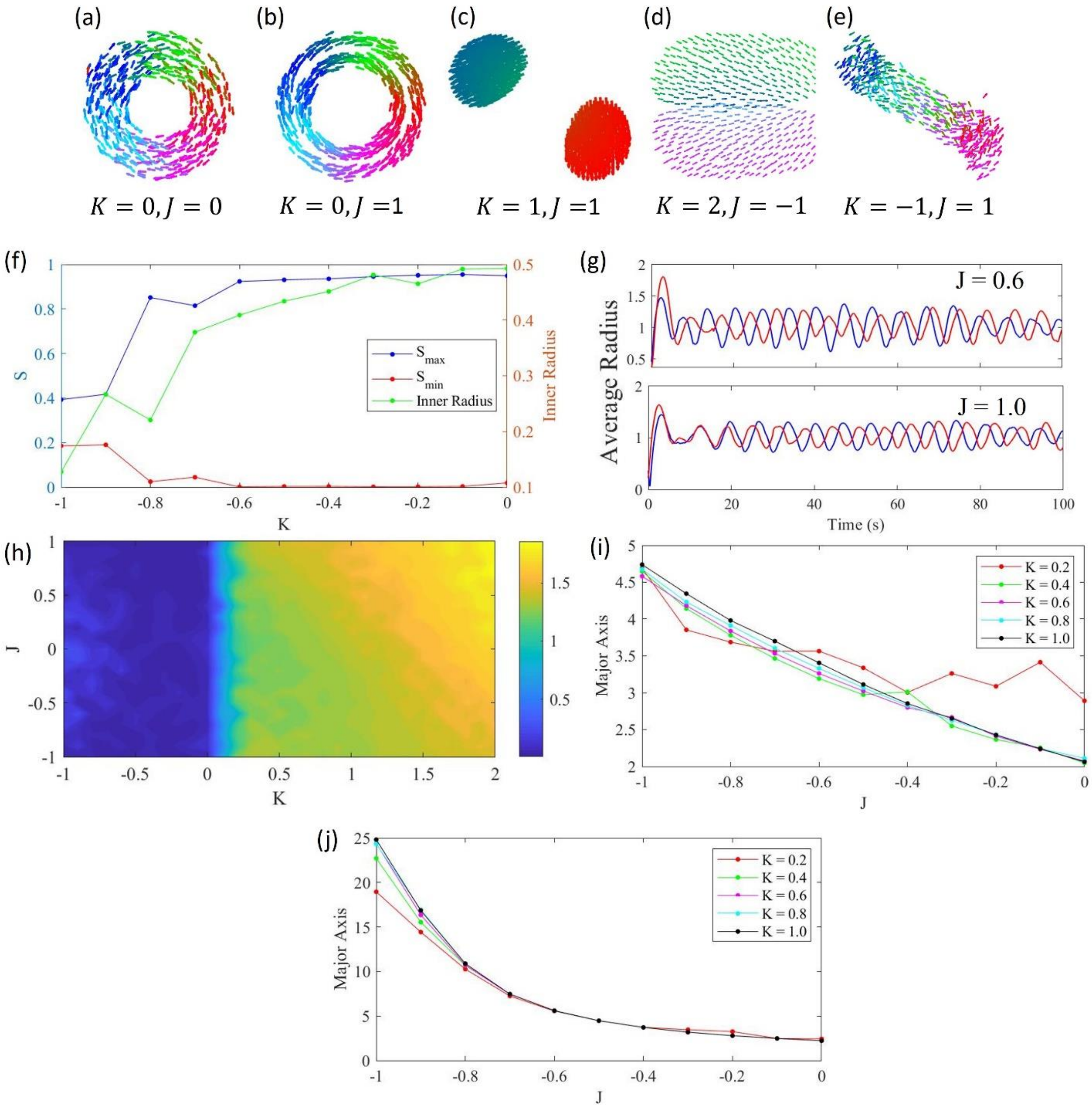


**Supplementary Fig. 15. Characterization of collective behaviors in chiral swarmalators with discrete natural frequencies and no frequency coupling. (a-e)** Various emergent configurations of chiral swarmalators. **(f-j)** Collective behavior characterizations for the same ω distributions listed in **(a-e)**, respectively. **(a)** $F2$; **(b)** $F2$; **(c)** $F2$; **(d)** $F2$; **(e)** $F1$; **(f)** $S_{max}$, $S_{min}$, and inner ring radius when $J = 0$; **(g)** average radius of each natural frequency group over time when $J = 0.6$, 1; **(h)** max distance between the two clusters; **(i)** major axis length for different $K$; **(j)** major axis length when $K = -1$.

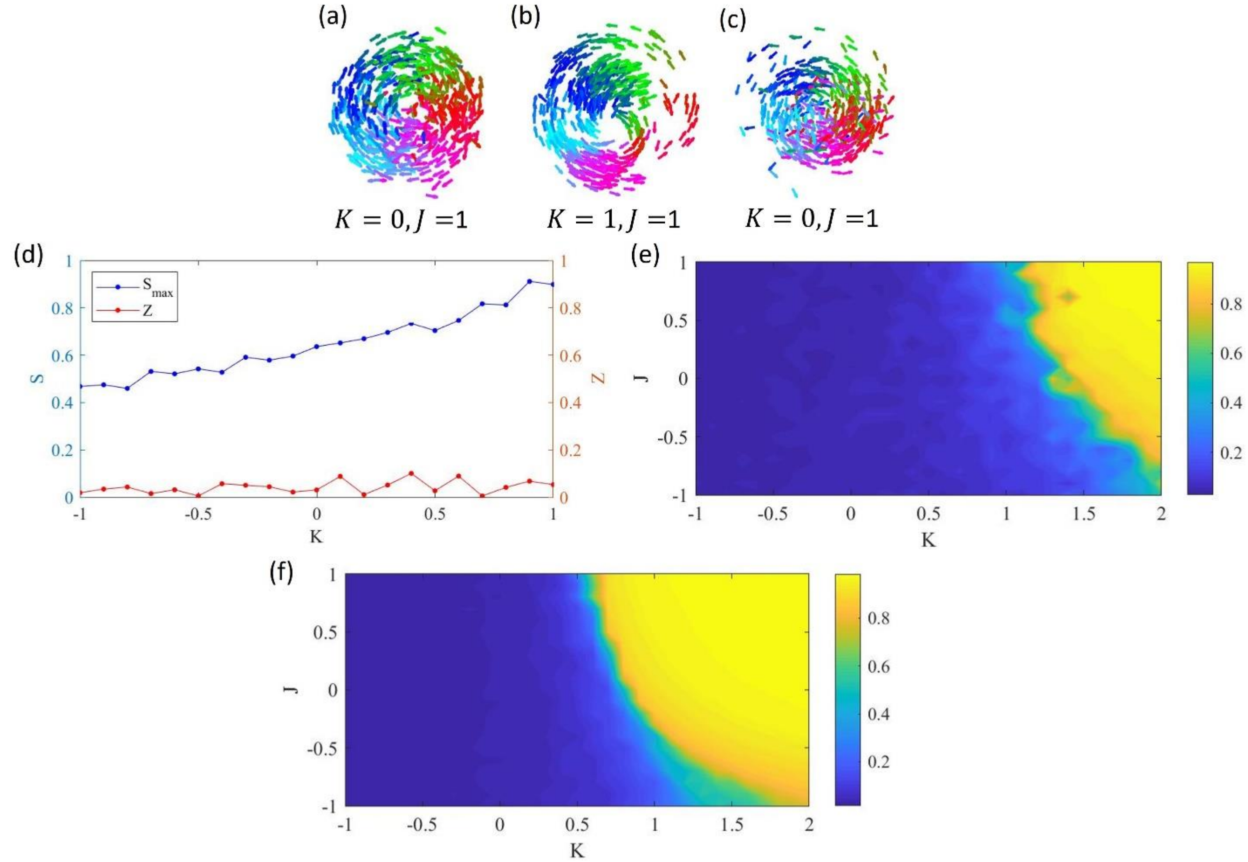


**Supplementary Fig. 16. Characterization of collective behaviors in chiral swarmalators with a natural frequency spread and no frequency coupling. (a-e)** Emergent configurations of chiral swarmalators. **(f-j)** Collective behavior characterizations for the same ω distributions listed in **(a-e)**, respectively. **(a)** $F4$; **(b)** $F4$; **(c)** $F3$; **(d)** $S$ order and phase coherence when $J = 0$; **(e)** Heat map of the average natural frequency group S order. **(f)** Heat map of phase coherence.

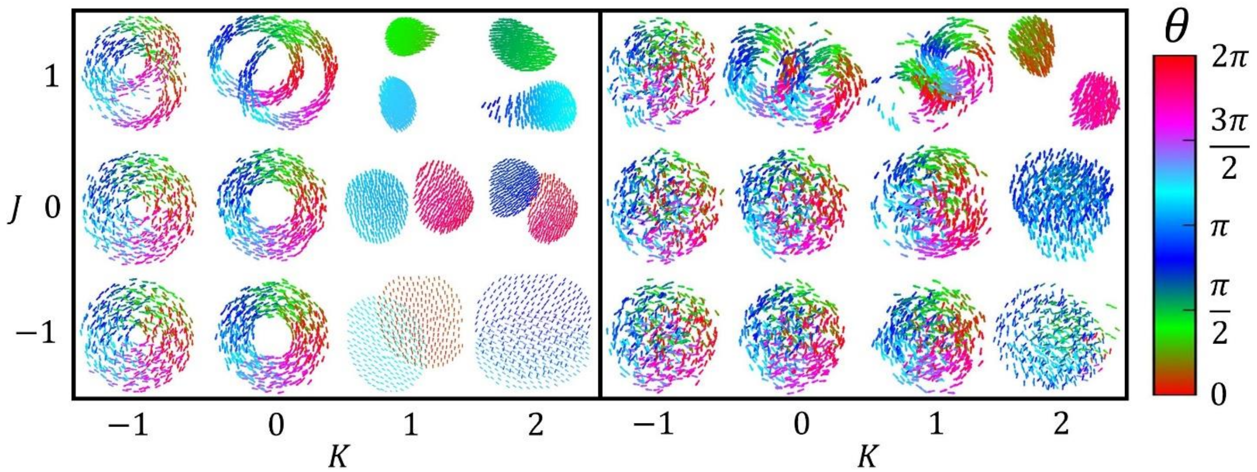


**Supplementary Fig. 17. Collective behaviors of frequency-coupled chiral swarmalators.** $Q_{\dot{x}} = \frac{\pi}{2}\left|\frac{\omega_j}{|\omega_j|} - \frac{\omega_i}{|\omega_i|}\right|, Q_{\dot{\theta}} = \frac{\pi}{4}\left|\frac{\omega_j}{|\omega_j|} - \frac{\omega_i}{|\omega_i|}\right|, F2$ (Left). $F4$ (Right).

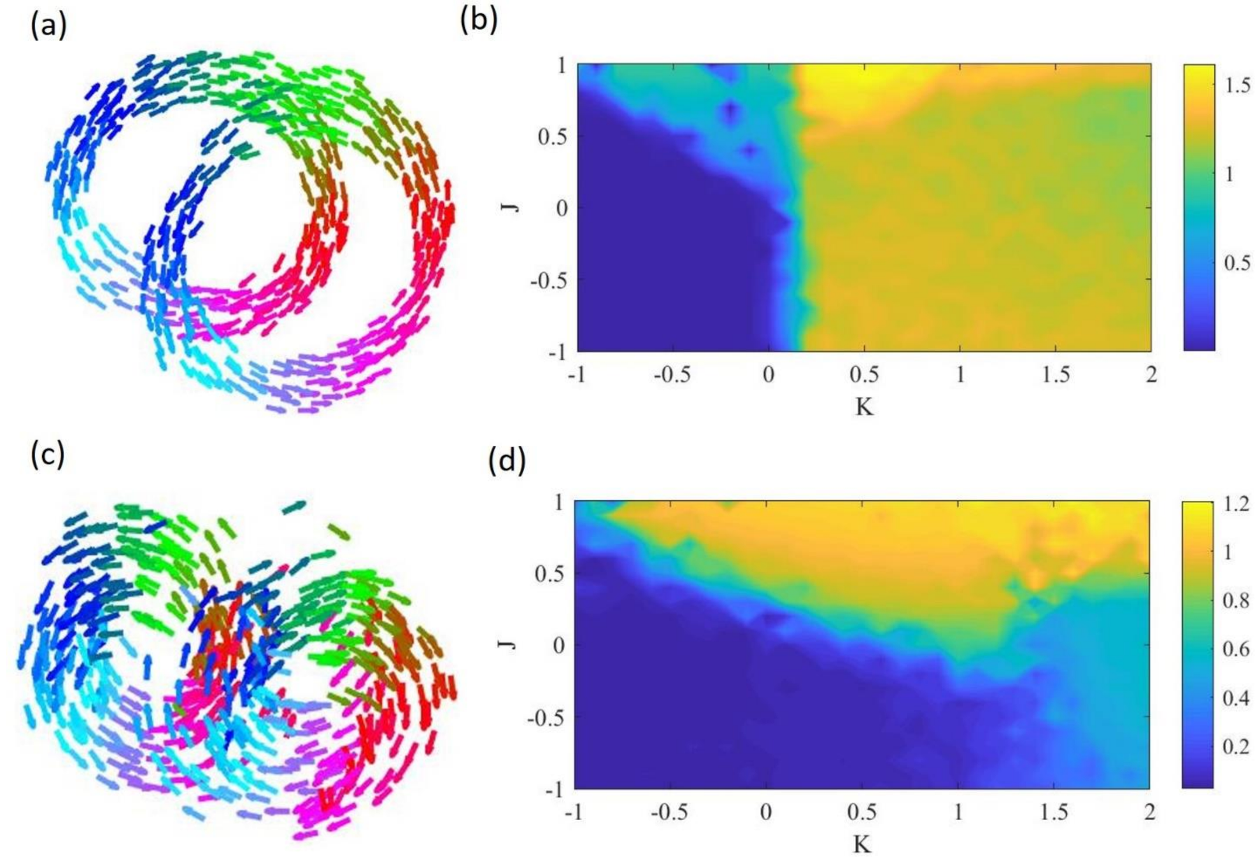


**Supplementary Fig. 18. Characterization of collective behaviors in frequency-coupled chiral swarmalators. (a)** $K = 0, J = 1,\ g(\omega) = \{-\Omega, \Omega\}$ ; **(c)** $K = 0, J = 1,\ F4$ . **(b,d)** Average distance between the natural frequency groups' centroids for **(a)** and **(c)**, respectively.

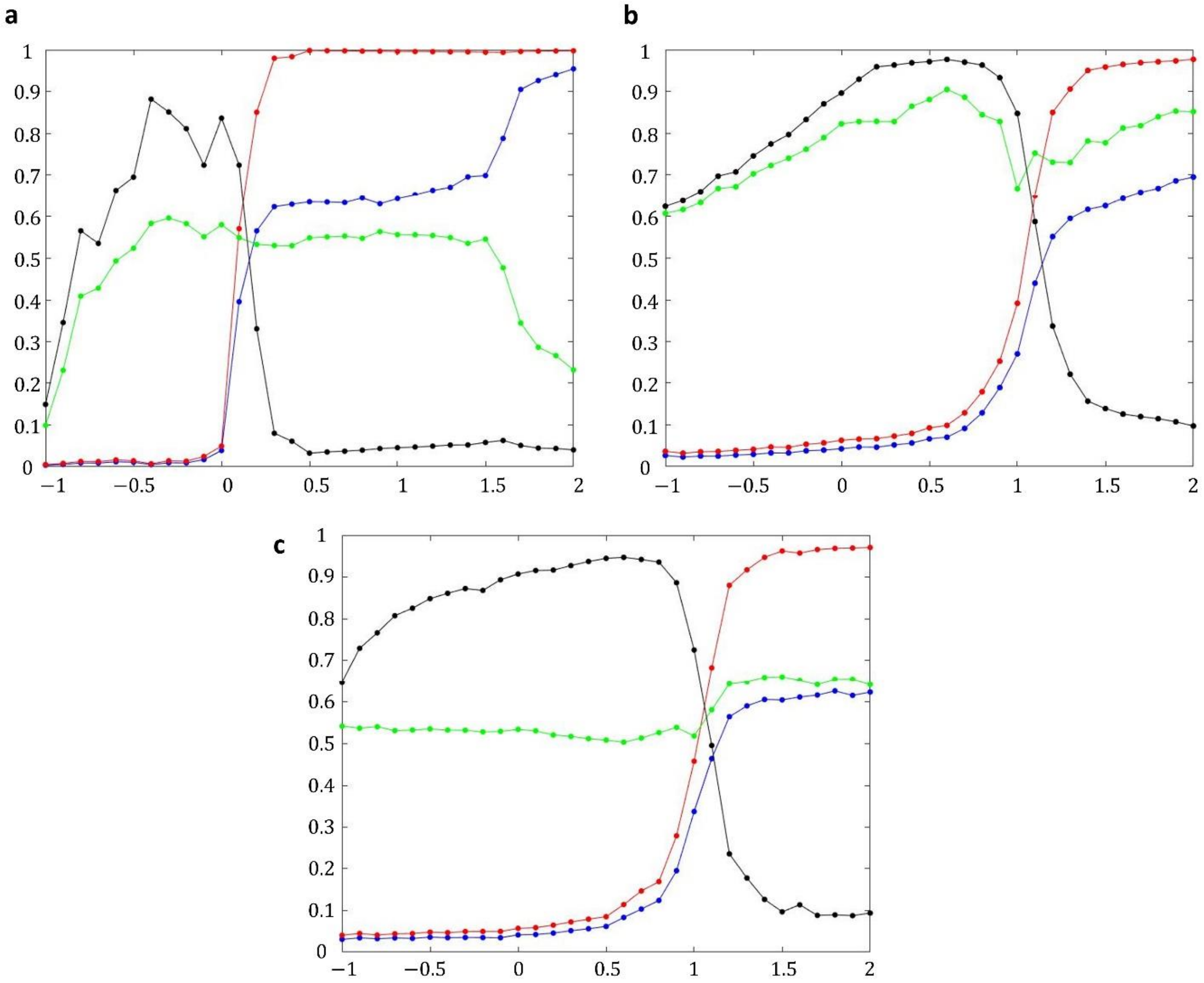


**Supplementary Fig. 19. Order parameters.** Four order parameters are plotted for **(a)** non-chiral swarmalators with the natural frequency distribution $F2$, **(b)** revolving swarmalators with the natural frequency distribution $F3$, and **(c)** frequency-coupled revolving swarmalators with the distribution $F4$. The four order parameters are global phase coherence (blue), frequency group phase coherence (red), global spatial phase order (green), frequency group spatial phase order (black).

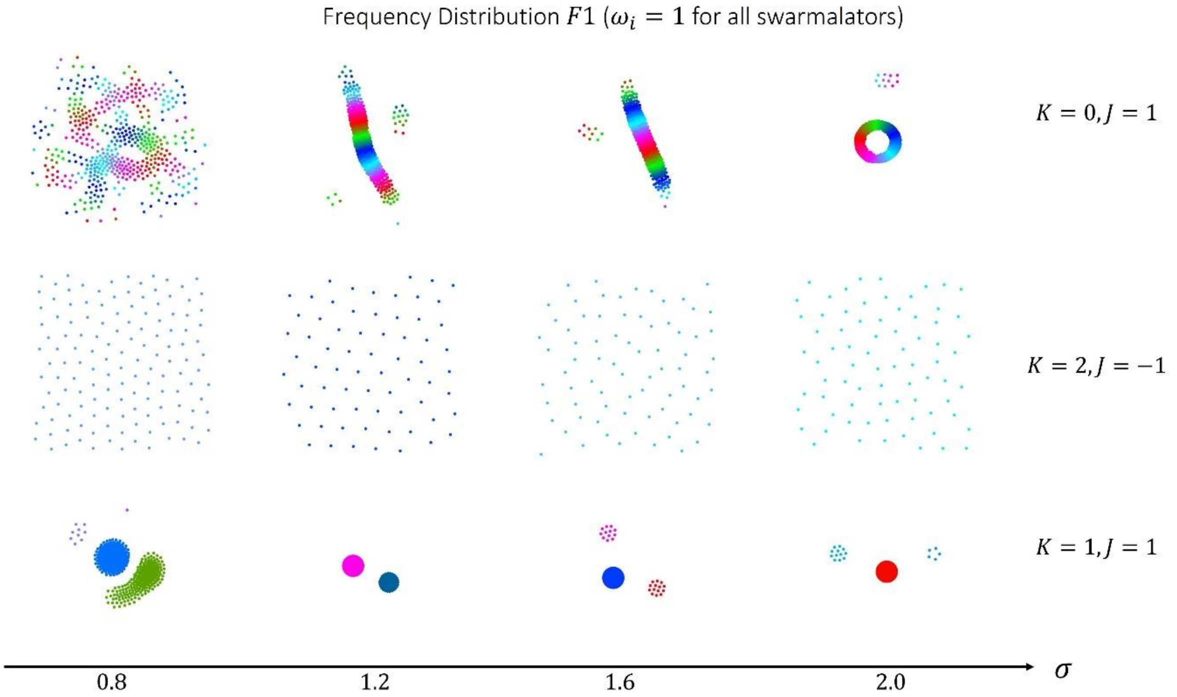


**Supplementary Fig. 20. Collective behaviors of locally coupled non-chiral swarmalators with one natural frequency and no frequency coupling.** The $x$-axis increases the coupling distance ($\sigma$). (Top) $K = 0, J = 1$. (Middle) $K = 2, J = -1$. (Bottom) $K = 1, J = 1$.

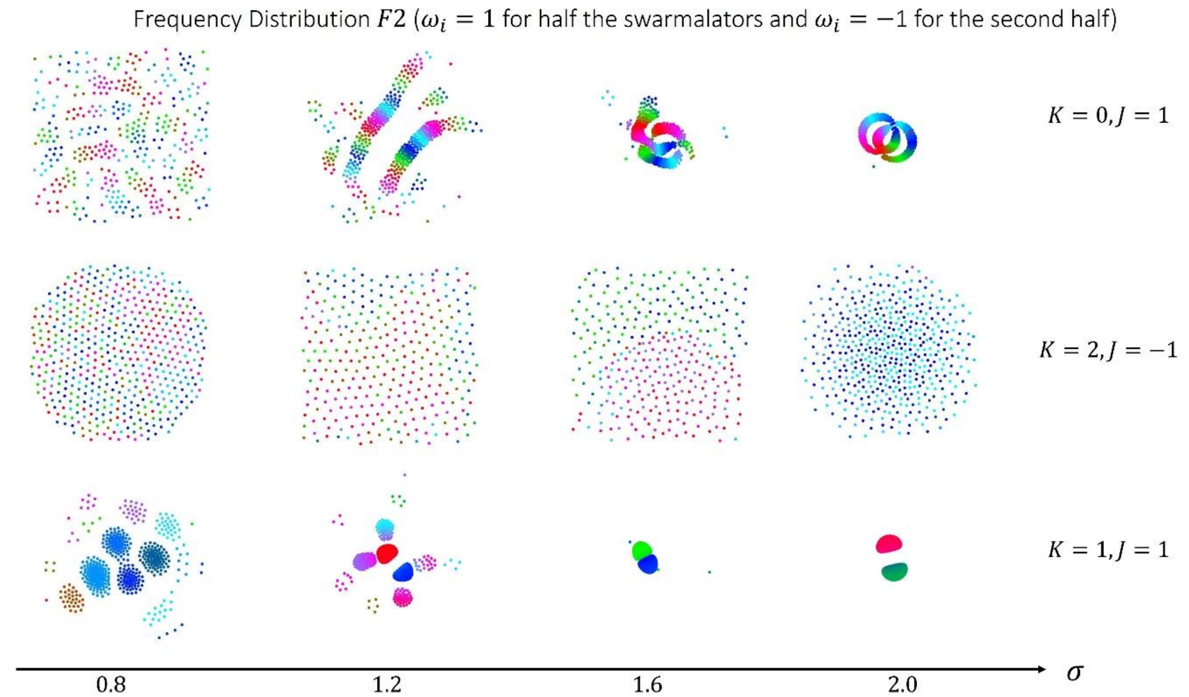


**Supplementary Fig. 21. Collective behaviors of locally coupled non-chiral swarmalators with two natural frequencies and no frequency coupling.** The $x$-axis increases the coupling distance ($\sigma$). (Top) $K = 0, J = 1$. (Middle) $K = 2, J = -1$. (Bottom) $K = 1, J = 1$.

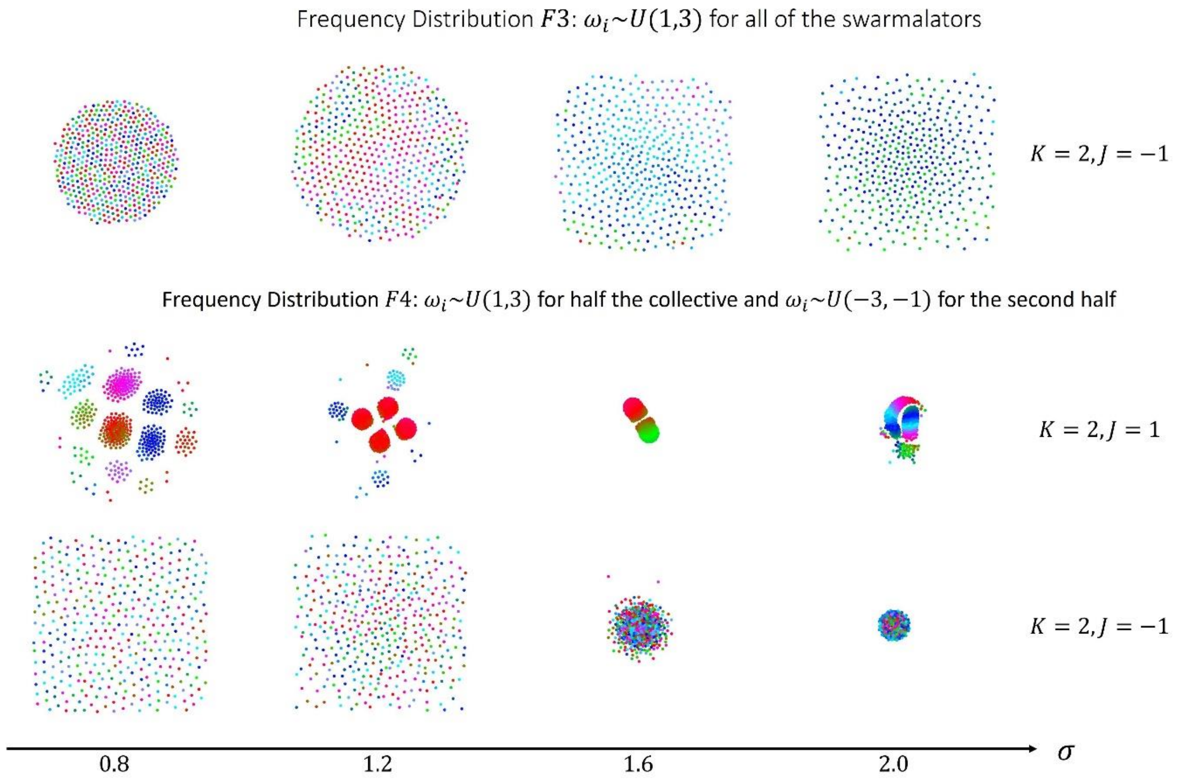


**Supplementary Fig. 22. Collective behaviors of locally coupled non-chiral swarmalators with a natural frequency spread and no frequency coupling.** The $x$-axis increases the coupling distance ($\sigma$). (Top) $K = 2, J = -1$. (Middle) $K = 2, J = 1$. (Bottom) $K = 2, J = -1$.

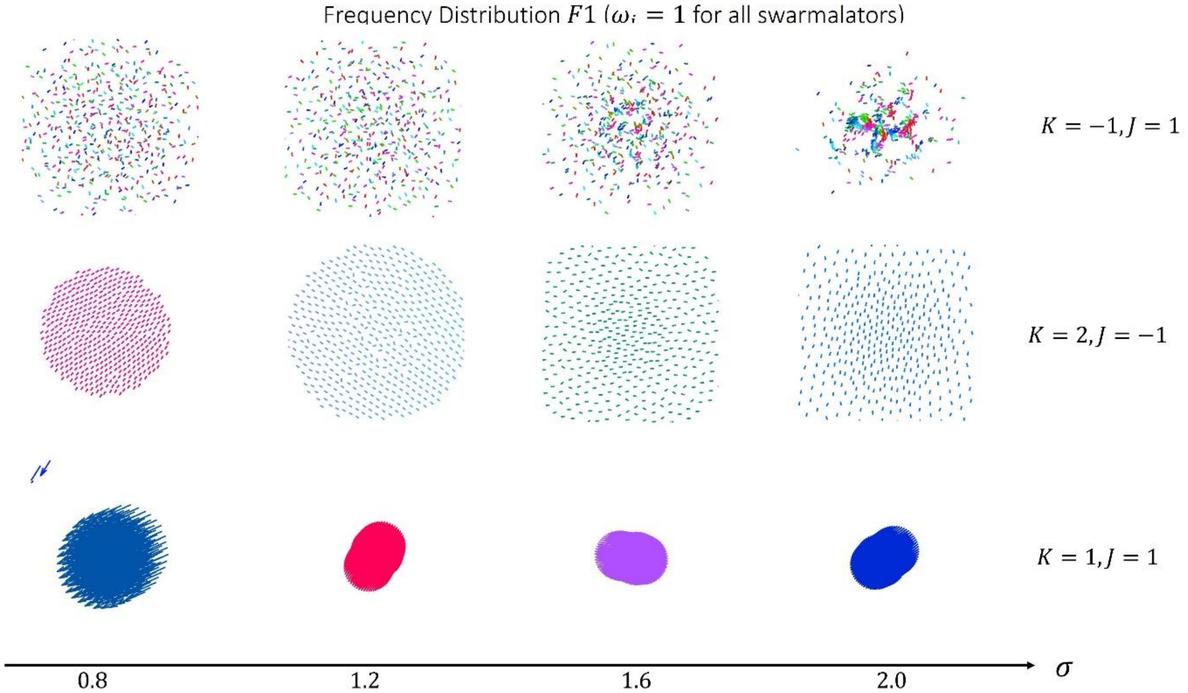


**Supplementary Fig. 23. Collective behaviors of locally coupled chiral swarmalators with one natural frequency and no frequency coupling.** The $x$-axis increases the coupling distance ($\sigma$) and is normalized by the maximum radius of revolution ($R = 1$). (Top) $K = 0, J = 1$. (Middle) $K = 2, J = -1$. (Bottom) $K = 1, J = 1$.

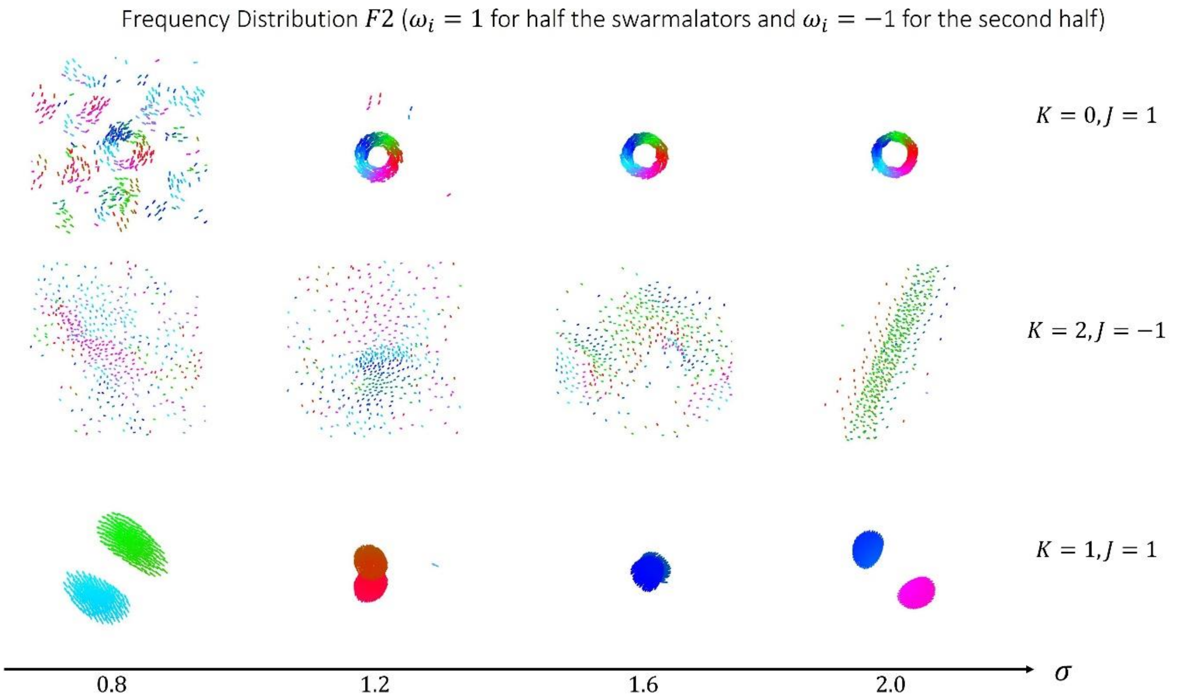


**Supplementary Fig. 24. Collective behaviors of locally coupled chiral swarmalators with one natural frequency and no frequency coupling.** The $x$-axis increases the coupling distance ($\sigma$) and is normalized by the maximum radius of revolution ($R = 1$). (Top) $K = 0, J = 1$. (Middle) $K = 2, J = -1$. (Bottom) $K = 1, J = 1$.

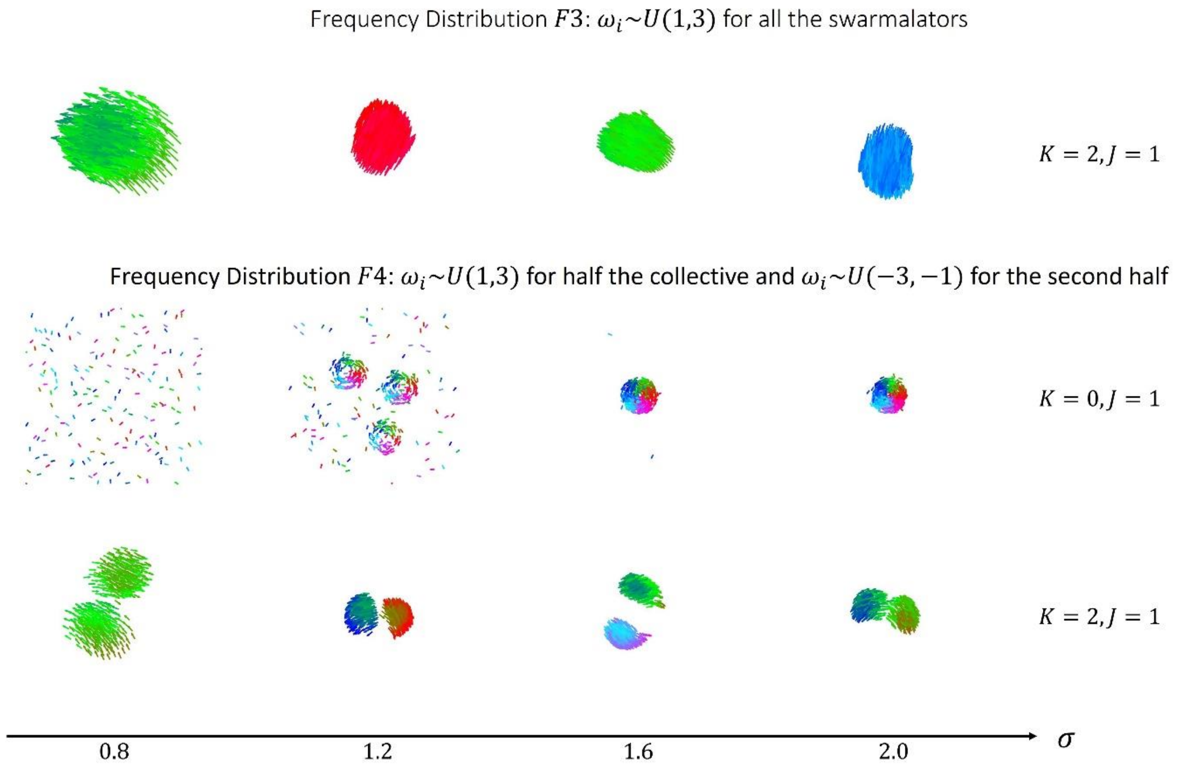


**Supplementary Fig. 25. Collective behaviors of locally coupled chiral swarmalators with a natural frequency spread and no frequency coupling.** The $x$-axis increases the coupling distance ($\sigma$) and is normalized by the maximum radius of revolution ($R = 1$). (Top) $K = 2, J = 1$. (Middle) $K = 0, J = 1$. (Bottom) $K = 2, J = 1$.

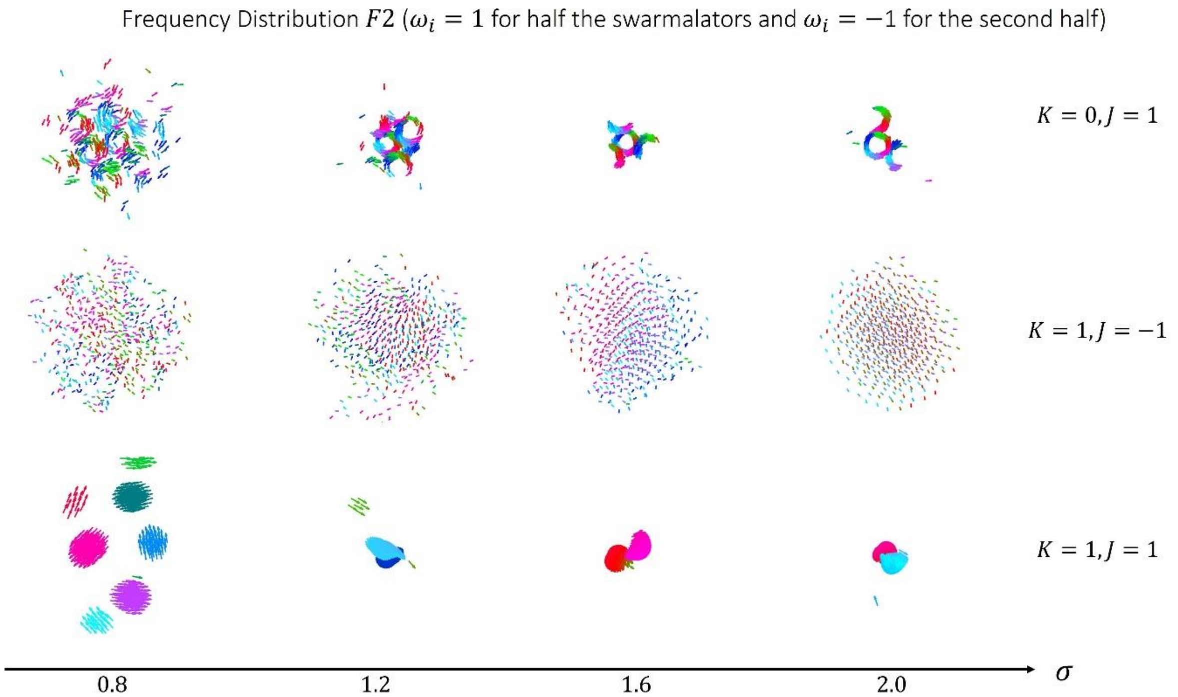


**Supplementary Fig. 26. Collective behaviors of locally coupled chiral swarmalators with no natural frequency spread and frequency coupled.** The $x$-axis increases the coupling distance ($\sigma$). (Top) $K = 0, J = 1$. (Middle) $K = 1, J = -1$. (Bottom) $K = 1, J = 1$.

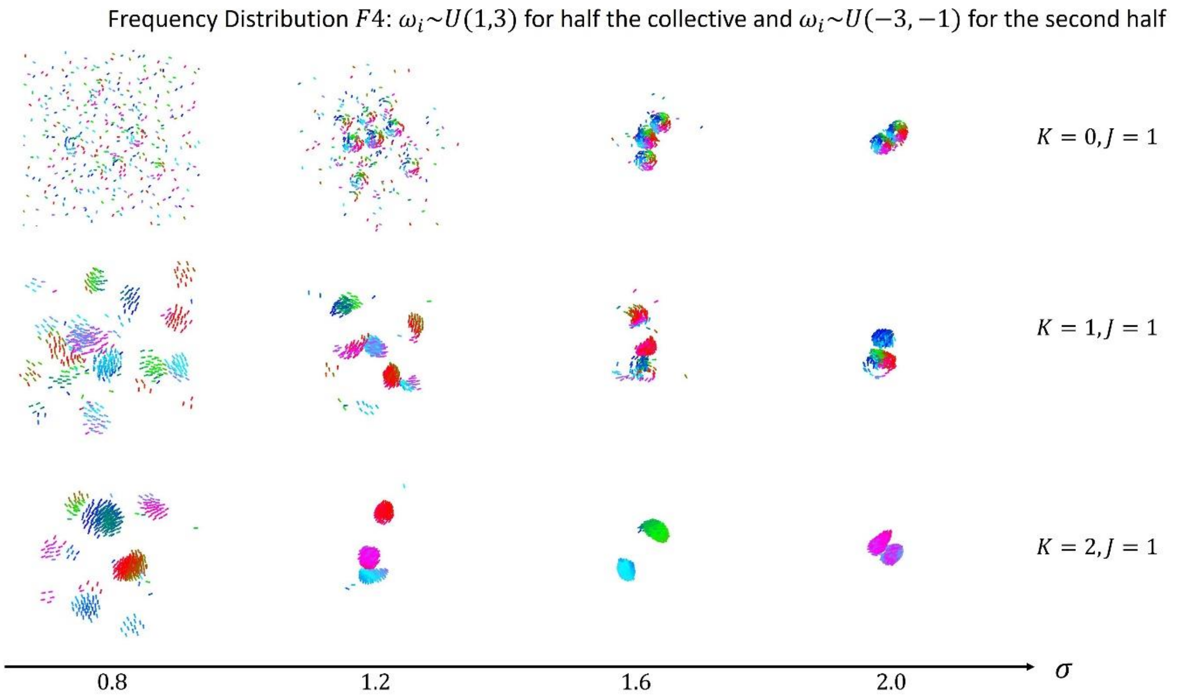


**Supplementary Fig. 27. Collective behaviors of locally coupled chiral swarmalators with a natural frequency spread and frequency coupling.** The $x$-axis increases the coupling distance ($\sigma$). (Top) $K = 0, J = 1$. (Middle) $K = 1, J = 1$. (Bottom) $F4$. $K = 2, J = 1$.

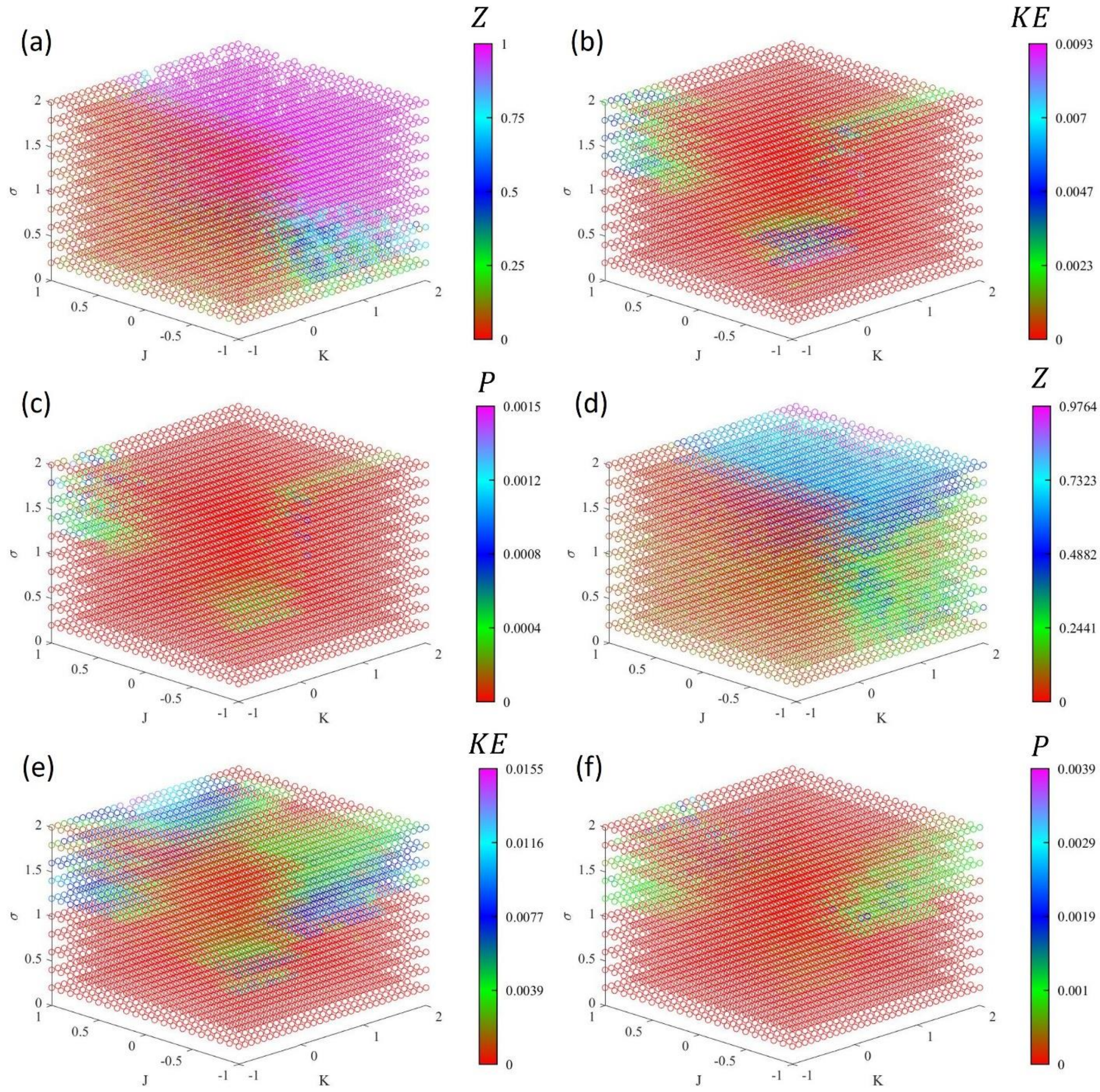


**Supplementary Fig. 28. 3D Maps for locally coupled non-chiral swarmalators with no frequency spread and no frequency coupling.** (a-c) There is a single natural frequency, $\omega_i = 1$, throughout the collective. (d-f) Half the collective has $\omega_i = -1$ and the other half has $\omega_i = 1$. **(a,d)** Degree of synchrony ($Z$). **(b,e)** Kinetic energy ($KE$). **(c,f)** Linear momentum ($P$).

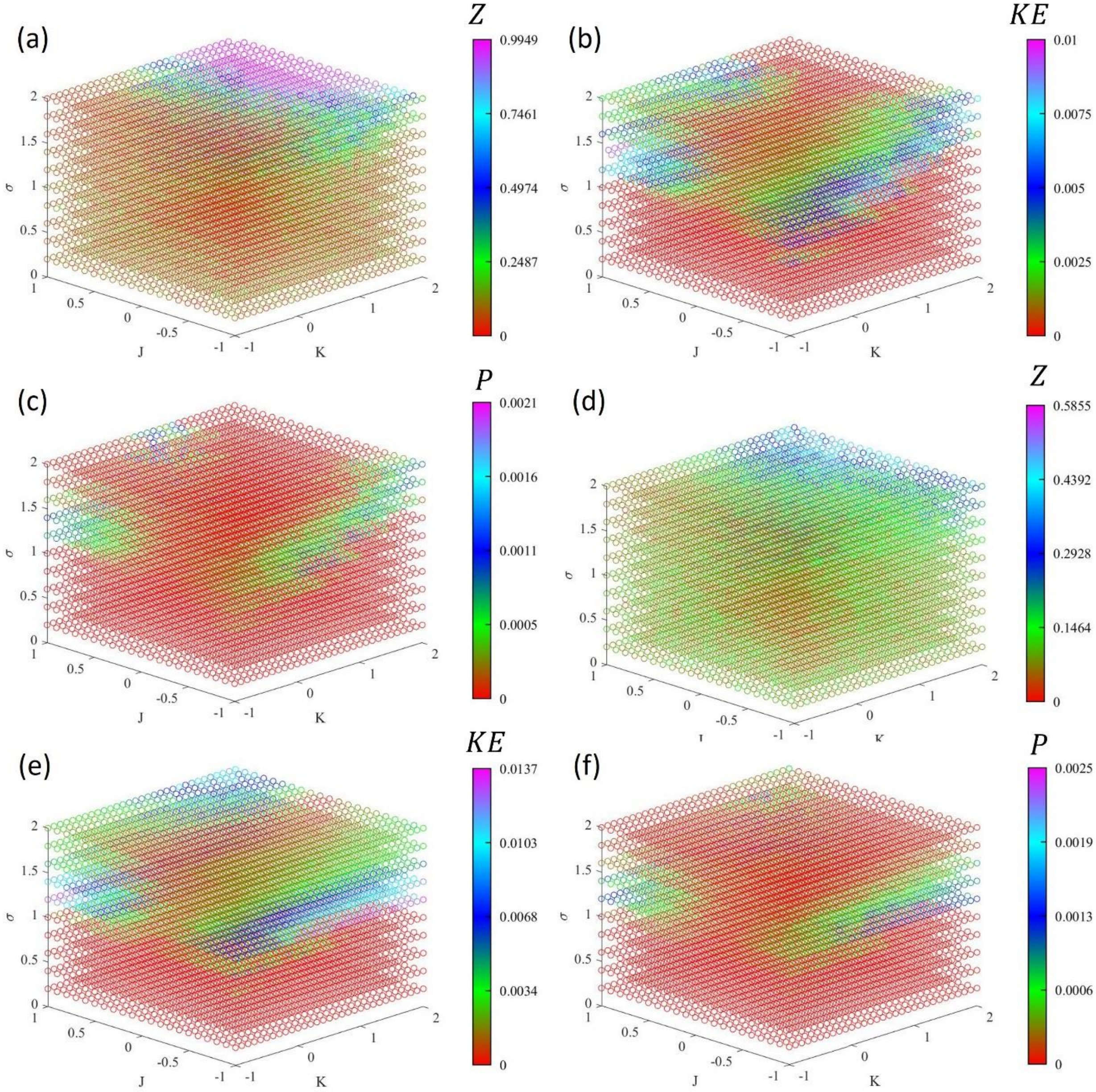


**Supplementary Fig. 29. 3D Maps for locally coupled non-chiral swarmalators with a frequency spread and no frequency coupling.** (a-c) All swarmalators have their natural frequency randomly selected from single uniform distribution, such that $\omega_i \sim U(\Omega, 3\Omega)$. (d-f) Half the collective has their natural frequencies randomly selected from the uniform distribution $U(1,3)$ and the second half have their natural frequency selected from the uniform distribution $U(-3,-1)$. **(a,d)** Degree of synchrony ($Z$). **(b,e)** Kinetic energy ($KE$). **(c,f)** Linear momentum ($P$).

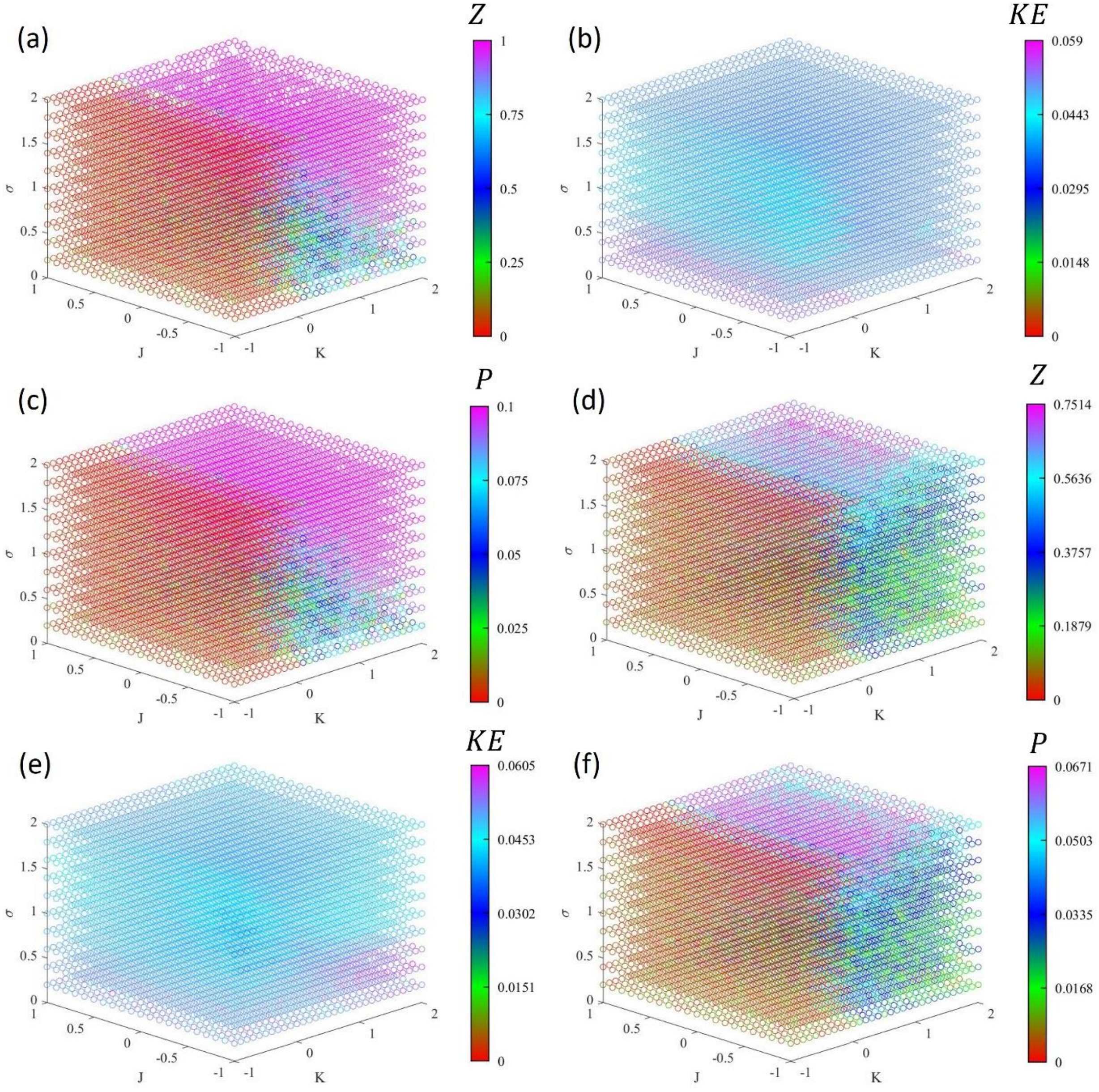


**Supplementary Fig. 30. 3D Maps for locally coupled chiral swarmalators with no frequency spread and no frequency coupling.** (a-c) There is a single natural frequency, $\omega_i = 1$, throughout the collective. (d-f) Half the collective has $\omega_i = -1$ and the other half has $\omega_i = 1$. **(a,d)** Degree of synchrony ($Z$). **(b,e)** Kinetic energy ($KE$). **(c,f)** Linear momentum ($P$).

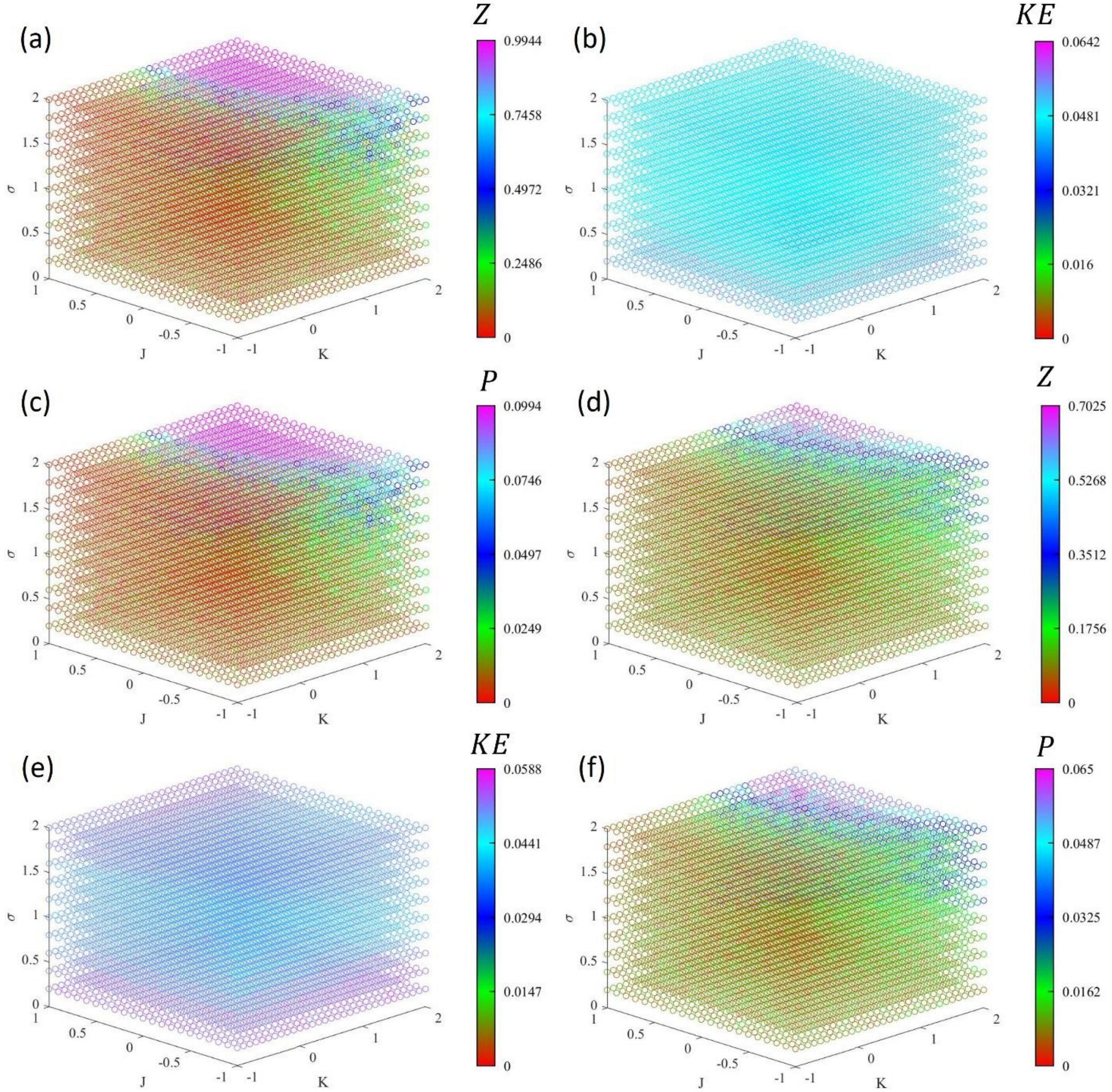


**Supplementary Fig. 31. 3D Maps for locally coupled chiral swarmalators with a frequency spread and no frequency coupling.** (a-c) All swarmalators have their natural frequency randomly selected from single uniform distribution, such that $\omega_i \sim U(\Omega, 3\Omega)$. (d-f) Half the collective has their natural frequencies randomly selected from the uniform distribution $U(1, 3)$ and the second half have their natural frequency selected from the uniform distribution $U(-3, -1)$. **(a,d)** Degree of synchrony ($Z$). **(b,e)** Kinetic energy ($KE$). **(c,f)** Linear momentum ($P$).

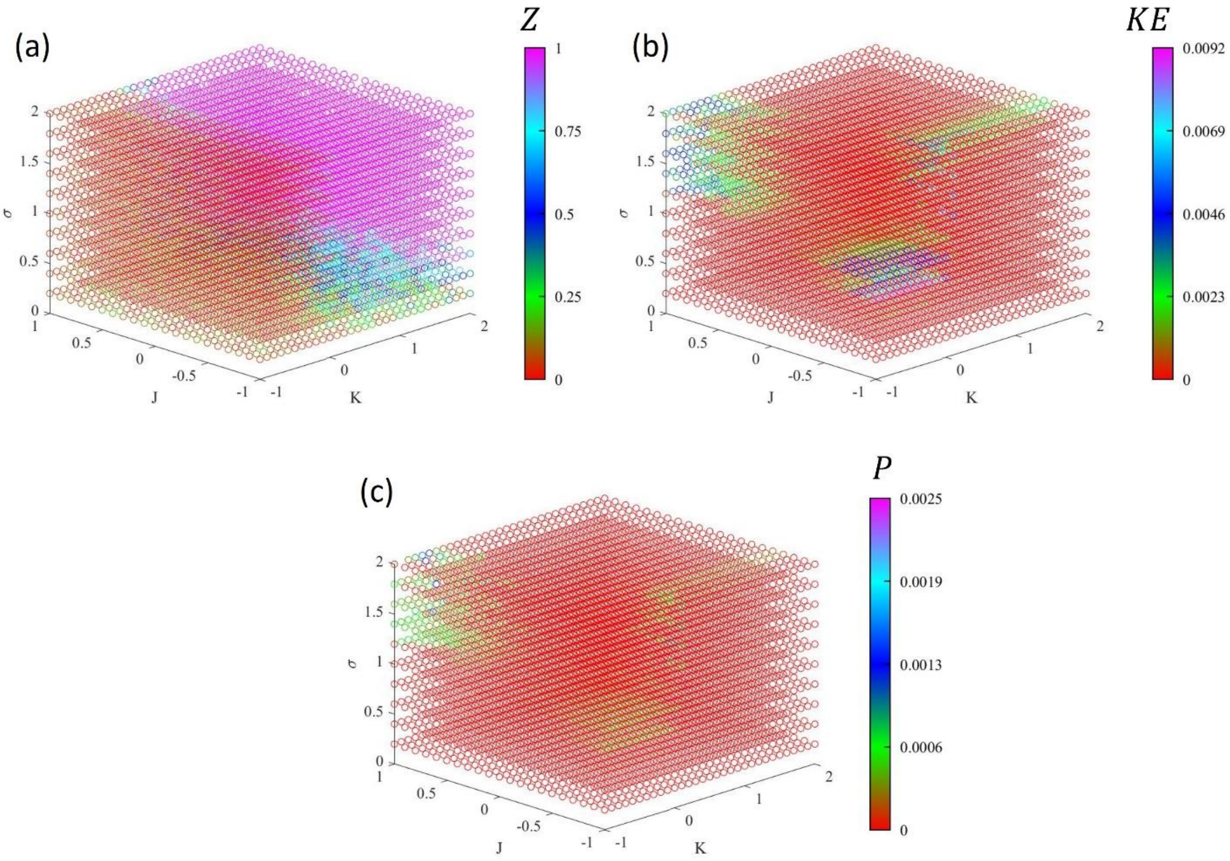


**Supplementary Fig. 32. 3D Maps for locally coupled non-chiral swarmalators with no frequency spread and frequency-coupled.** Half the collective has $\omega_i = -1$ and the other half has $\omega_i = 1$. **(a)** Degree of synchrony ($Z$). **(b)** Kinetic energy ($KE$). **(c)** Linear momentum ($P$).

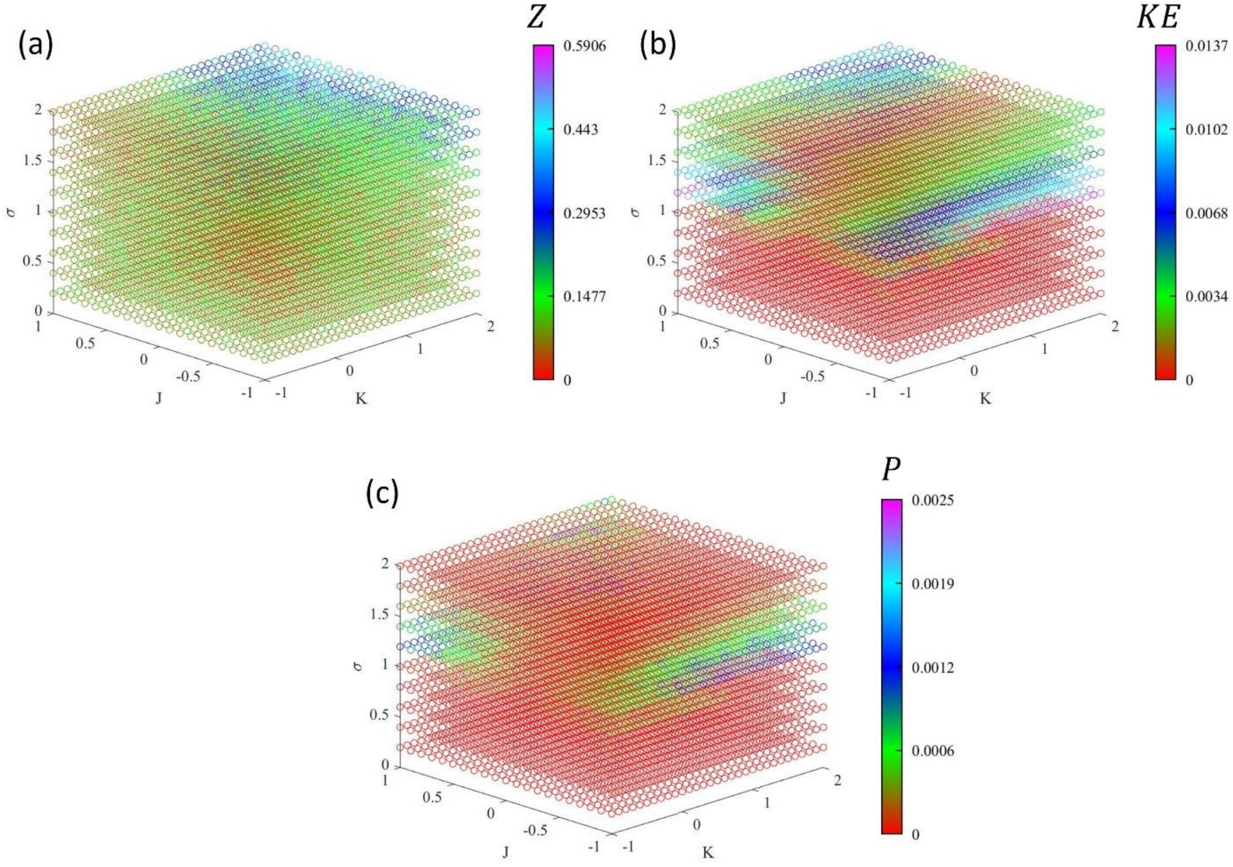


**Supplementary Fig. 33. 3D Maps for locally coupled non-chiral swarmalators with a frequency spread and frequency-coupled.** Half the collective has their natural frequencies randomly selected from the uniform distribution $U(1, 3)$ and the second half have their natural frequency selected from the uniform distribution $U(-3, -1)$. **(a)** Degree of synchrony ($Z$). **(b)** Kinetic energy ($KE$). **(c)** Linear momentum ($P$).

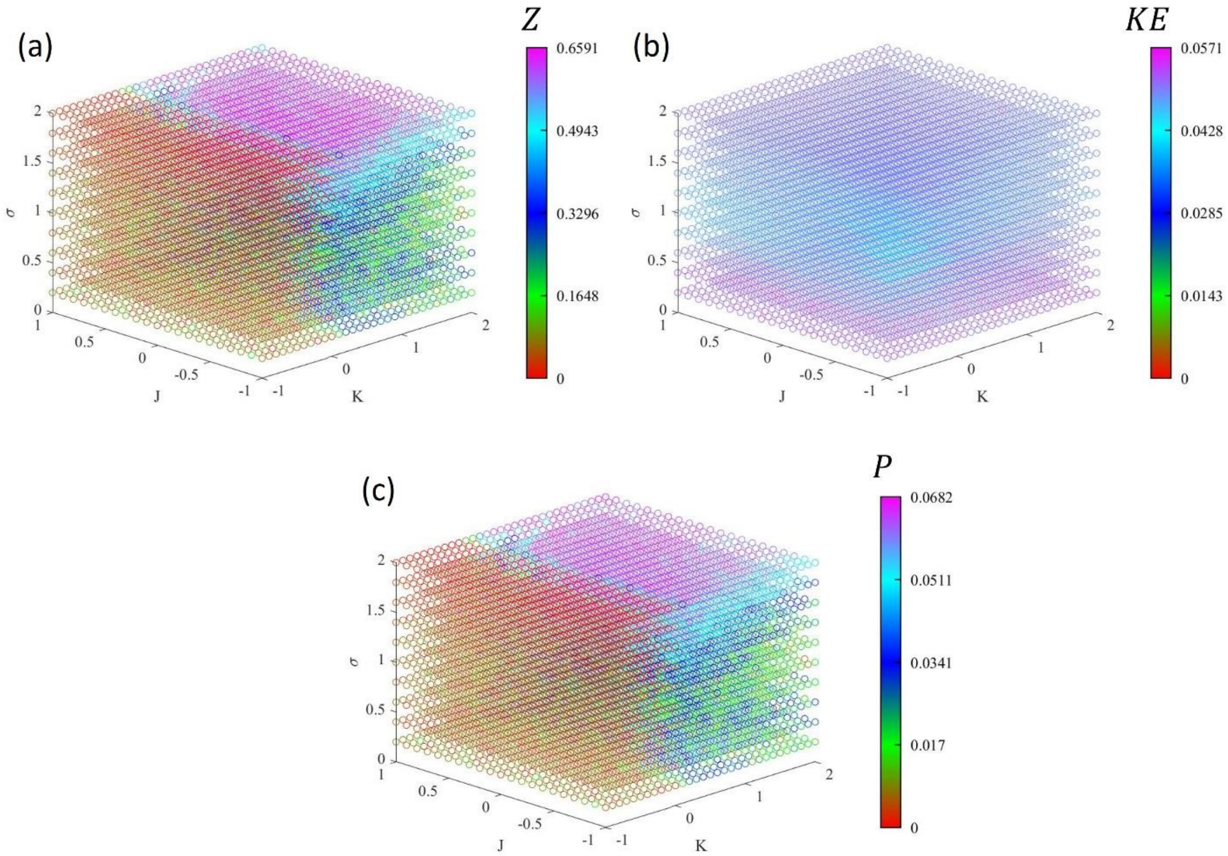


**Supplementary Fig. 34. 3D Maps for locally coupled chiral swarmalators with no frequency spread and frequency-coupled.** Half the collective has $\omega_i = -1$ and the other half has $\omega_i = 1$. **(a)** Degree of synchrony ($Z$). **(b)** Kinetic energy ($KE$). **(c)** Linear momentum ($P$).

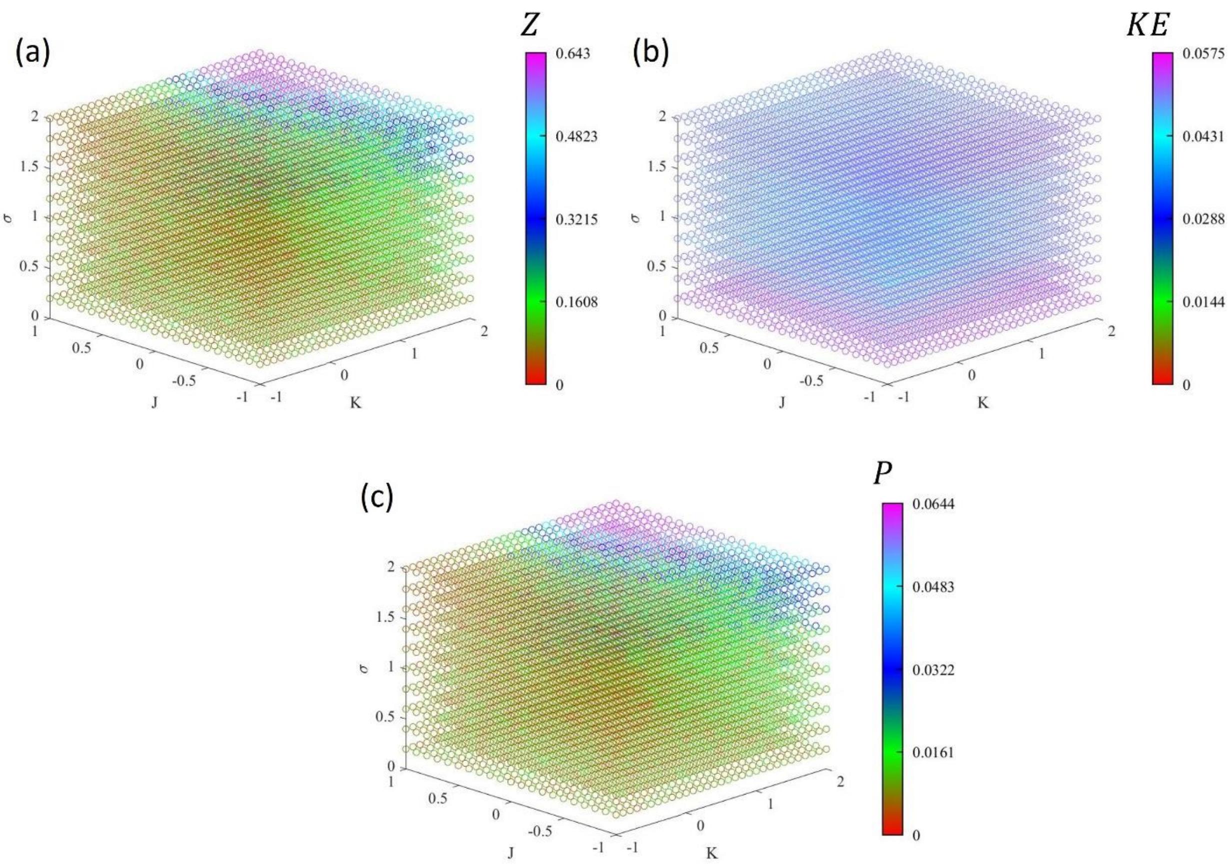


**Supplementary Fig. 35. 3D Maps for locally coupled chiral swarmalators with a frequency spread and frequency-coupled.** Half the collective has their natural frequencies randomly selected from the uniform distribution $U(1,3)$ and the second half have their natural frequency selected from the uniform distribution $U(-3,-1)$. **(a)** Degree of synchrony ($Z$). **(b)** Kinetic energy ($KE$). **(c)** Linear momentum ($P$).

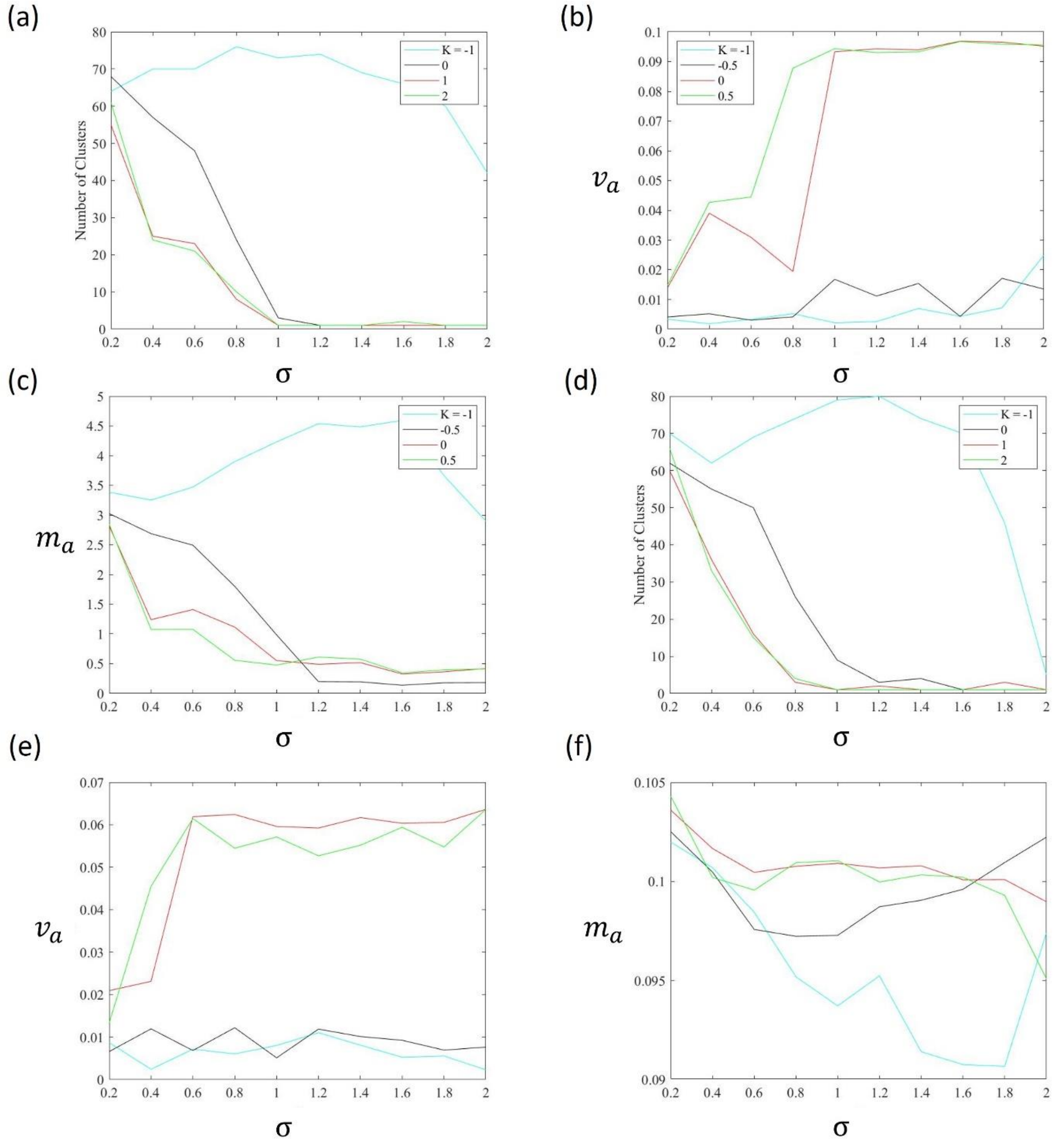


**Supplementary Fig. 36. Cluster count and motion characterization of locally coupled chiral swarmalators with no frequency spread and no frequency coupling.** (a-c) There is a single natural frequency, $\omega_i = 1$, throughout the collective. (d-f) Half the collective has $\omega_i = -1$ and the other half has $\omega_i = 1$. **(a,d)** Number of clusters. **(b,e)** Normalized velocity ($v_a$). **(c,f)** Normalized angular momentuum ($m_a$).

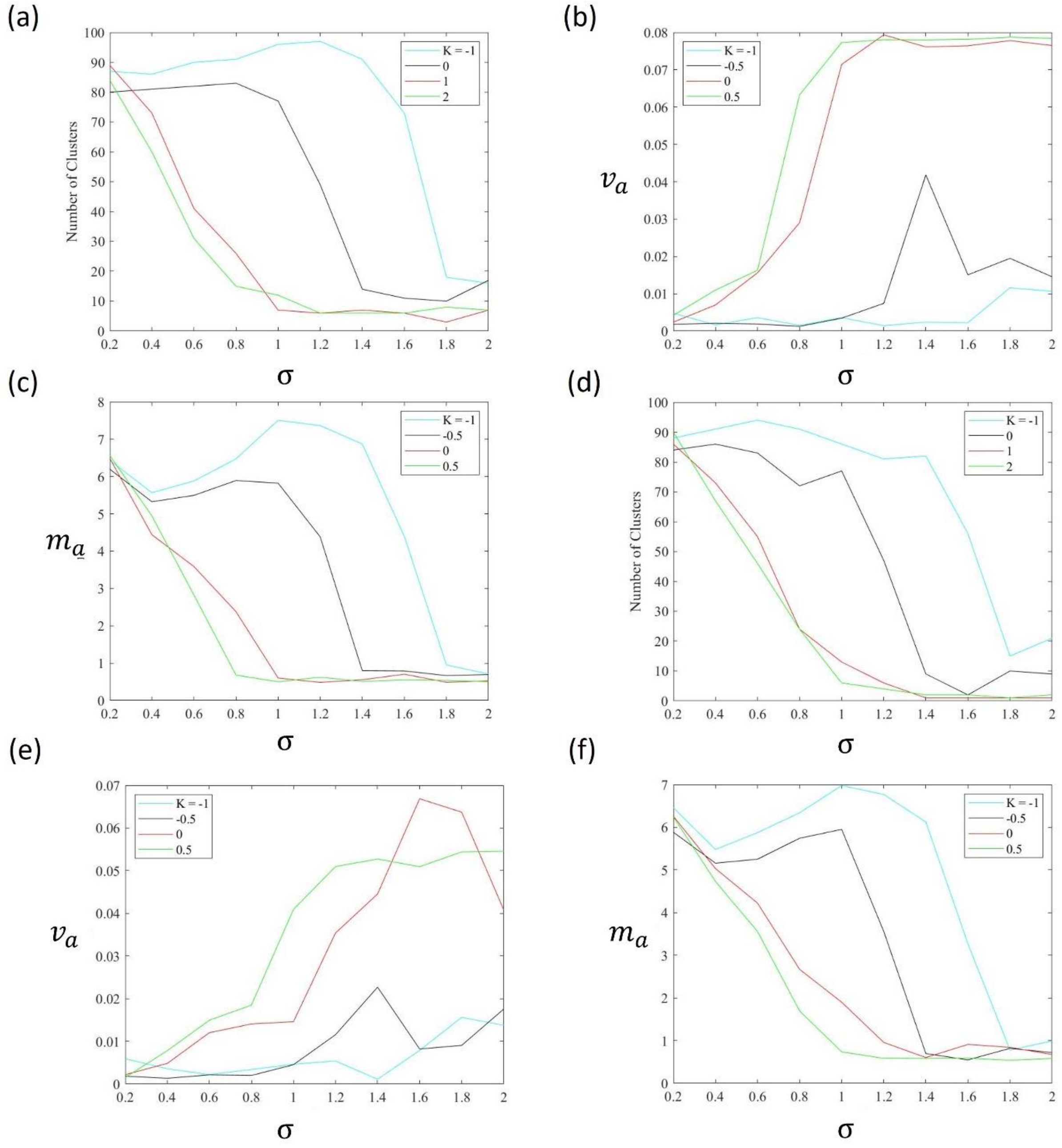


**Supplementary Fig. 37. Cluster count and motion characterization of locally coupled chiral swarmalators with a frequency spread and no frequency coupling.** (a-c) All swarmalators have their natural frequency randomly selected from single uniform distribution, such that $\omega_i \sim U(\Omega, 3\Omega)$. (d-f) Half the collective has their natural frequencies randomly selected from the uniform distribution $U(1, 3)$ and the second half have their natural frequency selected from the uniform distribution $U(-3, -1)$. **(a,d)** Number of clusters. **(b,e)** Normalized velocity ($v_a$). **(c,f)** Normalized angular momentuum ($m_a$).

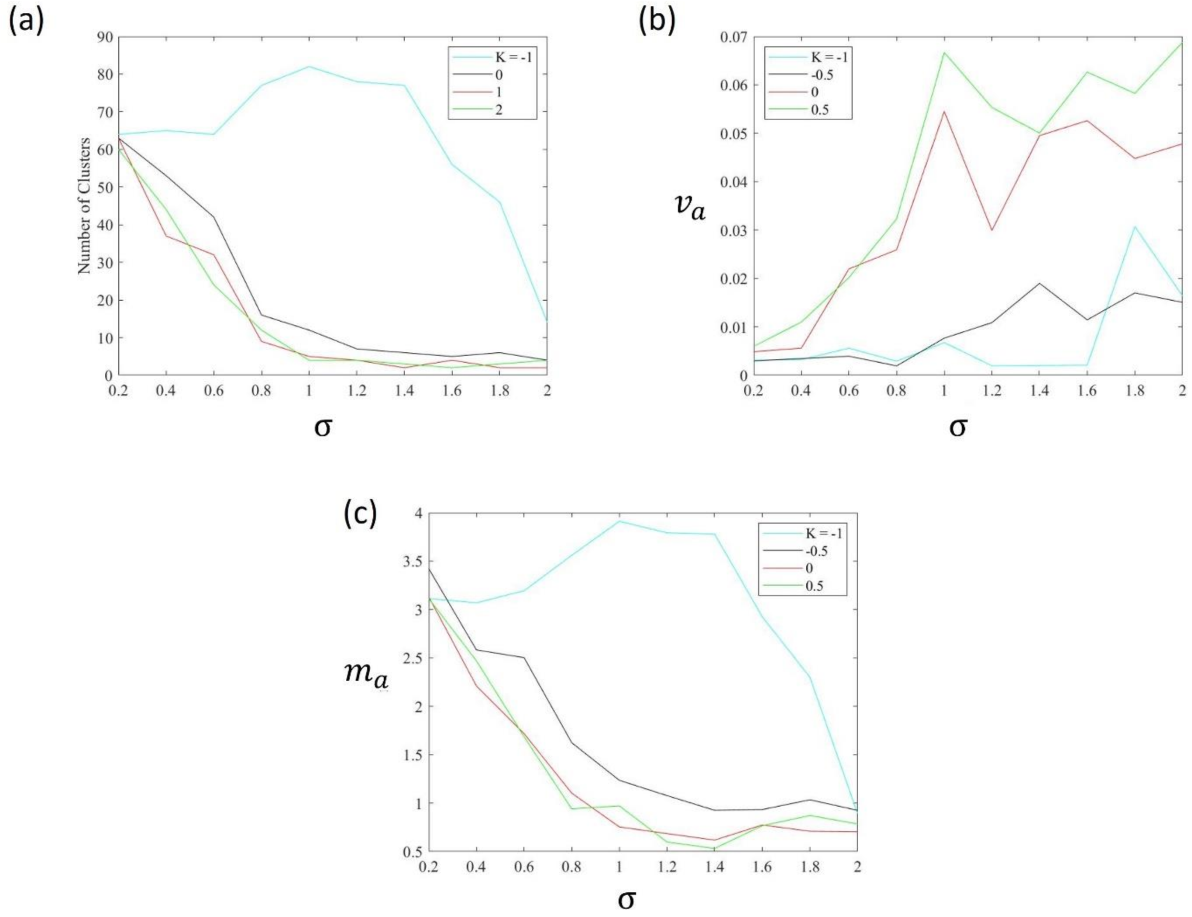


**Supplementary Fig. 38. Cluster count and motion characterization of locally coupled chiral swarmalators with no frequency spread and frequency-coupled.** Half the collective has $\omega_i = -1$ and the other half has $\omega_i = 1$. **(a)** Number of clusters. **(b)** Normalized velocity ($v_a$). **(c)** Normalized angular momentuum ($m_a$).

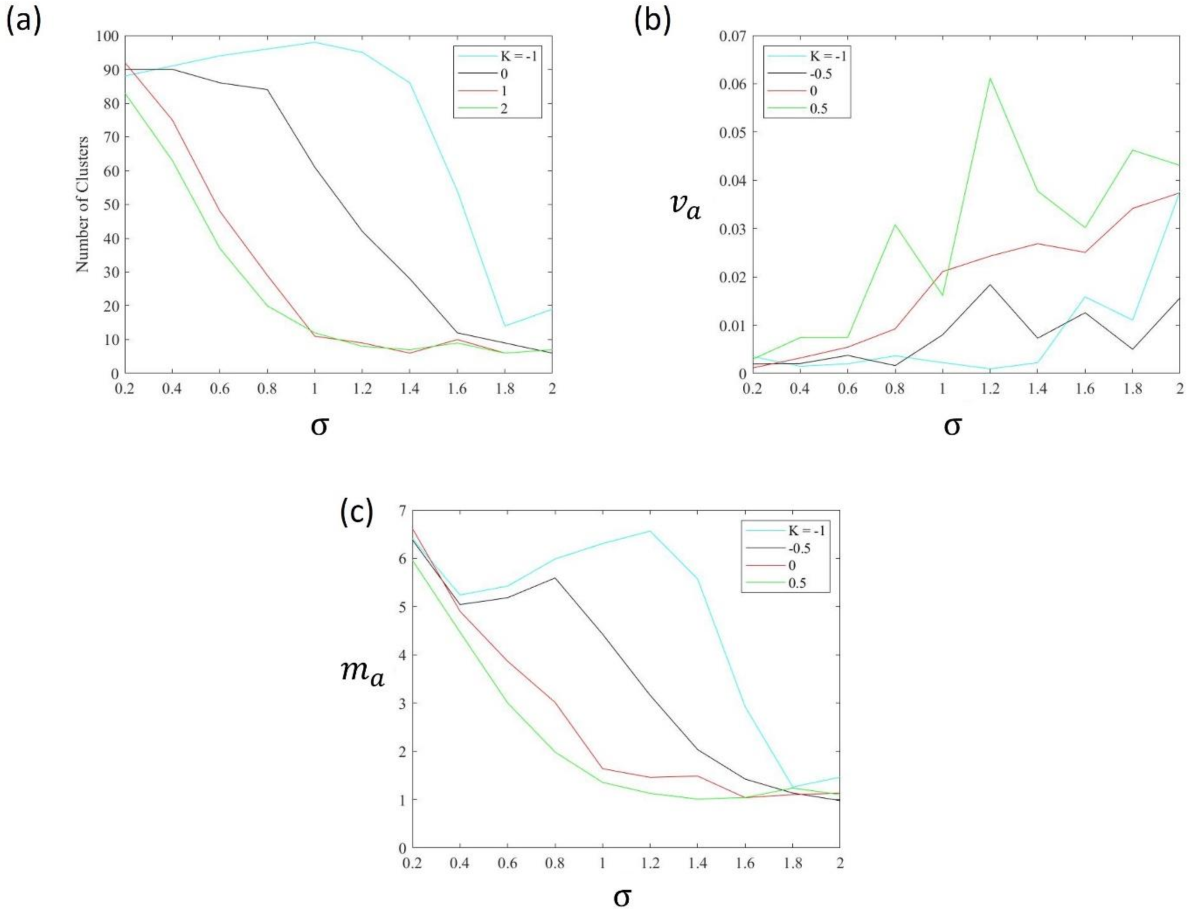


**Supplementary Fig. 39. Cluster count and motion characterization of locally coupled chiral swarmalators with a frequency spread and frequency-coupled.** Half the collective has their natural frequencies randomly selected from the uniform distribution $U(1,3)$ and the second half has their natural frequency selected from the uniform distribution $U(-3,-1)$. **(a)** Number of clusters. **(b)** Normalized velocity ($v_a$). **(c)** Normalized angular momentuum ($m_a$).

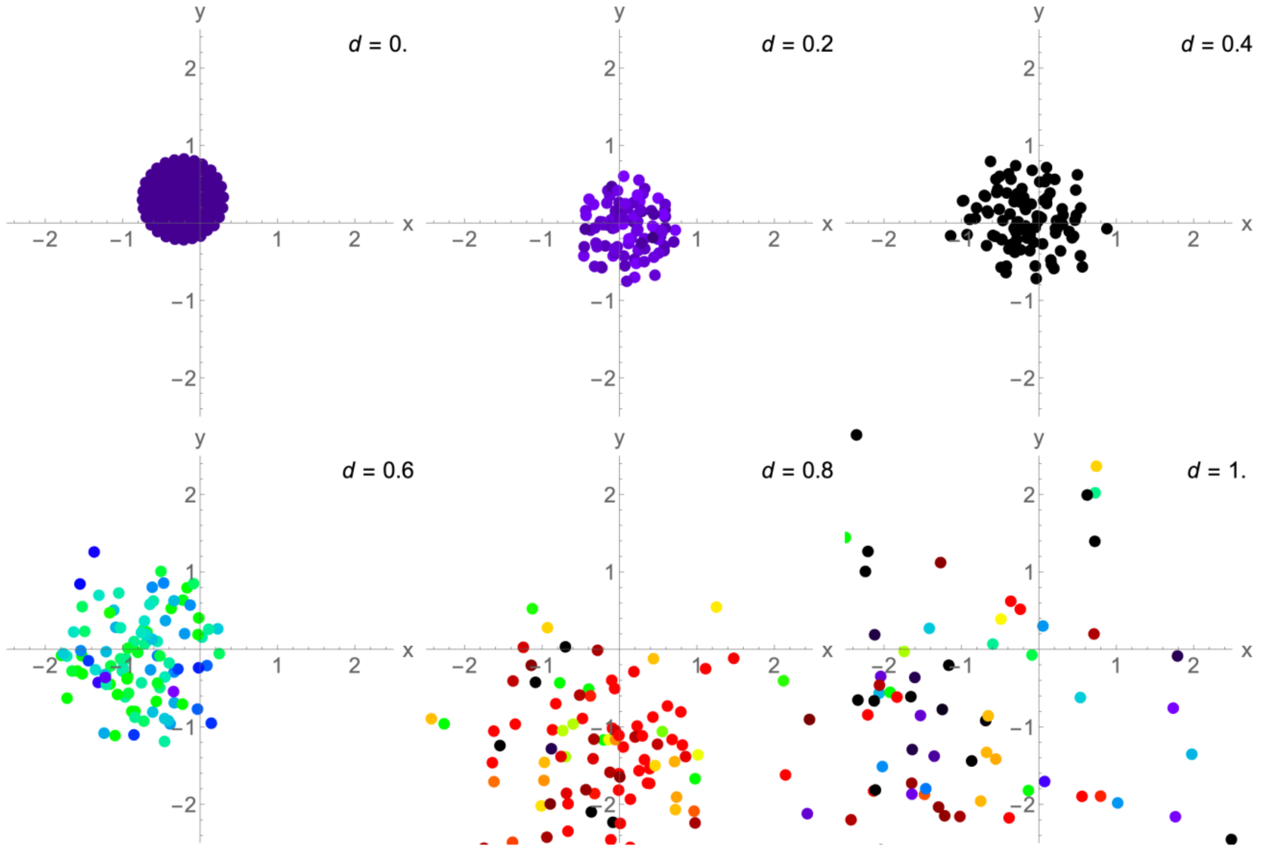


**Supplementary Fig. 40. Sync state for increasing $d$.** System becomes noticeably disordered as $d$ inreases.

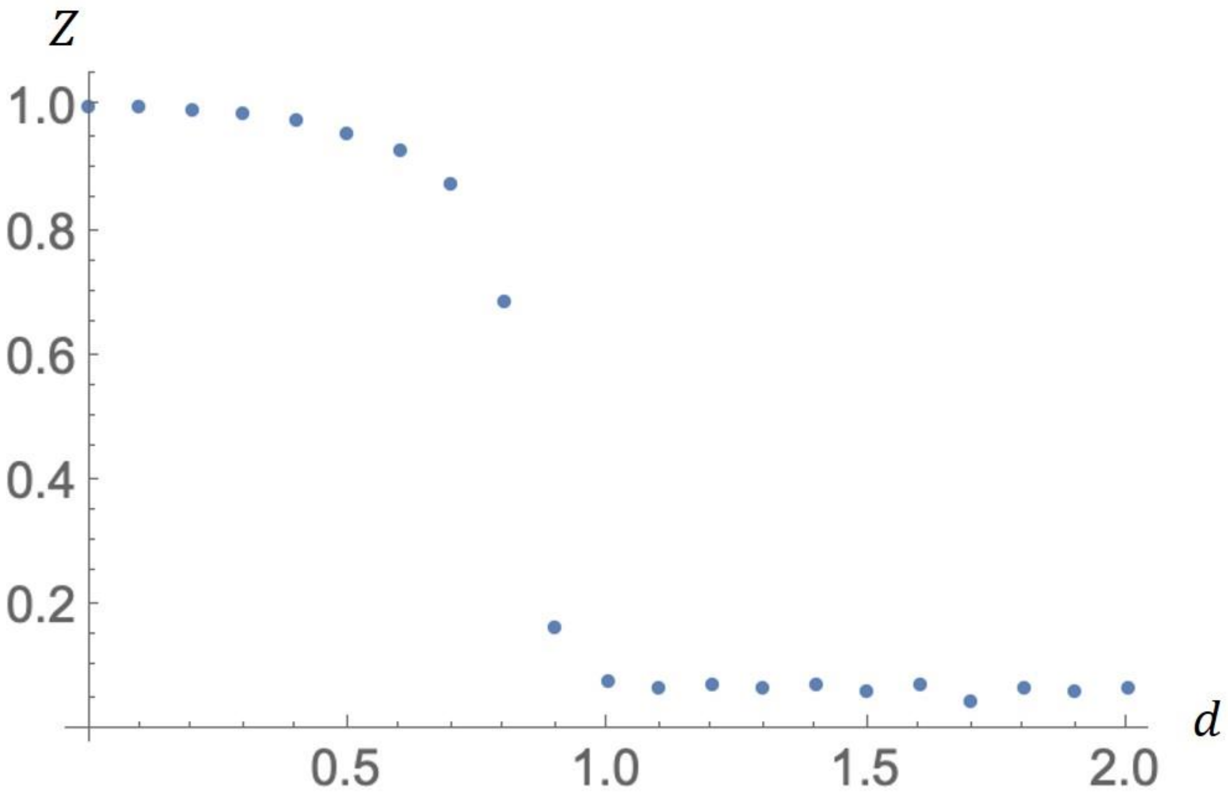


**Supplementary Fig. 41. Phase coherence for increasing $d$ in collectives with global coupling, identical, and non-chiral swarmalators.** On the y-axis, $Z$ decreases to almost zero with increasing $d$.

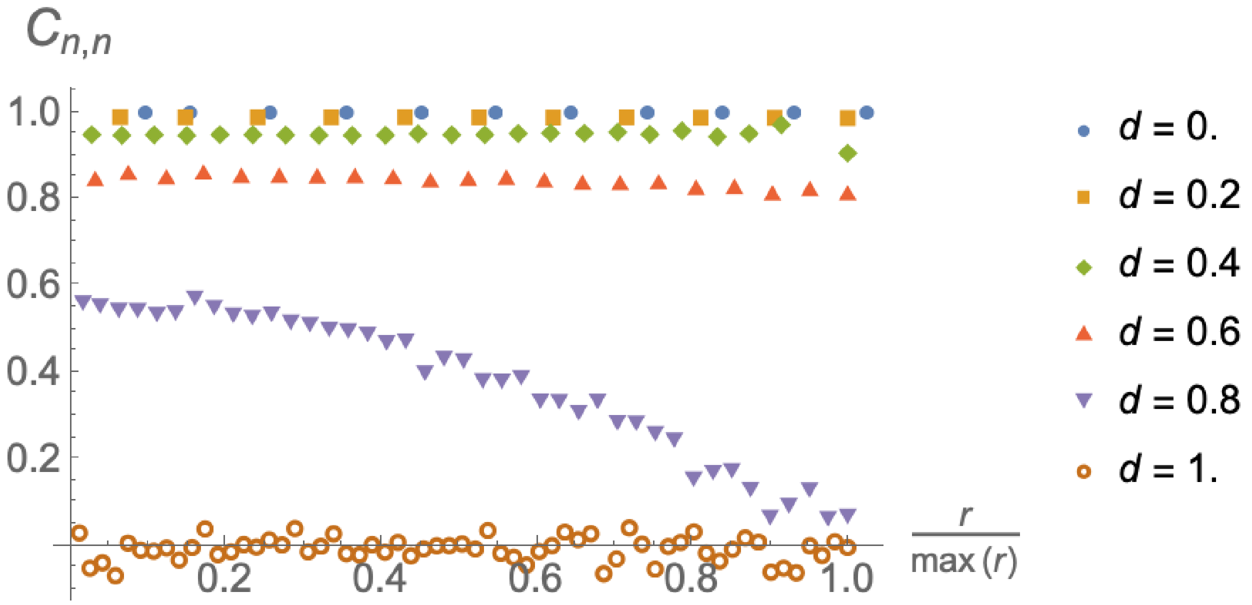


**Supplementary Fig. 42. Phase-phase correlation function.** Phase-phase correlation function $C_{\theta,\theta}(r)$ Pars: $(J, K, \sigma) = (1, 1, 10)$. $(dt, T, N) = (0.1, 200, 300)$. The first half of the data is dropped as transient. Each data point is the average of five simulations.

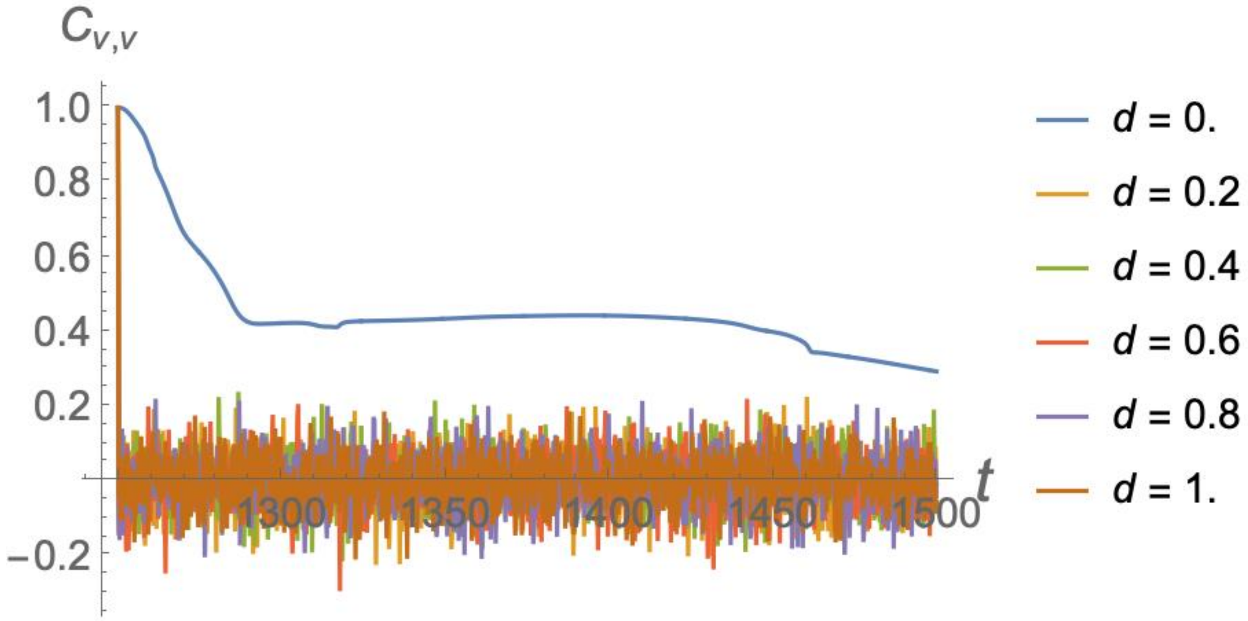


**Supplementary Fig. 43. Velocity auto-correlation function.** Simulation parameters: $(dt, T, N) = (0.1, 500, 200)$. The first half of data was dropped as transients (i.e. $\tau = NT/2$ in Eq. (2)).

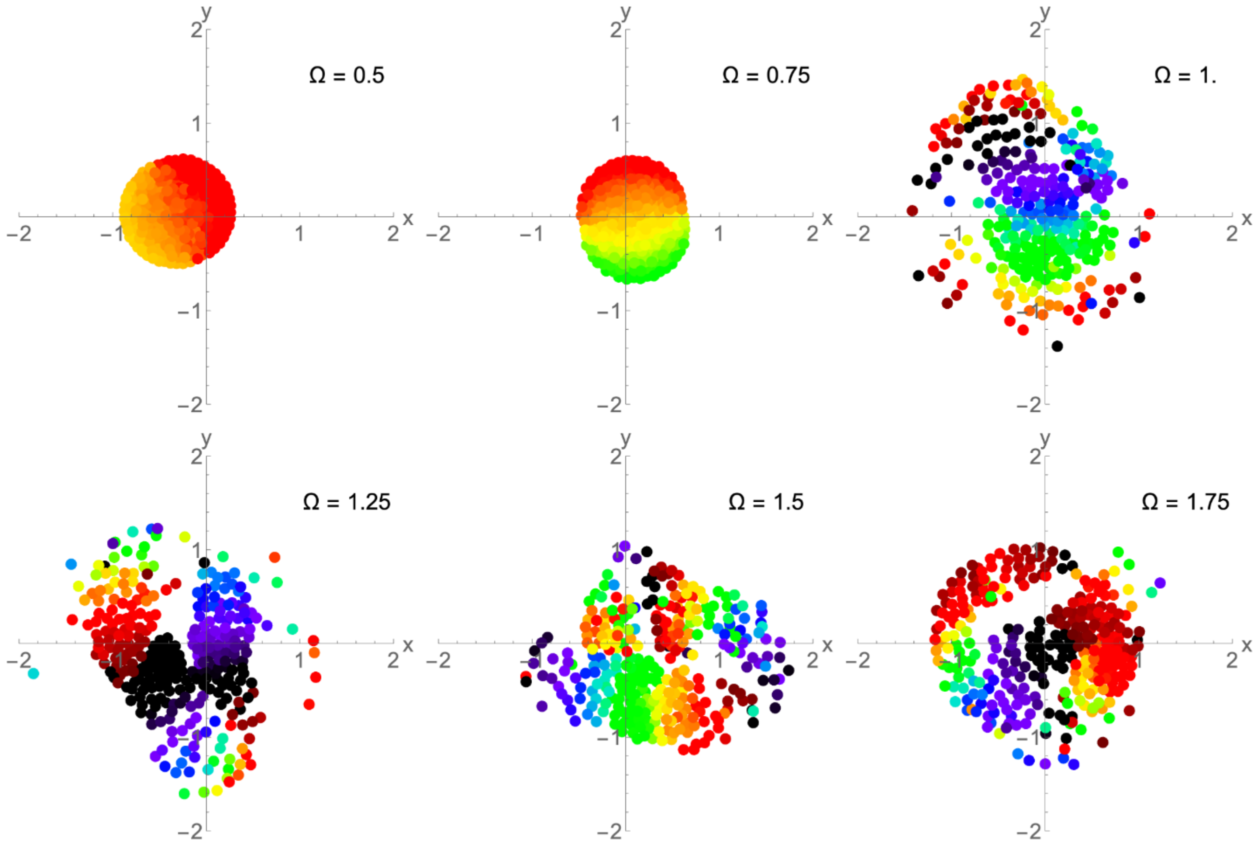


**Supplementary Fig. 44. Sync state for increasing Ω.** As Ω increases, the collective moves away from being synchronized in a cluster to being spatially organized by phase.

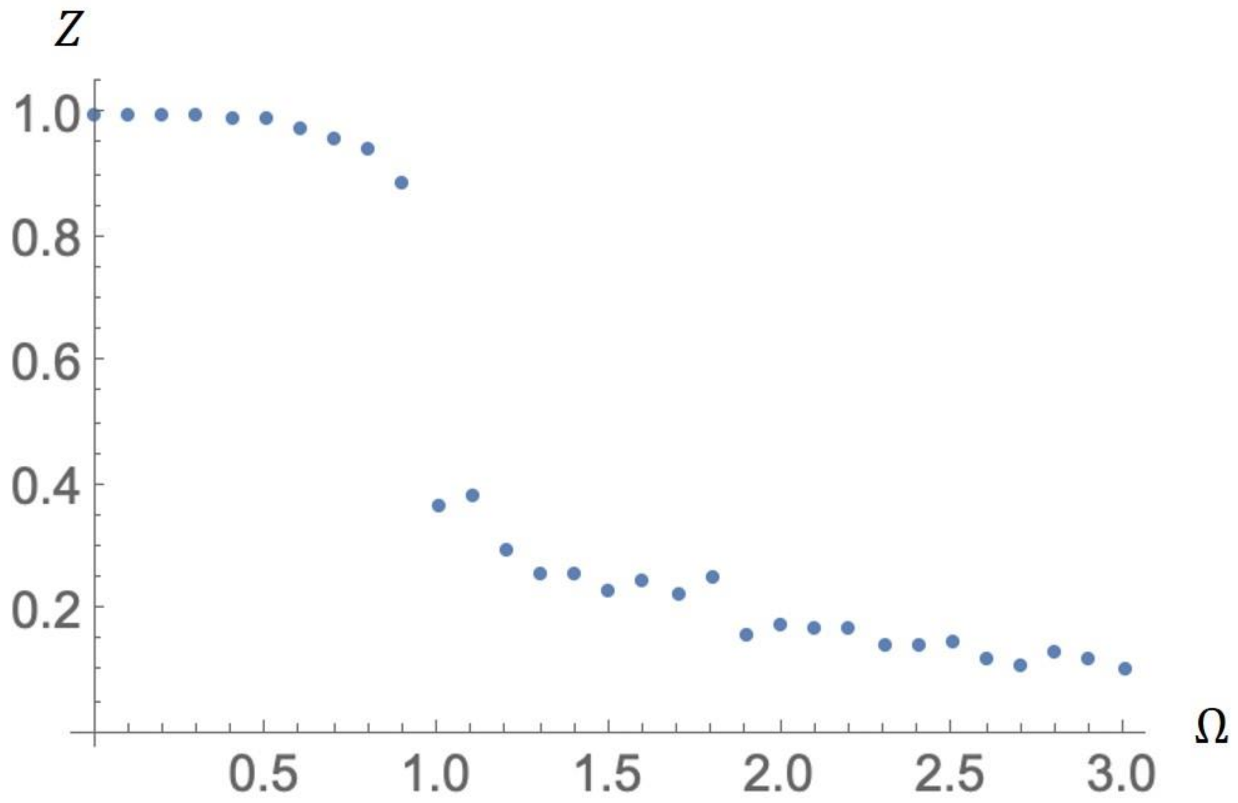


**Supplementary Fig. 45. Phase coherence for increasing $d$ in collectives with global coupling, non-identical, and non-chiral swarmalators.** On the y-axis, $Z$ decreases to almost zero with increasing $d$.

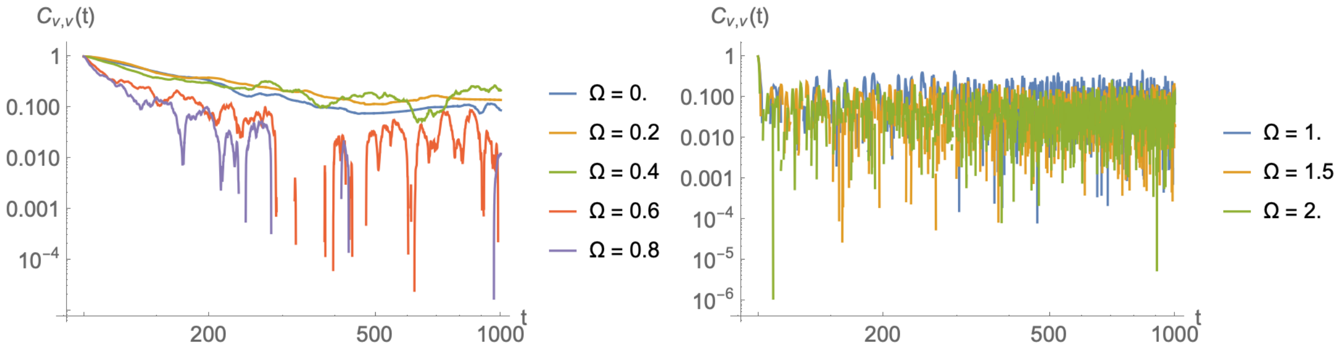


**Supplementary Fig. 46. Non-monotonic behavior of phase coherence for collectives with global coupling, non-identical, and non-chiral swarmalators.** Non-monotonic behavior of $R(d)$ for $\Omega = 1$. Simulation parameters: $(J, K, \sigma) = (1, 1, 10)$, $(dt, T, N) = (0.25, 1000, 200)$. Each data point is the average of five simulations.

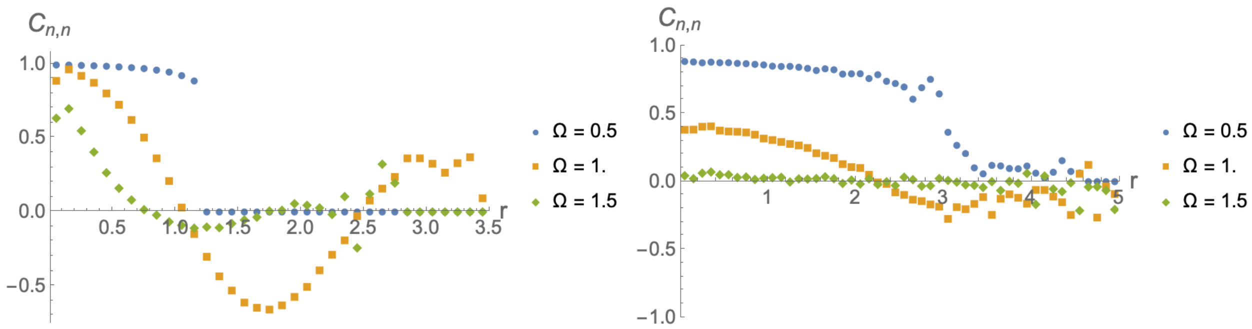


**Supplementary Fig. 47. Phase-phase correlation function for collectives with global coupling, non-chiral, and non-identical swarmalators.** Left: $d = 0$ . Right: $d = 0.5$ . Simulation parameters: $(J, K, \sigma) = (1, 1, 10)$, $(dt, T, N) = (0.25, 1000, 200)$. Each data point is the average of five simulations.

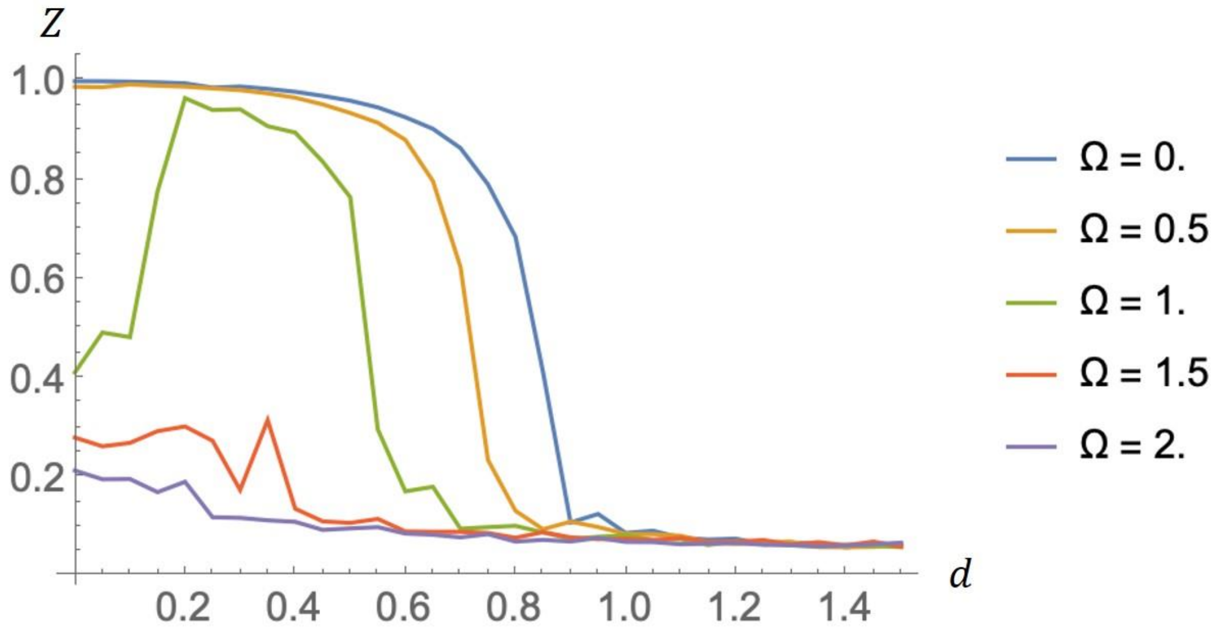


**Supplementary Fig. 48. Non-monotonic behavior of phase coherence for collectives with local coupling and non-chiral swarmalators.** Non-monotonic behavior of $Z(d)$ on the y-axis for $\Omega = 1$. Simulation parameters: $(J, K, \sigma) = (1, 1, 10)$, $(dt, T, N) = (0.1, 500, 300)$. Each data point is the average of five simulations.

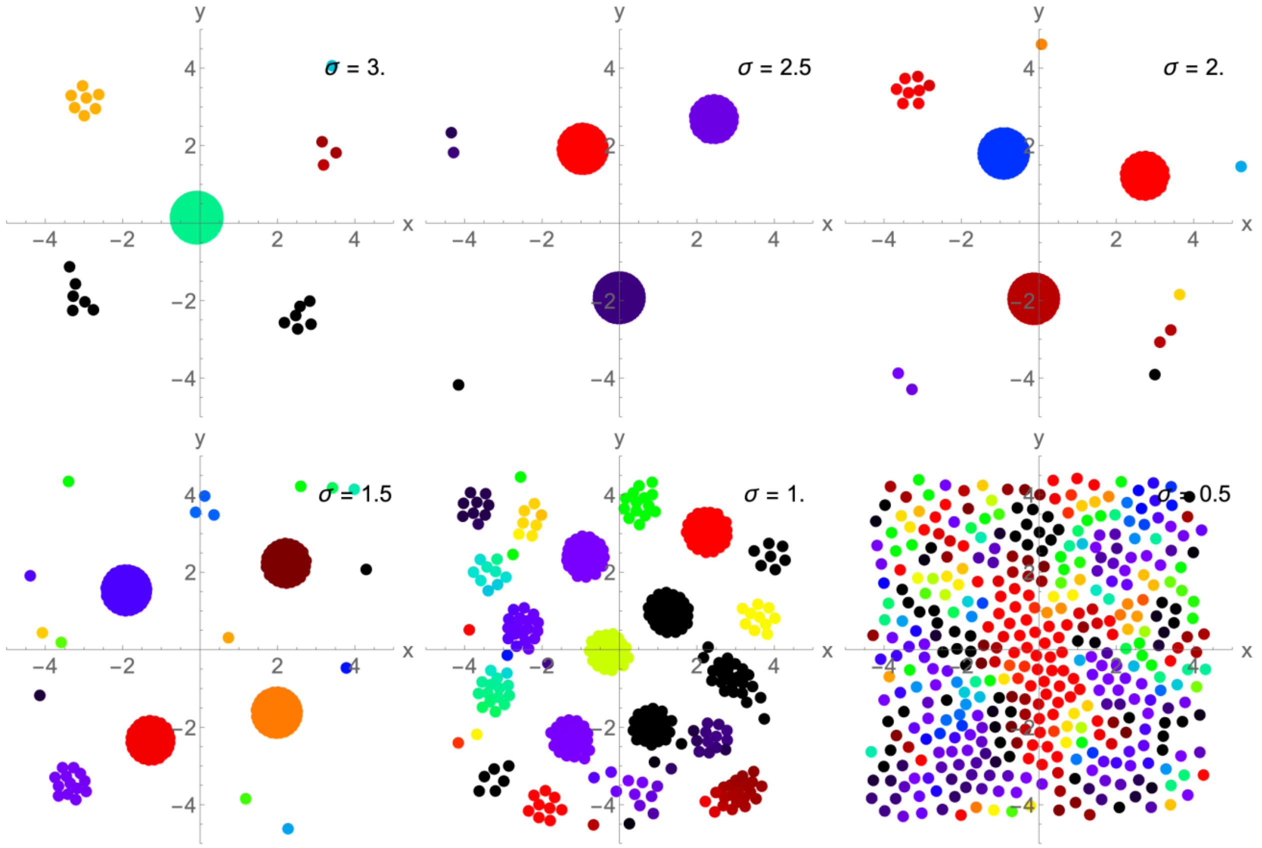


**Supplementary Fig. 49. Sync state for different $\sigma$ when there is local coupling.** Simulation parameters: $(J, K, d, \Omega) = (1, 1, 0.1, 0)$, $(dt, T, N) = (0.25, 200, 400)$. Each swarmalator is initially placed within a box of side length $L = 4$ and its phase is randomly selectred from a uniform distribution between 0 and $2\pi$.

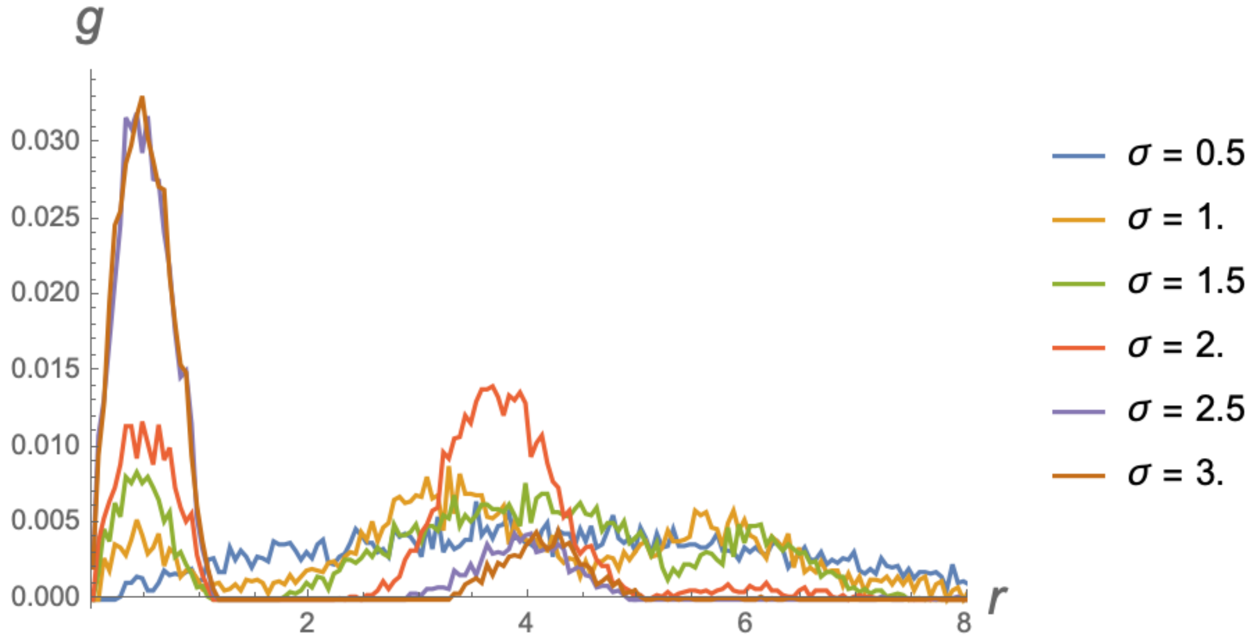


**Supplementary Fig. 50. $g(r)$ for different coupling ranges $\sigma$.** All swarmalators were initially placed randomly within a box of side length $L = 4$.

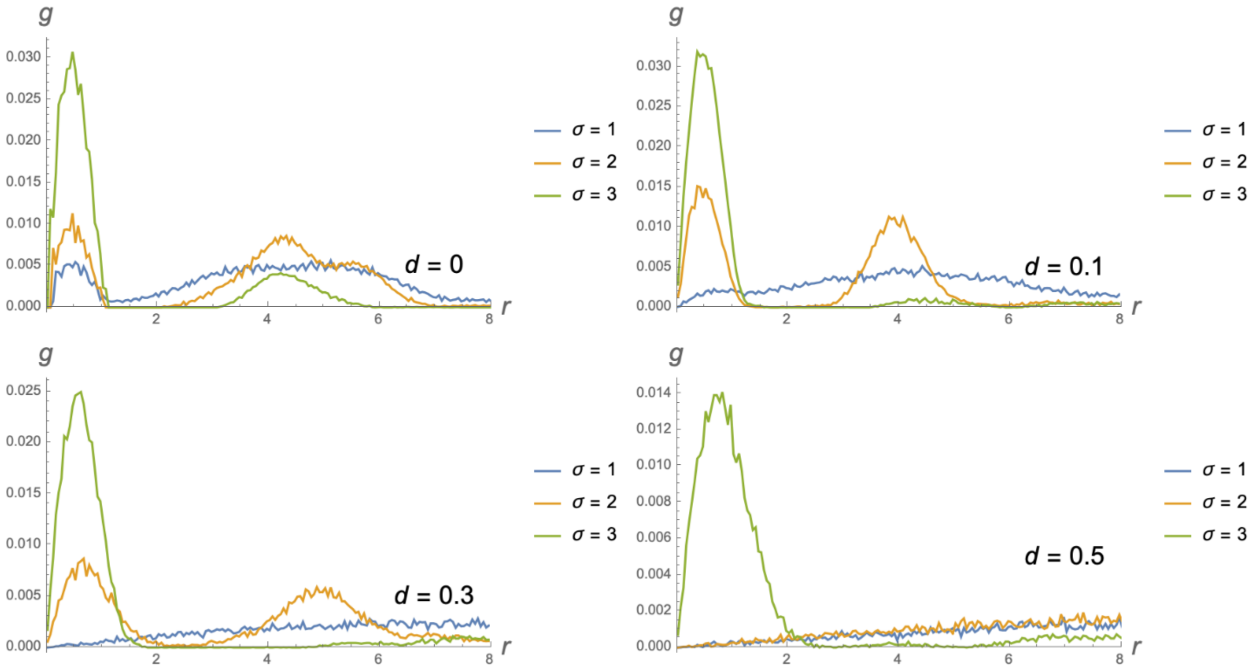


**Supplementary Fig. 51. $g(r)$ for different coupling ranges $\sigma$ when noise increases.** All swarmalators were initially placed randomly within a box of side length $L = 4$.

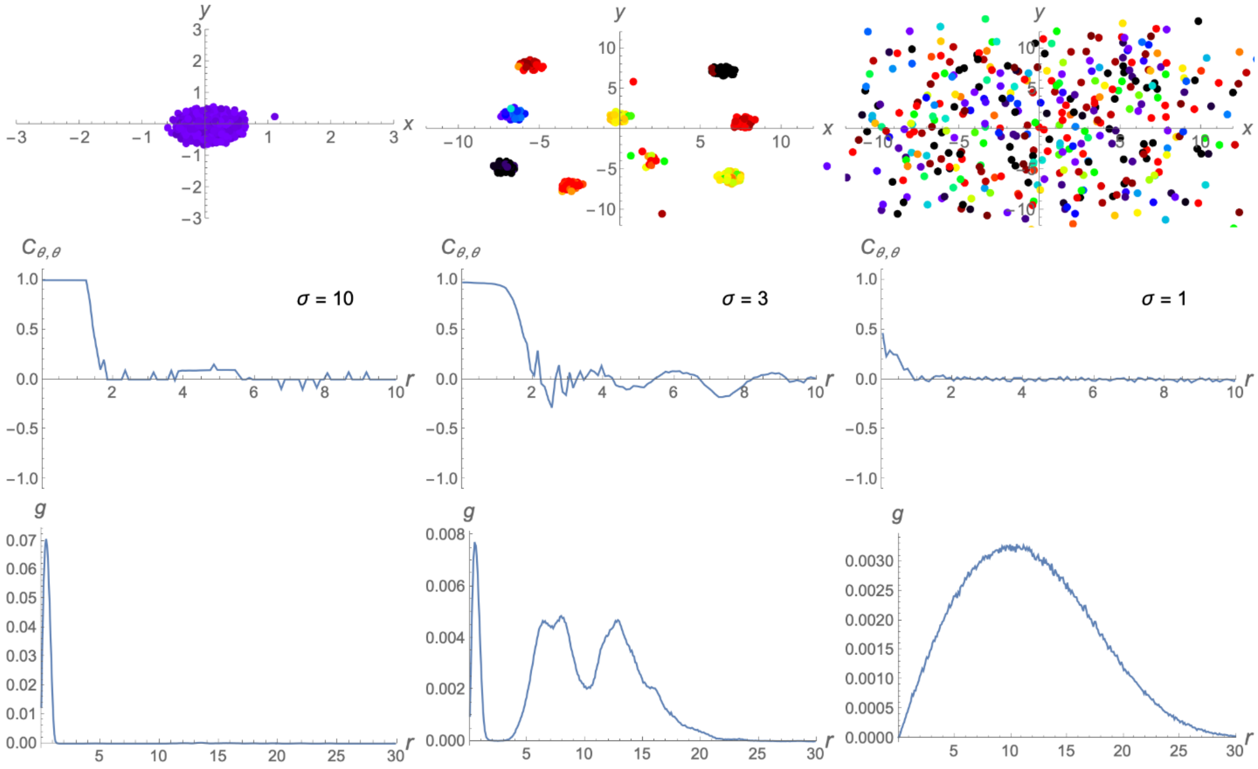


**Supplementary Fig. 52. Phase-phase correlation of collectives with local coupling ($\boldsymbol{\sigma = 10, 3, 1}$), non-identical, non-chiral, and with noise.** Top row: scatter plots of states. Bottom row: phase-phase correlation function for different coupling ranges of $\sigma$. Simulation parameters: $(J, K, d, \Omega) = (1, 1, 0.1, 0.2)$, $(dt, T, N) = (0.25, 500, 300)$. Each data point is the average of 500 simulations.

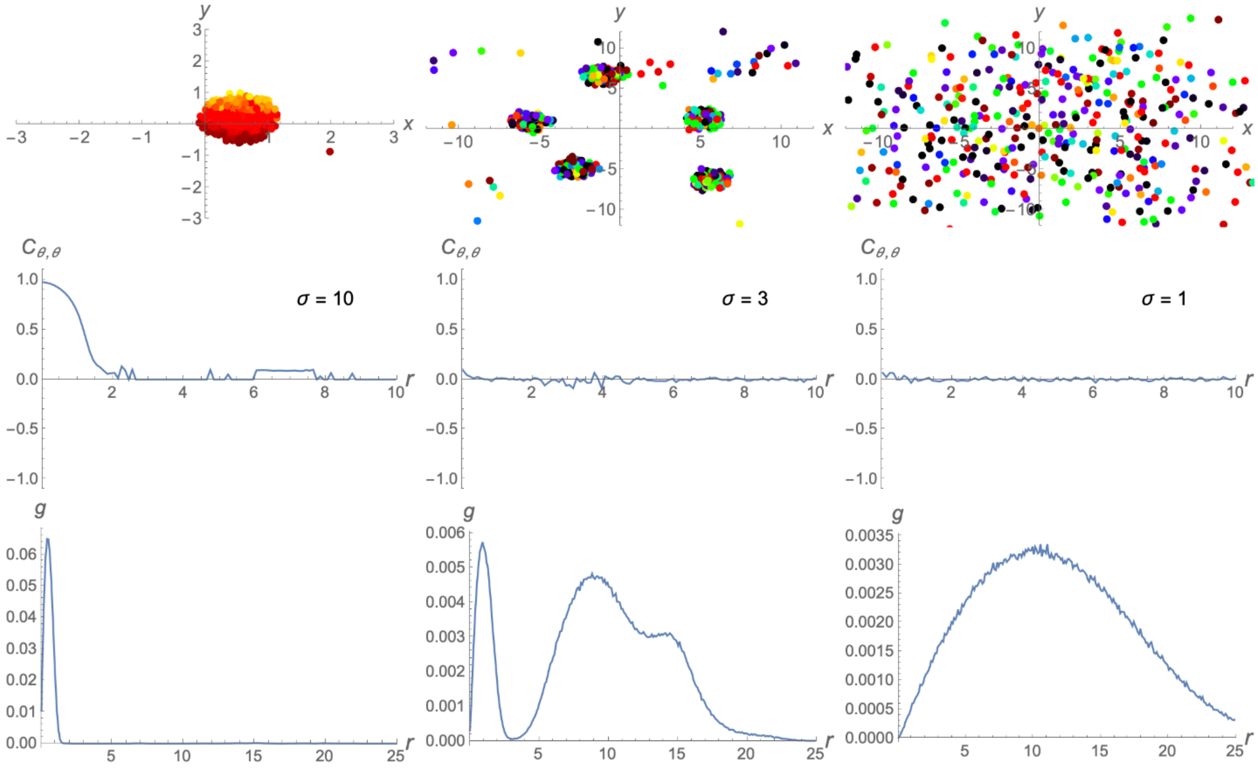


**Supplementary Fig. 53. Phase-phase correlation of collectives with local coupling ($\sigma = 10, 3, 1$), non-identical ($\Omega = 0.9$), non-chiral, and with noise.** Top row: scatter plots of states. Bottom row: phase-phase correlation function for different coupling ranges of $\sigma$. Simulation parameters: $(J, K, d, \Omega) = (1, 1, 0.1, 0.9)$, $(dt, T, N) = (0.25, 500, 300)$. Each data point is the average of 500 simulations.

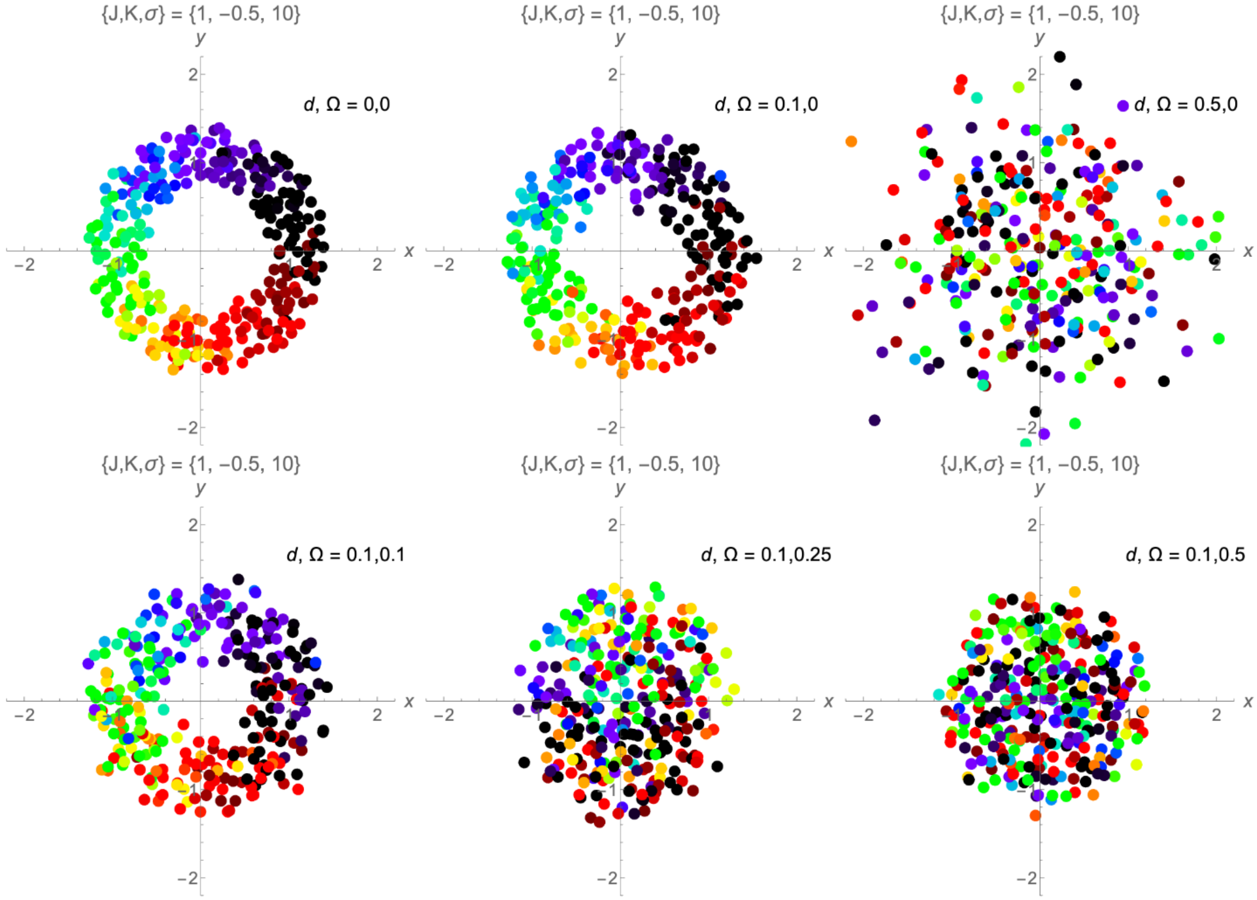


**Supplementary Fig. 54. Vortices with disorder.** Scatter plots of vortices for different amounts of disorder, both active $d > 0$ and quenched $\Omega > 0$.

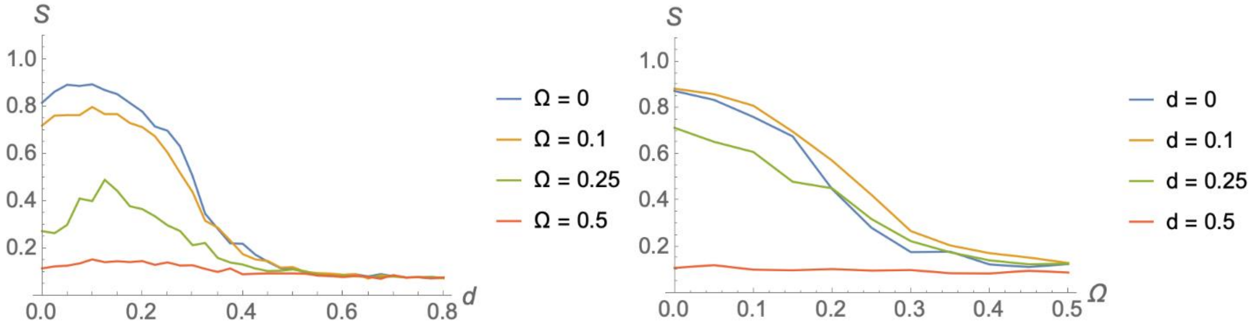


**Supplementary Fig. 55. Order parameter s for different $\boldsymbol{d}$ and Ω.** $\mathrm{S} = \max(\mathrm{S}_+, \mathrm{S}_-)$. Left: $S(d)$ for different Ω. Right: $S(\Omega)$ for different $d$. Simulation parameters: $(J, K) = (0, 1)$.

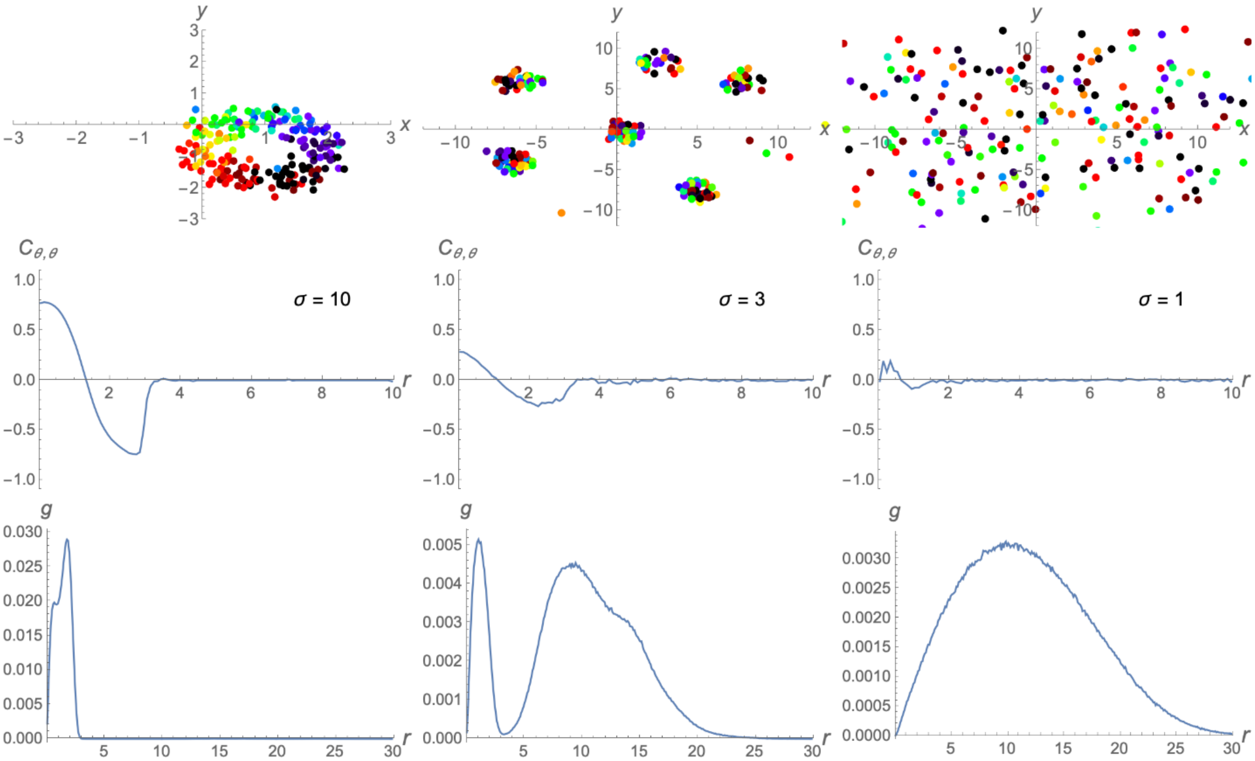


**Supplementary Fig. 56. Phase-phase correlation and $g(r)$ for vortices.** First column: long range coupling regime, with one cluster. Middle row: intermediary coupling regime with multiple vortices. Right row: short range regime, incoherence gas. Simulation parameters: $(dt, T, N) = (0.25, 800, 200)$, $(J, K, d, \Omega) = (1, -0.5, 0.1, 0.2)$. Each data point is the average of 100 simulations.

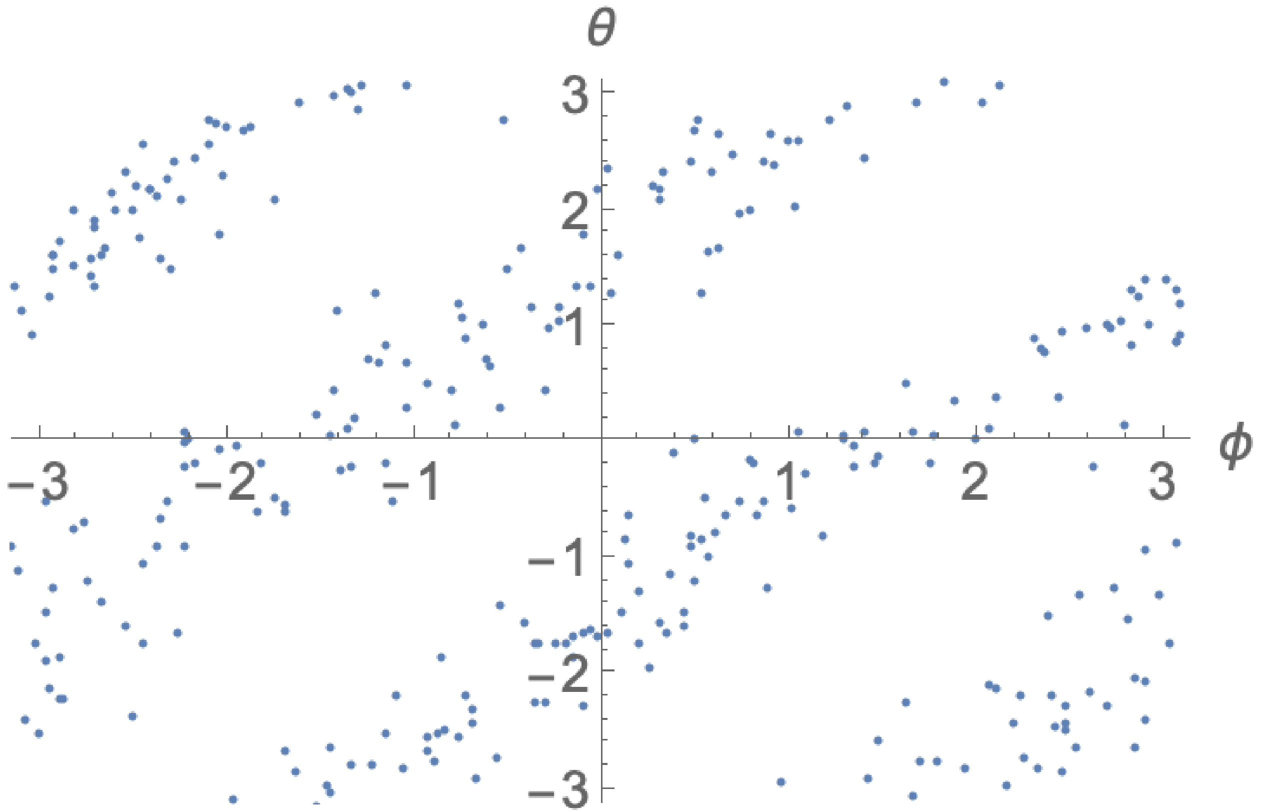


**Supplementary Fig. 57. Phase-phi space for phase wave state.** Scatter plot of phase wave state showing the winding number $k = 2$ . Simulation parameters: $(J, K, \Omega, \sigma, d) = (1, -0.3, 1.5, 10, 0.1)$, $(dt, T, N) = (0.25, 100, 300)$.

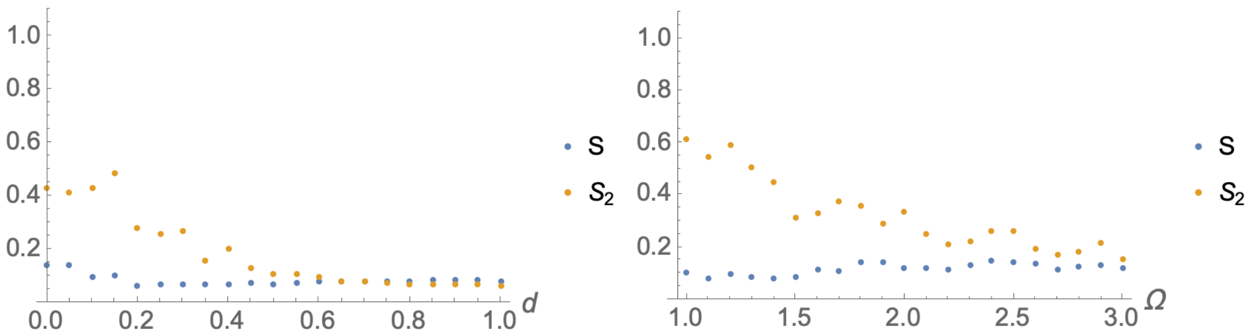


**Supplementary Fig. 58. Order parameters for active and quenched disorder.** Order parameters vs. active disorder (left panel) and quenched disorder (right panel). Simulation parameters: $(J, K, \sigma) = (1, -0.3, 10)$, $(dt, T, N) = (0.25, 100,300)$. (Left) $\Omega = 1.5$. (Right) $d = 0.1$.

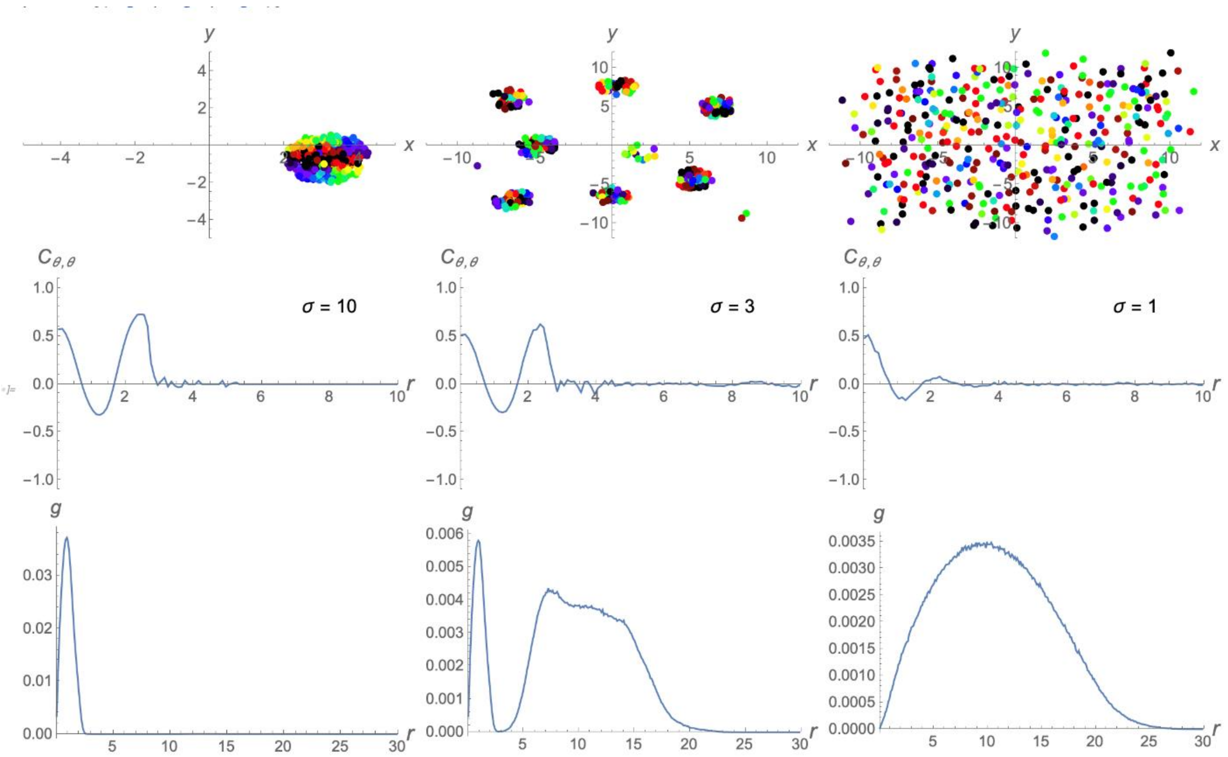


**Supplementary Fig. 59. Phase-phase correlation and $g(r)$ for revolving collectives in the vortex state.** First column: long range coupling regime, with one cluster. Middle row: intermediary coupling regime with multiple vortices. Right row: Short range regime, incoherence gas. Sim pars: (dt, T, N ) = (0.25, 800, 200). Pars: (J, K, d, Ω) = (1, −0.5, 0.1, 3). Each data point is the average of 100 simulations.

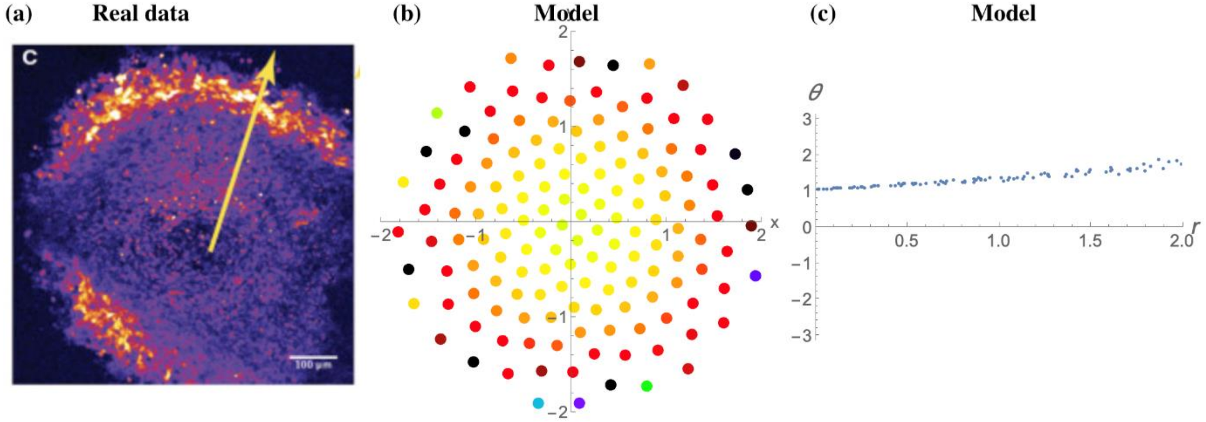


**Supplementary Fig. 60. Comparison to embryonic genetic oscillators. (a)** shows a sketch of the real data[41]. The embryonic cells arrange themselves in disk-like pattern and a wave of genetic activity (highlighted by a bright yellow dye; see paper for details) travels radially outward as indicated by the yellow arrow. **(b)** and **(c)** demonstrate the model reproduces this pattern. See also Supplementary Movie 17.

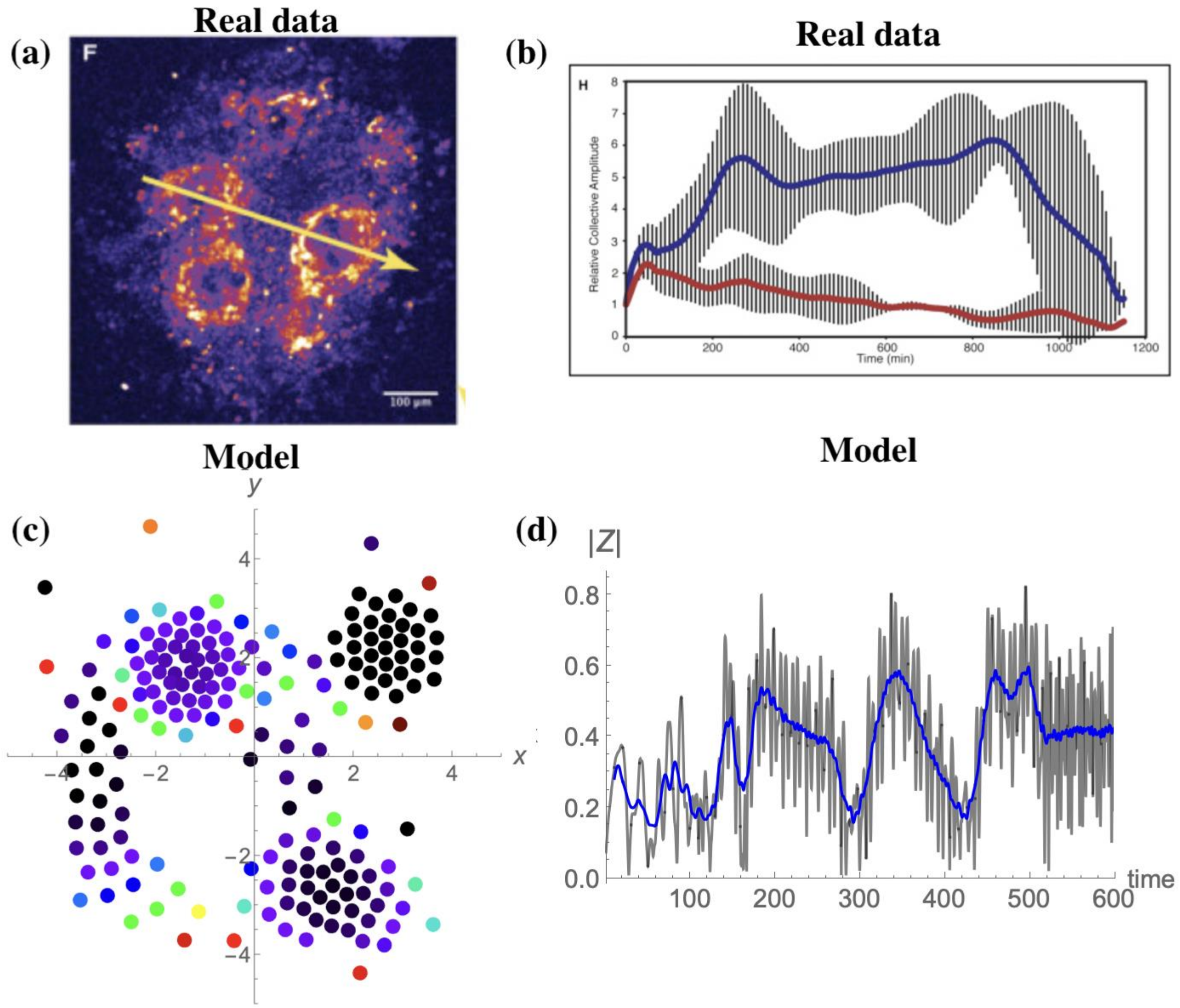


**Supplementary Fig. 61. Local clusters of embryonic genetic oscillators. (a)** shows an experimental scenario where multiple disks are formed[41]. **(b)** shows the degree of collective synchrony in the real data[41] (blue curves; ignore the red curve, it corresponds to a random baseline). **(c)** shows the model reproduces the pattern. **(d)** shows our model again reproduces this pattern qualitatively

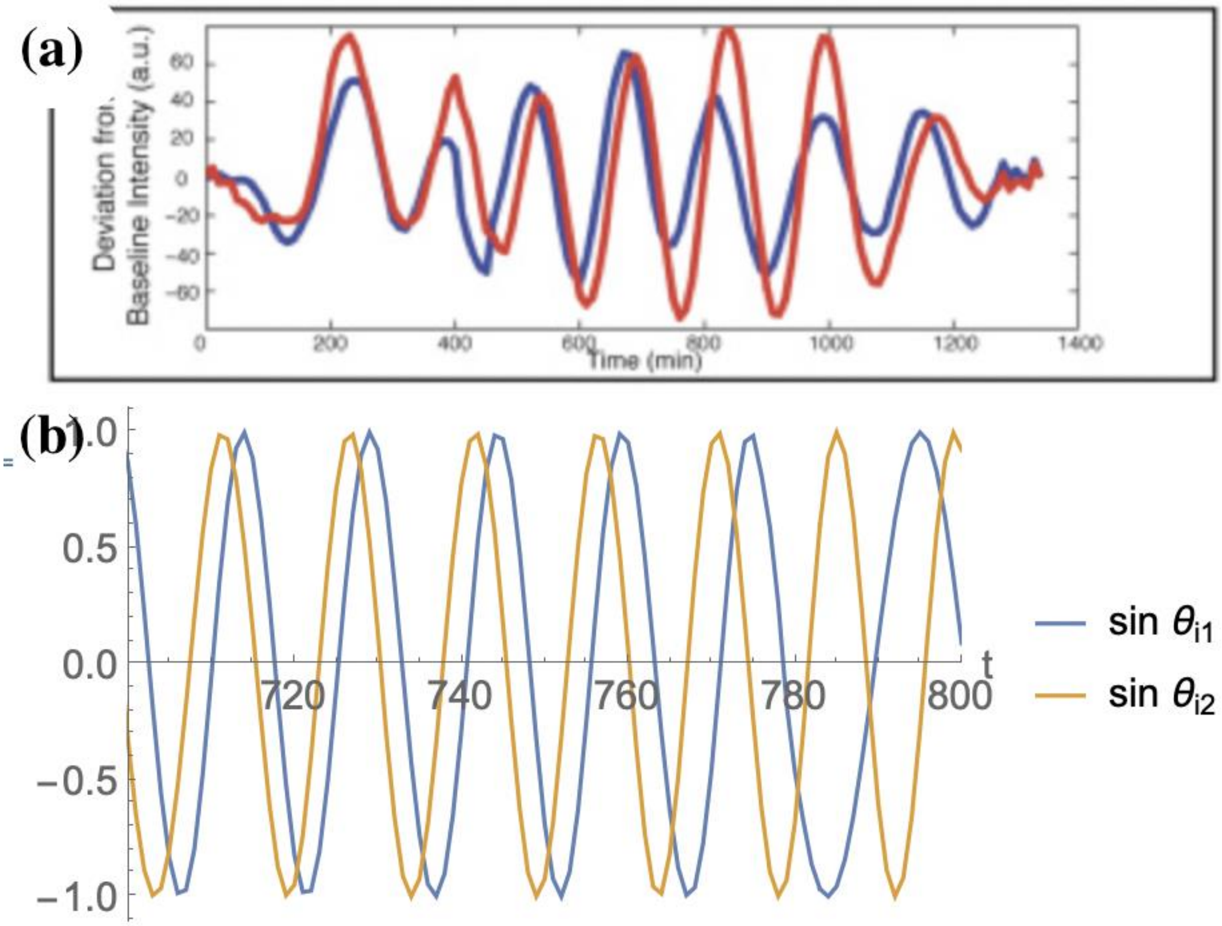


**Supplementary Fig. 62. Synchronization of various clusters. (a)** shows that the different foci from Supplementary Fig. 60 are in sync; the time series for two example oscillator are plotted which are phase locked[41]. **(b)** shows the same curves as produced by the model.

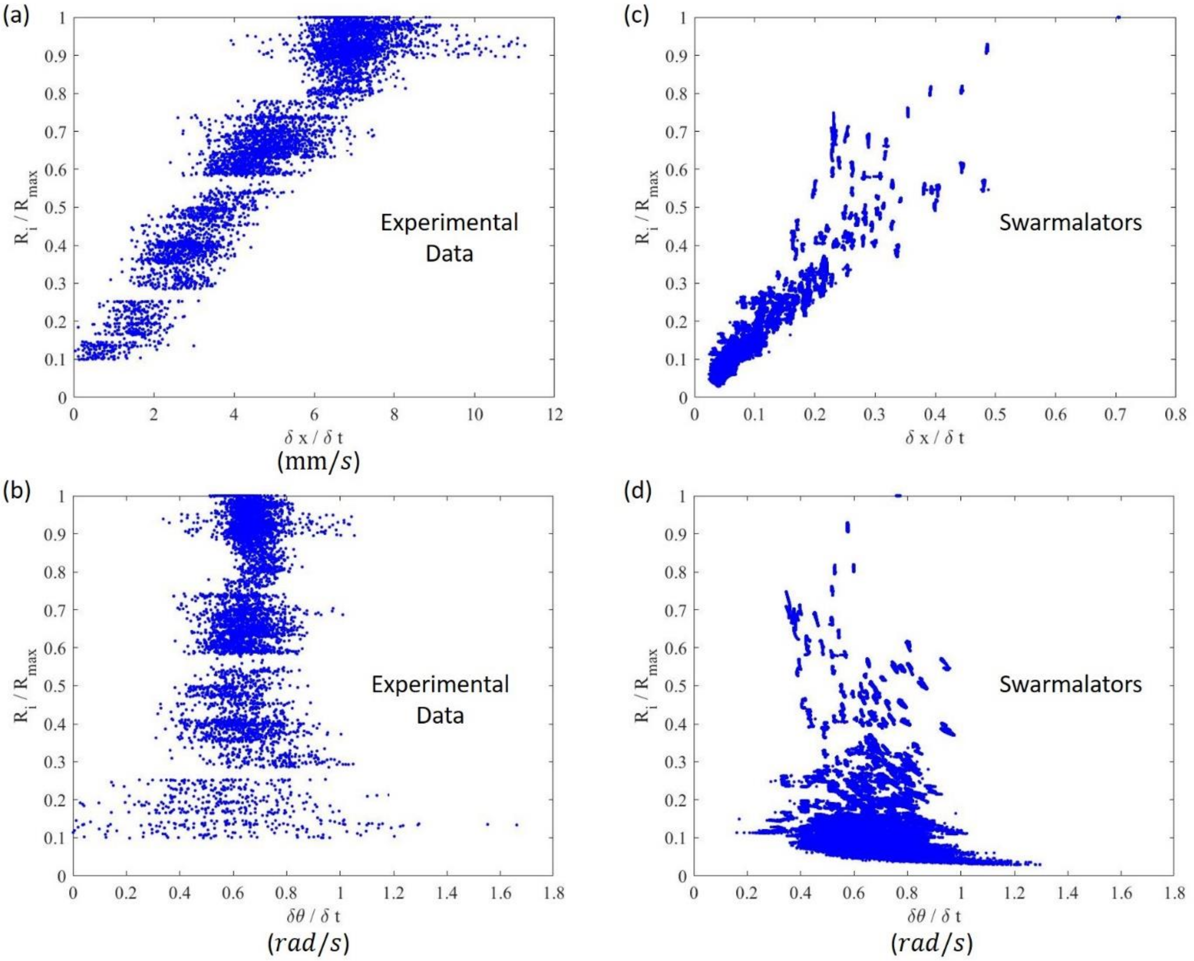


**Supplementary Fig. 63. Comparison to experiments with micro-disks.** Data from the micro-disk experiments conducted in Gardi et al.[50] was used to plot the **(a)** instantaneous speed and the **(b)** instantaneous angular velocity about the collective centroid vs. the normalized distance from collective center. Similar scatter plots **(c-d)** emerge for the swarmalators when the agents' natural frequencies are chosen from an exponential distribution between 0 and 1.

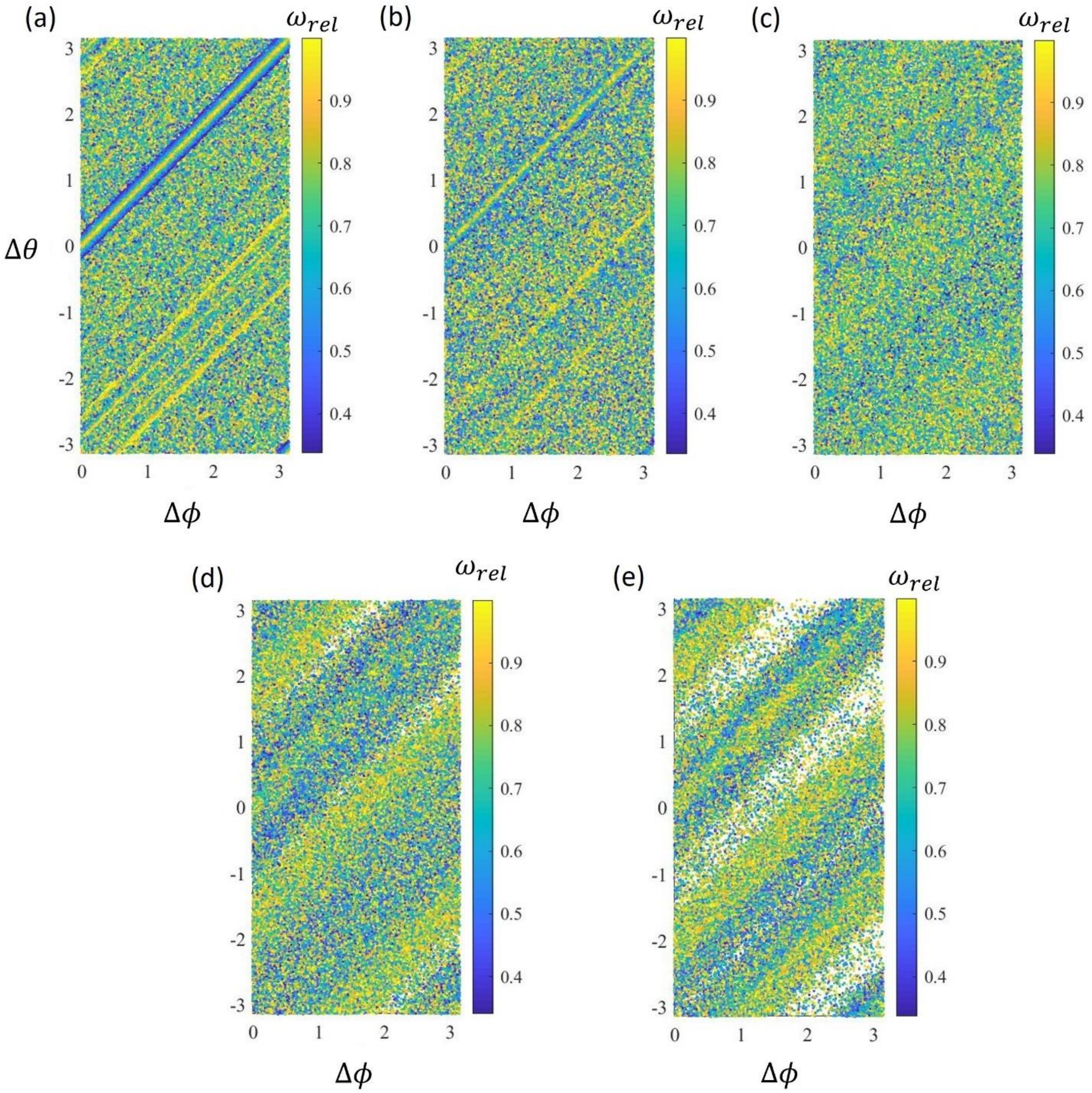


**Supplementary Fig. 64. Phase difference vs. spatial orientation difference.** Data from the micro-disk experiments conducted in Gardi et al.[50] was used to plot the **(a)** instantaneous speed and the **(b)** instantaneous angular velocity about the collective centroid vs. the normalized distance from collective center. Similar scatter plots **(c-d)** emerge for the swarmalators when the agents' natural frequencies are chosen from an exponential distribution between 0 and 1.

## Supplementary Movies

**Supplementary Movie 1. Non-Chiral Swarmalators with No Frequency Coupling.** A collage of movies of non-chiral swarmalators with integer values of $K$ and $J$. The movies corresponding to $F1$ are shown first, followed by $F2$, then $F3$, and finally $F4$.

**Supplementary Movie 2. Splintered Phase Waves.** Collectives with two, three, four, and five natural frequency groups are shown forming splintered phase waves when $K = -0.1, J = 1$.

**Supplementary Movie 3. Natural Frequency Group Separation.** Collectives with three, four, five, and nine natural frequency groups are shown going from a disordered state to exhibiting multiple bouncing clusters when $K = 1, J = 1$.

**Supplementary Movie 4. Concentric Phase Self-Organization.** A movie of a plot of agents' distance from the collective centroid as a function of phase is shown for when there is no chirality, no frequency coupling, $F4$, $K = 2$, and various values of negative $J$.

**Supplementary Movie 5. Non-Chiral Swarmalators with Frequency Coupling.** A collage of movies of non-chiral swarmalators with frequency coupling and integer values of $K$ and $J$ are shown going from a disordered state to their final behavior. The movies corresponding to $F2$ are shown first, followed by $F4$.

**Supplementary Movie 6. Revolving swarmalators with No Frequency Coupling.** A collage of collectives with chirality, no natural frequency-dependent coupling, and integer values of $K$ and $J$ are shown going from a disordered state to their final behavior. The movies corresponding to $F1$ are shown first, followed by $F2$, then $F3$ and finally $F4$.

**Supplementary Movie 7. Revolving Swarmaltors with Frequency Coupling.** A collage of collectives with chirality, frequency coupling, and integer values of $K$ and $J$ are shown going from a disordered state to their final behavior. The movies corresponding to $F2$ and followed by $F4$.

**Supplementary Movie 8. Global coupling, non-chiral, and noise.** Globally coupling non-chiral swarmalators with increasing levels of noise which take the collective further away from a sync state.

**Supplementary Movie 9. Global coupling, non-identical, and non-chiral.** Globally coupling non-chiral swarmalators with increasing spreads of natural frequencies across the collective.

**Supplementary Movie 10. Local coupling, identical, and non-chiral.** Local coupling collectives with different values for radius of interaction with other agents.

**Supplementary Movie 11. Global coupling, non-identical, and non-chiral vortices with noise.** Globally coupling non-chiral swarmalators that form vortices and have increasing spreads of natural frequency and noise.

**Supplementary Movie 12. Global coupling, identical, and non-chiral vortices with noise.** Locally coupling identical and non-chiral swarmalators with various radii of interaction forming vortices.

**Supplementary Movie 13. Local coupling, non-chiral, and no frequency coupling.** A collage of collective with different $\sigma$ are shown for collectives exhibiting no chirality and no natural frequency-dependent coupling. The movies corresponding to $F1$ are shown first, followed by $F2$, then $F3$, and $F4$.

**Supplementary Movie 14. Local coupling, chiral, and no frequency coupling.** A collage of collectives with different $\sigma$ are shown for collectives exhibiting chirality and no frequency coupling. The movies corresponding to $F1$ are shown first, followed by $F2$, then $F3$, and $F4$.

**Supplementary Movie 15. Local coupling, chiral, and frequency coupling.** A collage of locally coupling collectives with different $\sigma$ are shown for collectives exhibiting chirality and frequency coupling. The movies corresponding to $F2$ and followed by $F4$.

**Supplementary Movie 16. Slime Mold.** Locally coupling non-chiral swarmalators that vary the $K$ and $J$ parameters and switch between different states that resemble the various life stages of social slime mold.

**Supplementary Movie 17. Embryonic Genetic Oscillators.** Local clusters are shown to form with radial phase organization; qualitatively similar to the behavior present in embryonic genetic oscillators in the Ref. 41.
