# Supplementary material for: Diverse behaviors in non-uniform chiral and non-chiral swarmalators
Source: Nat Commun. 2023 Feb 20;14:940. doi: 10.1038/s41467-023-36563-4 (PMC9941214; doi:10.1038/s41467-023-36563-4)
Supplement: Supplementary file 3 — Description of additional Supplementary File [file 41467_2023_36563_MOESM3_ESM.pdf]

Descriptions of additional supplementary files

Supplementary Movie 1. Non-Chiral Swarmalators with No Frequency Coupling.
Supplementary Movie 2. Splintered Phase Waves.
Supplementary Movie 3. Natural Frequency Group Separation.
Supplementary Movie 4. Concentric Phase Self-Organization.
Supplementary Movie 5. Non-Chiral Swarmalators with Frequency Coupling.
Supplementary Movie 6. Revolving swarmalators with No Frequency Coupling.
Supplementary Movie 7. Revolving Swarmaltors with Frequency Coupling.
Supplementary Movie 8. Global coupling, non-chiral, and noise.
Supplementary Movie 9. Global coupling, non-identical, and non-chiral.
Supplementary Movie 10. Local coupling, identical, and non-chiral.
Supplementary Movie 11. Global coupling, non-identical, and non-chiral vortices with noise.
Supplementary Movie 12. Global coupling, identical, and non-chiral vortices with noise.
Supplementary Movie 13. Local coupling, non-chiral, and no frequency coupling.
Supplementary Movie 14. Local coupling, chiral, and no frequency coupling.
Supplementary Movie 15. Local coupling, chiral, and frequency coupling.
Supplementary Movie 16. Slime Mold.
Supplementary Movie 17. Embryonic Genetic Oscillators.
